# Large Artificial microRNA Cluster Genes Confer Effective Resistance against Multiple Tomato Yellow Leaf Curl Viruses in Transgenic Tomato

**DOI:** 10.3390/plants12112179

**Published:** 2023-05-31

**Authors:** Annum Khalid, Xi Zhang, Huaijin Ji, Muhammad Yasir, Tariq Farooq, Xinyi Dai, Feng Li

**Affiliations:** National Key Laboratory for Germplasm Innovation and Utilization of Horticultural Crops, College of Horticulture and Forestry Sciences, Huazhong Agricultural University, Wuhan 430070, China

**Keywords:** tomato yellow leaf curl viruses, artificial miRNA, resistance, TY1, whitefly, mixed infection

## Abstract

Tomato yellow leaf curl disease (TYLCD) has become the key limiting factor for the production of tomato in many areas because of the continuous infection and recombination of several tomato yellow leaf curl virus (TYLCV)-like species (TYLCLV) which produce novel and destructive viruses. Artificial microRNA (AMIR) is a recent and effective technology used to create viral resistance in major crops. This study applies AMIR technology in two ways, i.e., amiRNA in introns (AMINs) and amiRNA in exons (AMIEs), to express 14 amiRNAs targeting conserved regions in seven TYLCLV genes and their satellite DNA. The resulting pAMIN14 and pAMIE14 vectors can encode large AMIR clusters and their function in silencing reporter genes was validated with transient assays and stable transgenic *N. tabacum* plants. To assess the efficacy of conferring resistance against TYLCLV, pAMIE14 and pAMIN14 were transformed into tomato cultivar A57 and the resulting transgenic tomato plants were evaluated for their level of resistance to mixed TYLCLV infection. The results suggest that pAMIN14 transgenic lines have a more effective resistance than pAMIE14 transgenic lines, reaching a resistance level comparable to plants carrying the TY1 resistance gene.

## 1. Introduction

RNA silencing refers to sequence-specific gene silencing directed by microRNAs (miRNAs) or small interfering RNAs (siRNAs) 21 to 24 nucleotides long [1]. Plant microRNA genes are non-coding RNA genes and are transcribed by RNA polymerase II. The primary miRNA (pri-miRNA) is capped, spliced and polyadenylated similarly to RNA polymerase II transcribed messenger RNA [2,3]. After transcription, the pri-miRNA is cleaved into miRNA/miRNA* duplex by Dicer-like 1 (DCL1) processing in two or more reactions. The RNA methyltransferase, Hua enhancer 1 (HEN1), then methylates the miRNA/miRNA* duplex, which is then incorporated into the Argonaute protein (AGO), with the mature miRNA strand retained in the RNA-induced silencing complex (RISC) [2,3]. Plant miRNAs play broad roles in regulating plant development and stress responses by silencing the target genes involved in these processes [4,5]. In plants, DCL4 and DCL2 are capable of processing viral double-stranded RNA (dsRNA) into 21–22 nucleotide primary viral siRNAs (vsiRNAs), which are then loaded into AGO1 or AGO2 containing RISC to target viral RNAs for cleavage and degradation [6]. Some of the cleaved viral RNAs may be used by plant RNA-dependent RNA polymerase 1 (RDR1) and RDR6 as templates to produce double-stranded RNAs (dsRNAs), which are then processed by the DCL2 and DCL4 enzymes into secondary vsiRNAs to potentiate antiviral RNA silencing [7]. There is also a class of noncoding RNAs (ncRNAs) which do not fold into a hairpin structure like primary miRNA. These ncRNAs are cleaved by 22 nt miRNAs or miR390 and their cleavage products are transcribed by RDR6 into dsRNAs, which are further cleaved successively into siRNAs in phase with the miRNA cleavage sites [8,9,10,11]. These ncRNA genes are called trans-acting siRNA (TAS) genes and play important roles in regulating plant development and stress responses [4,12].

Owing to its high efficiency and specificity, diverse RNA-silencing mechanisms are used to engineer viral resistance in many plant species [13,14]. Since plant miRNA genes produce mature miRNAs with defined sequences, artificial miRNA genes can be created using a plant miRNA gene as backbone by replacing its mature miRNA sequences with sequences complementary to a plant gene or viral sequences and replacing its miRNA* sequences concomitantly to maintain its secondary structure [15,16]. Artificial microRNA (AMIR) technology has been applied to viral disease resistance for more than a decade and resistance against one or two viral pathogens has been achieved in transgenic plants expressing one or two AMIRs [15,17,18,19,20,21]. AMIR-mediated resistance has several advantages over traditional transgene-based viral-resistance approaches using long antisense viral transcripts or long viral dsRNAs, such as reducing off-target effects in host genes and reducing the risk of recombination with non-target viruses [14]. 

Tomato is an important vegetable crop cultivated worldwide and its production is affected by many viral diseases [22,23,24]. Tomato yellow leaf curl disease (TYLCD) is one of the major tomato diseases. TYLCD is caused by tomato yellow-leaf-curl-virus-like viruses (TYLCLVs), which are a complex of begomoviruses that includes ten definitive species [25]. TYLCLVs are transmitted by whiteflies and cause extensive yield losses in tomato plants, up to 100% depending on the stage of plant growth at the time of infection [26]. Breeding efforts have introgressed several resistance loci from wild tomato species into cultivated tomato. Among these, *Ty1* and *Ty3* have been cloned and found to be allelic encoding RNA-dependent RNA polymerases (RDRs) homologous to Arabidopsis RDR3/4/5, and they function by methylating viral genomes [27]. The hairpin-RNA-based RNA silencing strategy has been applied to engineer TYLCV resistance in tomato [28]. However, artificial miRNA technology has not been used to engineer resistance to multiple TYLCLV species in a simultaneous manner.

This work analyzed a large number of TYLCLVs and satellite sequences and identified conserved sequences in their protein-coding sequences. Fourteen artificial miRNA (AMIR) genes were designed to target seven viral genes, with two AMIR for each viral gene. The function of each AMIR gene was validated via transient assays, and they were clustered in two ways to form AMIR superclusters to silence multiple TYLCLVs. In the AMIR-in-exon (AMIE) method, a dual AMIR cluster for each viral gene was concatenated in a single transcript without intron; thus, they were processed by DCL1 together. In the AMIR-in-intron (AMIN) method, each dual AMIR cluster was enclosed in an intron derived from an endogenous gene such that each AMIR cluster was processed individually by DCL1 after splicing. Stable transgenic tomato plants were created using both AMIE and AMIN superclusters, and infectious clones for seven TYLCLV species were also created. The resistance to mixed TYLCLV infection was tested in both growth room and plastic house conditions using Agrobacterium-mediated or whitefly-mediated infections. Our results showed that the AMIE supercluster confers effective resistance to mixed TYLCLV infections at levels comparable to the TY1 gene, and the AMIN supercluster confers slightly better resistance than AMIE and TY1.

## 2. Results

### 2.1. Identification of Conserved Target Sites in TYLCLV Genomes and Design of Artificial miRNA Precursors

**Figure 1:**
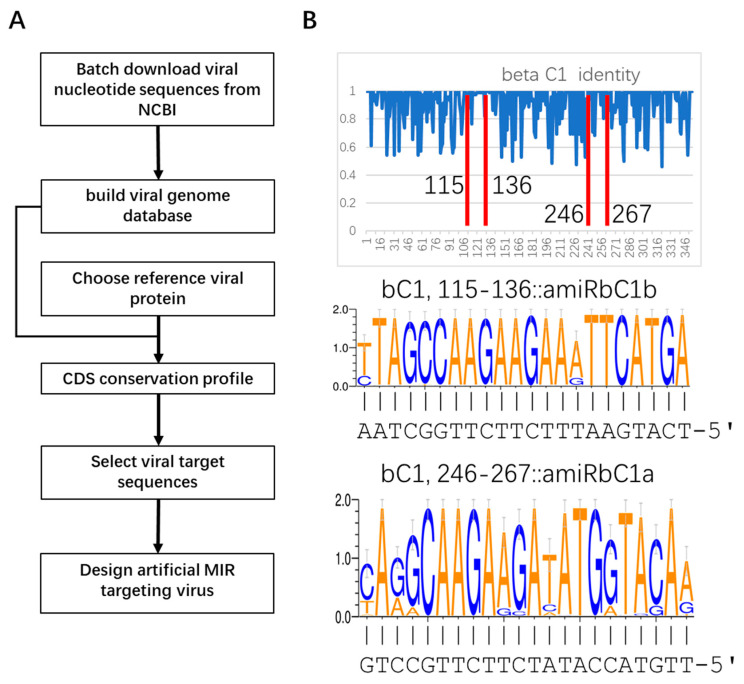
Establishment of a method to design artificial miRNA targeting conserved viral sequences. (**A**) Method for designing AMIR precursor targeting conserved viral coding sequences. (**B**) Identification of conserved target sites in TYLCV-satellite-encoded βC1 gene. The nucleotide conservation profile of βC1 gene-coding sequences is shown in the top panel. Alignments between the designed amiRbC1a/b and the selected target sequences are shown in the middle and bottom panels.

To identify the conserved protein-coding region in the TYLCLV genomes, TYLCLV genome sequences were retrieved from the NCBI Nucleotide database using the keywords “tomato yellow leaf curl virus”, which retrieved 1853 TYLCLV sequences, including 79 β satellite sequences (Supplemental Data S1). Sequences of TYLCLV genes encoding CP, V2, C1, C2, C3 and C4—and those of the β satellite encoding βC1—proteins were extracted from these genomes. Then, multiple sequence alignments were conducted. The analyses identified several conserved peptide-coding sequences in these viral genes (Figure 1 and Figure S1).

Two conserved peptide-coding sequences were selected for each viral protein as artificial miRNA target sequences. Two 22 nt target sequences were designed for each gene to enhance silencing efficiency by 22 nt miRNA-triggered phasiRNA synthesis from targeted viral transcripts [29]. To facilitate RISC loading of the designed mature miRNA strand, the last nucleotides of the target sequences were selected to be “A” or “G” so that the 5′ end of the mature strand of the designed miRNA could be a “U” (Figure 1B and Figure S1), which favors the mature strand in binding to AGO1 during RISC assembly [8,30].

We used the AMIRdesigner webserver to design the AMIR precursor [31]. This server used the Arabidopsis MIR171 and MIR164 precursors as the basic backbones. Several MIR171 and MIR164 derivatives were created by mutating the basic backbones, which maintained their foldback structure. Diversified backbone sequences made it easier to make AMIR clusters via fusion polymerase chain reaction. When the input mature miRNA length was 22 nt, an asymmetric bulge was introduced to a miRNA/miRNA* duplex, which produced 22 nt rather than 21 nt mature miRNAs via DCL1 processing [10,11]. With the AMIRdesigner webserver, two AMIRs targeting each TYLCV and its satellite-encoded gene were designed using the MIR171 and AMIR164 basic backbones, and they were joined to form a dual AMIR cluster (Supplemental Data S2). 

### 2.2. Synthesis of Artificial microRNA Clusters Encoded in Exon (AMIE) and in Intron (AMIN) Targeting TYLCV Proteins 

**Figure 2:**
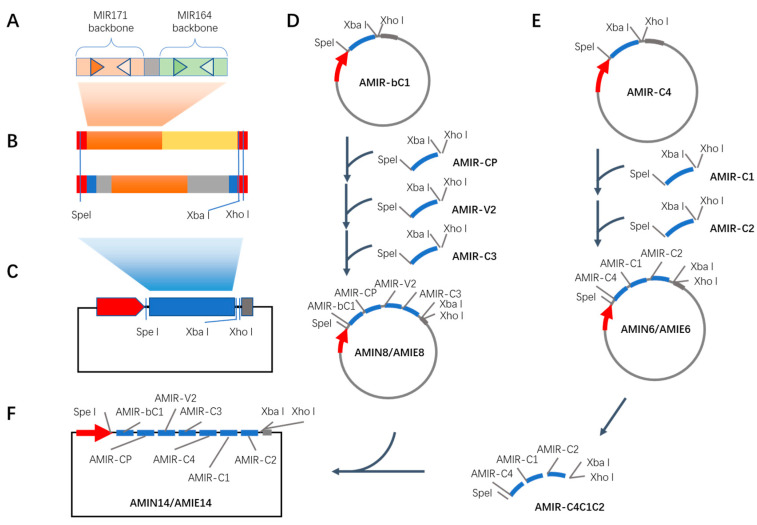
Construction of an expression vector for artificial miRNA encoded in exon (AMIE) and in intron (AMIN). (**A**) Structure of dual miRNA cluster. Orange and green boxes represent MIR171 and MIR164 precursor backbones, respectively. Gray box represents connector sequences. Brown and green triangles represent mature artificial miRNA a and b, while the open triangles represent the corresponding miRNA*. (**B**) Structures of exon-encoded (AMIE, top) and intron-encoded (AMIN, bottom) dual miRNA clusters. Red boxes represent indicated enzyme restriction sites. Brown boxes represent dual miRNA cluster shown in A. Yellow box represents fragments of GUS-coding sequences. Gray boxes represent intron sequences from PDS gene while blue boxes represent short exon sequences flanking each intron. (**C**) Structure of expression vector for a dual miRNA cluster. Red box represents 35S promoter sequence. Blue box represents AMIE or AMIN targeting 7 TYLCV proteins, the structure of which is shown in B. Gray box represents 35S terminator sequence. (**D**) Method to clone eight-miRNA expression vectors pAMIN8 and pAMIE8. (**E**) Method for cloning six-miRNA expression vectors pAMIN6 and pAMIE6. (**F**) Method for cloning fourteen-miRNA expression vectors pAMIN14 and pAMIE14. At each cloning step, vector DNA was digested with XbaI and XhoI, while insert DNA was digested with SpeI and XhoI.

Seven MIR171-based AMIRs and seven MIR164-based AMIRs were synthesized via fusion PCR [31]. Overlapping sequences of 18 nt were added to the 5′ end of the P5 primer for the MIR171-based AMIR and the P1 primer for the MIR164-based AMIR (Supplemental Table S1), such that two AMIRs targeting the same viral gene could be further joined into a dual AMIR cluster via fusion PCR (Figure 2A). Next, the dual AMIR cluster was fused with a *GUS* gene fragment of about 200 nt or was flanked with about 200 nt *PDS* exon–intron and intron–exon junction sequences to make seven AMIE and AMIN constructs. These constructs contained a Spe I restriction site on the 5′ end and Xba I/Xho I restriction sites at the 3′ end of the AMIR inserts (Figure 2B). Fourteen of these constructs were cloned into an expression vector via ligation-independent cloning to obtain AMIE and AMIN vectors targeting individual viral genes: pAMIE-C1, pAMIN-C1, pAMIE-C2, pAMIN-C2, etc. (Figure 2C; Materials and Methods; Supplemental Data S2). 

To make higher-order AMIR clusters, AMIRs from pAMIE-CP, pAMIE-V2 and pAMIE-C3 were cloned into the pAMIE-bC1 vector sequentially to create a larger AMIR cluster with eight AMIRs targeting four viral genes (bC1, CP, V2 and C3) using restriction enzyme digestion and the ligation approach, which was designated pAMIE8 (Figure 2D). In the cloning process, the recipient vector was digested with XbaI and XhoI while the incoming insert was released from its parent vector using SpeI and XhoI. After ligation, the XbaI and XhoI restriction sites were restored in the resulting vector, which was ready to take new inserts using the same process (Figure 2D and Figure S2). Similarly, pAMIN8 was created with intron-encoded AMIRs targeting bC1, CP, V2 and C3 (Figure 2D) and pAMIE6 and pAMIN6 were created to target C4, C1 and C2 with exon-encoded and intron-encoded AMIRs, respectively (Figure 2E). Finally, AMIR clusters were released from pAMIE6 and pAMIN6 with SpeI and XhoI and ligated into XbaI- and XhoI-digested pAMIE8 and pAMIN8 to create pAMIE14 and pAMIN14 vectors, respectively, both of which can target all seven viral genes (Figure 2F).

### 2.3. Validation of amiRNA Expression from AMIE and AMIN Constructs

**Figure 3:**
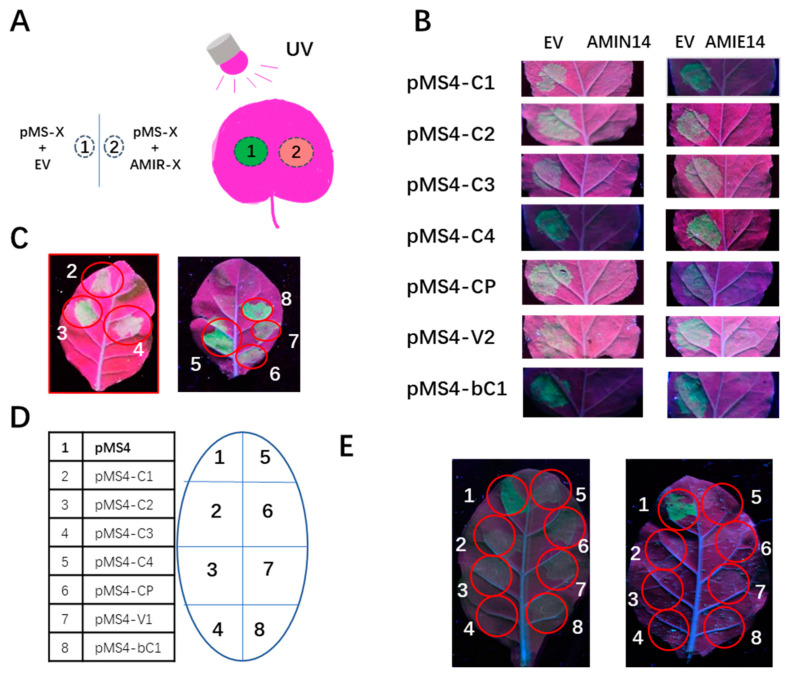
Validation of artificial miRNA expression in transient assay and transgenic tobacco plants using miRNA sensor constructs. (**A**) Diagram shows the combination of agrobacteria infiltration in each leaf of N. benthamiana (left) and the predicted outcome under UV light. (**B**) Green florescence image of N. benthamiana leaves under UV light. Each patch was infiltrated by agrobacteria harboring an empty vector (EV) or a miRNA expression vector (AMIN14 or AMIE14), as indicated on the top, and miRNA sensor constructs, as indicated to the left. (**C**) Expression of seven miRNA sensors in wild-type N. tabacum leaves (top; the # in the red circle corresponds to the # listed in the table in (**D**)). (**D**) Infiltration scheme on transgenic AMIN14 and AMIE14 leaves (bottom). (**E**) Green florescence image of infiltrated leaves of AMIN14 (left) and AMIE14 (right) transgenic plants.

The expression of the designed amiRNAs from pAMIE and pAMIN vectors was first validated with a miRNA sensor assay using agrobacterium-mediated transient expression in *N. benthamiana* plants [32]. A miRNA sensor construct was created by inserting a miRNA-binding site into ER-targeted GFP-coding sequences, which produced green florescence protein when expressed with an empty vector, resulting in green florescence in the infiltrated patch under UV light. When the miRNA sensor construct was co-expressed with the cognate miRNA expression construct, the expression of GFP was inhibited, and no green florescence could be detected under UV light (Figure 3A and Figure S3A). For this purpose, seven miRNA sensor constructs with amiRNA-binding sites were constructed and named pMS4-C1, pMS4-C2, pMS4-C3, pMS4-C4, pMS4-CP, pMS4-V2 and pMS4-bC1 (Supplemental Data S3). Seven pAMIE constructs and seven pAMIN constructs, each containing a dual AMIR cluster that targeted a single viral gene, were tested with their cognate pMS4 sensor constructs. The transient expression of each pMS4 sensor with an empty vector resulted in strong green florescence, and green florescence inhibition was observed when it was co-expressed with the pAMIN or pAMIE vectors (Figure S3B, left vs. right), demonstrating that both intron- and exon-encoded amiRNAs are expressed and functional in repressing GFP expression. The pAMIN14 and pAMIE14 vectors were tested against each of the seven pMS4 sensor constructs. Figure 3B shows that strong green florescence was observed in the patch infiltrated with each pMS4 sensor construct and empty vector while no green florescence was observed in the patch where the pMS4 sensor was co-expressed with either the pAMIN14 or pAMIE14 vectors, indicating both AMIN14 and AMIE14 produce amiRNAs capable of targeting all seven viral targets, similar to dual clusters.

To further characterize amiRNA production from the AMIN and AMIE constructs, fourteen dual amiRNA clusters, pAMIN6, pAMIE6, pAMIN8, pAMIE8, pAMIN14 and pAMIE14 were transformed into *N. tabacum* SR1 plants, and pAMIN14 and pAMIE14 were also transformed into *Solanum lycoperscicum* A57, to produce stable transgenic plants. To validate amiRNA expression from positive transgenic tobacco plants using the seven miRNA sensors, the expression of these miRNA sensors was first tested in wild-type *N. tabacum* SR1 plants. The results showed that all these miRNA sensors were expressed in SR1 plants via agrobacterium-mediated transient expression (Figure 3C). To test transgenic lines of the AMIN14 and AMIE14 constructs, the miRNA sensor constructs, together with a non-target control pMS4 construct, were transiently expressed in the young leaves of these transgenic plants (Figure 3D). The results showed that green florescence was only found in patches infiltrated with the non-targeted pMS4 construct; none of the patches infiltrated with the targeted pMS4 sensors showed green florescence under UV light (Figure 3E). The expression of amiRNAs from the dual amiRNA cluster, pAMIN6, pAMIE6, pAMIN8 and pAMIE8 transgenic lines were also confirmed with the same method (Figure S3C–E). These results showed that all the selected transgenic lines were capable of producing the designed functional amiRNAs.

### 2.4. Synthesis of TYLCV Infectious Clones and Validation of Their Infectivity in N. benthamiana

Tomato yellow leaf curl disease is the most destructive tomato crop disease, and it can cause up to 100% yield loss. According to the literature, there are 10 species of tomato yellow-leaf-curl-virus-like viruses (collectively named TYLCLV hereafter) [25]. To test the TYLCD resistance conferred by the designed AMIR, we prepared infectious clones of these viruses based on their genomic information in the NCBI Database (Supplemental Table S2). 

**Figure 4:**
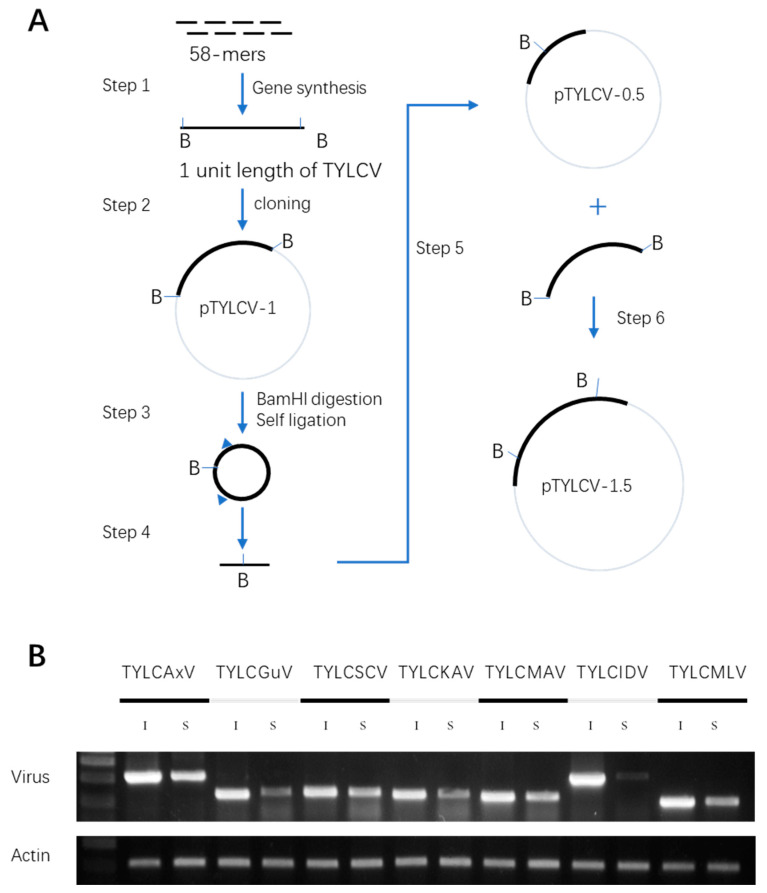
TYLCV infectious clone construction and validation. (**A**) Workflow for TYLCLV infectious clone construction. (**B**) PCR analysis of different TYLCV species in inoculated leaves (I) and systemic leaves (S) of N. benthamiana plants.

The infectious clone was prepared according to the previous study [33]. A 1.5-unit tandem repeat was cloned into the T-DNA region of a binary vector. For the preparation of the one-unit repeat, a 2.8kb fragment for all TYLCLV infectious clones was prepared using the gene synthesis approach [34], and it contained the full genome of the virus (see Method and Figure 4 Step 1). This one-unit length TYLCLV was cloned into the pHLic14 vector (Figure 4 Step 2). After successful cloning into the vector, the one-unit vector was digested using BamHI, self-ligated, and amplified with primers, producing a 2.1kb fragment. This 2.1kb fragment was linearized and cloned into the pHLic14 vector (Figure 4 Steps 3–5). Finally, the full-length insert (one unit) was released from vector I with BamHI, recovered by gel purification (2.8 kb band) and cloned into the BamHI site of vector II to generate the final infectious clone (Figure 4 Step 6). The same procedure was repeated for all seven TYLCLV clones. 

To validate the infectivity of the TYLCLV infectious clones, the above vectors were transformed into Agrobacterium and introduced into *N. benthamiana* plants via Agrobacterium-mediated infiltration. DNA samples were prepared from both infiltrated leaves and systemic leaves at 7 days post infiltration. Different species of TYLCLVs were analyzed using species-specific primers, and the results showed that all TYLCLVs were detected in both inoculated and systemic leaves (Figure 4B), indicating that they were infectious.

### 2.5. The AMIN Strategy Confers Better Resistance against Mixed Infections by Multiple TYLCLV Species in Transgenic Tomato Plants in Growth Room Conditions

**Figure 5:**
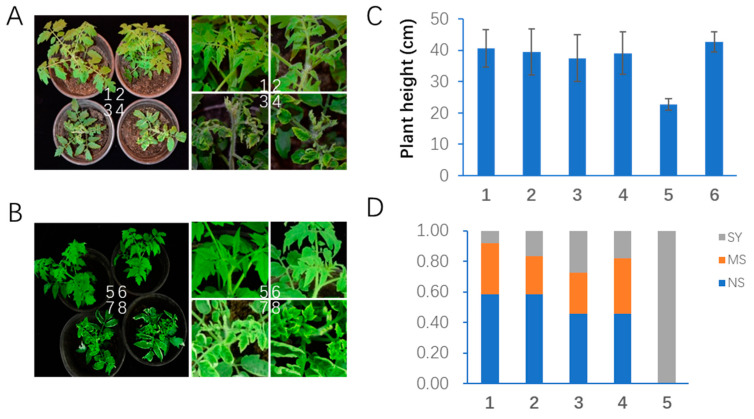
AMIN14 transgenic plants confer better resistance to mixed TYLCLV infection than AMIE14 transgenic plants. (**A**) Overview (left) and close view (right) of disease symptoms in AMIN14 transgenic (1 and 2 from line 61-1) and wild-type (3 and 4) plants. (**B**) Overview (left) and close view (right) of disease symptoms in AMIE14 transgenic (5 and 6 from line 55-2) and wild-type (7 and 8) plants. (**C**) Plant height comparison of infected and non-infected tomato plants: 1 and 2, TYLCV-infected AMIN14 line 61-1 and line 61-2; 3 and 4, TYLCV-infected AMIE14 line 55-1 and line 55-2; 5, TYLCV-infected wild-type tomato plants; 6, mock-inoculated wild-type tomato plants. (**D**) Comparison of disease severity between infected transgenic plants and wild-type plants. Numbers 1–5 represents genotypes as in C. SY, portion of symptomatic plants; MS, portion of mild symptomatic plants; NS, portion of non-symptomatic plants.

To assess which strategy conferred better resistance against TYLCLVs in tomato, pAMIE14 and pAMIN14 were transferred to tomato cultivar A57. After validating the T-DNA insertion, three homozygous lines (AN-1, AN-5 and AN-12) from the AMIN method and two homozygous lines (AE-1 and AE-6) from the AMIE method were tested against multiple TYLCLV infections. The wild-type tomato (WT) was taken as a control. The plants were infected with a mixture of six strains of TYLCLV, i.e., TYLCGUV, TYLCAxV, TYLCKAV, TYLCCSV, TYLCMLV, TYLCMAV and TYLCIDV, with Agrobacterium-mediated infiltration. The infected plants were accessed at 30 days post infection (dpi). Most of the control A57 plants showed severe disease symptoms, including leaf curling and yellowing, stunted growth, etc. (Figure 5A,B). In contrast, most of the transgenic lines expressing pAMIE14 and pAMIN14 remained symptomless or showed only mild symptoms (Figure 5A,B). Consistent with the phenotypic assessment, PCR analysis showed that TYLCGUV, TYLCAxV, TYLCCSV, TYLCMAV and TYLCMLV accumulated in higher levels in the control plants than in the transgenic plants (Figure S4). The plant heights of infected AMIE and AMIN transgenic plants were clearly higher than those of the infected A57 plants and were similar to the mock inoculated control, A57 (Figure 5C). The infected plants were further classified into non-symptomatic, severe symptomatic and mild symptomatic groups based on the severity of their symptoms. The A57 plants were all in the severe symptomatic group; in contrast, about 60% of the AMIN plants and 50% of the AMIE plants were asymptomatic (Figure 5D). These data suggested that both strategies conferred resistance to TYLCV infection, and the AMIN method performed slightly better.

### 2.6. The AMIN Method Confers Better Resistance against Mixed Infections in Plastic House Conditions

**Figure 6:**
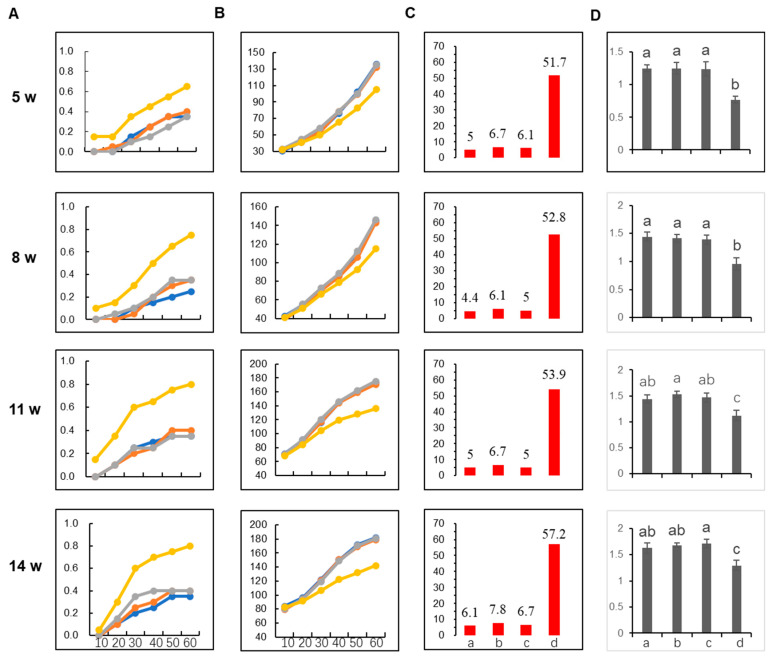
Disease-resistance phenotyping in plastic house conditions via whitefly infestation. (**A**) Progression of infection in tomato infected by whitefly. Y axis is the rate of plants showing disease phenotype. X axis shows the days after infection (DAI). Yellow line, Line A57; gray line, Line TY1; orange line, Line AE-6; blue line, Line AN-12 (same for B). (**B**) Plant height at different days after infection. Y axis is the plant height in centimeter. (**C**) Disease index of plants at 60 days after inoculation. Y axis is the accumulative disease index. X axis: a, Line AN-12; b, Line AE-6; c, TY1; d, control tomato line A57. (**D**) Stem diameters measured at 60 days after infection. Y axis is diameter in centimeters. X axis is the same as in C. Age of plants at the time of inoculation via whitefly is indicated to the left of each row. Letters a, b, c on top of each bar indicate significant differences determined by Student’s test (*p* < 0.05) while ab indicates no significant difference to a or b.

To further test the performance of the AMIE and AMIN transgenic plants in conditions close to real production, the above-tested AMIE line, AE-6, and AMIN line AN-12 were tested in a plastic house infected with whitefly infestation. The TY1 line was used as a positive control containing an *RDR3* that conferred resistance to TYLCV [27]. Four batches of plants were grown in a plastic house such that they were 5, 8, 11 and 14 weeks old when they were infected via whitefly infestation. Agrobacterium-mediated infection was also conducted for these different age groups as a positive control. Disease progression was observed in the plastic house and a disease index was calculated at 60 days after infection (DAI) (see Methods, Figure S5A). The results showed that the ratio of diseased tomato plants for all age groups infected by whiteflies gradually increased from 10 to 60 DAI. The disease in A57 progressed relatively fast and reached 65–80% by the end of the observation, while the disease in AE-6 and AN-12 progressed relatively slowly and reached 40% by the end of the observation, which is similar to the TY1 line (Figure 6A). Interestingly, the disease progressed much faster in positive control plants inoculated via Agrobacterium-mediated infiltration. The A57 plants in the 5- and 14-week-old groups reached 100% infection at 20 DAI (Figure S5B). Consistent with our observations of disease progression, the plant height of A57 increased slower than the other three genotypes, which were nearly the same (Figure 6B). At 60 DAI, the disease index for A57 ranged from 51.7 to 57.2; the AN-12 disease index ranged from 4.4 to 6.1; the AE-6 disease index ranged from 6.1 to 7.8; and the TY1 disease index ranged from 5 to 6.7 (Figure 6C). The results showed generally increased susceptibility as the tomato plants grew bigger (Figure 6C). According to the classification standard (Figure S5C), A57 showed high susceptibility in this assay. AE-6 and TY1 performed similarly, showing medium resistance, while AN-12 performed slightly better, showing high to medium resistance at different stages. The stem diameter of the infected plants was also examined at 60 DAI, and, similar to the height, the A57 stem diameter was smaller than that of the other three genotypes (Figure 6D). These results showed that the AMIE and AMIN methods conferred effective resistance in plastic house growth conditions, similar to the widely used TY1 resistance trait. AMIN performed slightly better than the AMIE method.

## 3. Discussion

TYLCD is a devastating disease that has threatened tomato production for decades. The causal agents are a complex of many TYLCV-like viruses belonging to the begomovirus genus. AMIR has been successfully applied to engineering viral resistance in plants [14,15]. However, recent report showed that single-AMIR-mediated viral resistance could be overcome by the virus by accumulating mutations at the target site [35]. The same report also suggested that the number of targeting sites is more relevant than the expression level in relation to the resistance level in AMIR or artificial TAS transgenic plants [35]. Thus, to obtain strong and durable resistance against TYLCVs, we designed 14 artificial miRNAs to target the conserved regions of viral protein-coding sequences to then engineer viral resistance in tomato (Figure 1 and Figure S1). These AMIRs were first clustered into dual AMIR clusters targeting each of the seven viral genes and then clustered into higher-order clusters targeting three, four and seven viral genes simultaneously (Figure 2), which are the largest AMIR clusters to date.

During miRNA processing, the 5′ and 3′ RNAs flanking miRNA/miRNA* are released via DCL1 cleavage with 3′ hydroxy and 5′ monophosphate ends, respectively. Their counterparts in animals have been shown to be degraded by exonuclease Xrn2 which interferes with clustered miRNA biogenesis in two ways; first, by causing RNA polymerase II release, thus inhibiting the transcription of downstream miRNAs, and second, by degrading flanking miRNA precursors [36]. However, this activity does not interfere with processing miRNA clusters with short spacers [36]. This is possibly caused by the coupled transcription of closely clustered miRNA processing and a synergistic effect in recruiting miRNA processing machinery for closely clustered miRNAs [37]. Thus, our dual clusters targeting the same viral genes were clustered with short spacers. However, a large number of closely clustered hairpins are technically challenging in DNA synthesis and cloning; thus, we inserted about 400–500 bp spacer sequences between the dual miRNA clusters when generating higher-order clusters. As we are not sure how the much longer spacers between the dual clusters would impact the function of the large miRNA cluster, we generated the large clusters in two ways: the AMIE way and the AMIN way (Figure 2 and Figure 3). In the AMIE method, all miRNAs are processed from one transcript, while, in the AMIN method, each dual cluster is released via splicing and processed individually, thus causing minimal interference with each other. The function of these AMIR constructs was validated with a transient assay and stable transgenic plants (Figure 3). The transgenic tomato lines were further tested for their resistance to TYLCLV infection. Interestingly, we found that the AMIN transgenic line performed slightly better than the AMIE line in both growth room conditions and plastic house conditions (Figure 5 and Figure 6), indicating that a long spacer between the miRNA dual clusters may impose a negative impact on miRNA biogenesis. Significantly, based on the disease index observed at the end of the observation, the AMIN transgenic line had a slightly better resistance level than the TY1 gene typically used in tomato breeding (Figure 6C). To test whether the over-expression of a large number of miRNAs might interfere with endogenous miRNA function, thus affecting tomato development and fruit quality in the absence of viral infections, we grew the AMIE, AMIN and A57 lines in a plastic house under regular care. Analyses of the physical parameters and fruit quality showed that they were similar to wild-type plants (Table S3). All these results indicated good potential in practice.

We also generated infectious clones for seven TYLCLV species that can initiate successful infections in *N. benthamiana* and tomato via Agrobacterium-mediated infiltration (Figure 4), and this will be very useful for future phenotyping related to TYLCLV resistance materials. To shorten the time until symptom expression, we chose to use a mixed infection with seven TYLCLV species. By comparing the disease progression and severity between the Agrobacterium- and whitefly-mediated infections, we found the former imposed much stronger infection pressure than the latter (Figure 6A and Figure S5B). Thus, in our case, the infection pressure might be much higher than in natural infections and, as such, further testing of the AMIN line’s performance in the field is worthwhile.

## 4. Materials and Methods

### 4.1. Construction of AMIR Expression Vectors

For artificial miRNA (AMIR) expression vector constructs, pK7LIC1.0 was used as a vector driven by 35S promoter and 35S terminator. All the AMIRs were generated through PCR (ligation-independent cloning) with an engineered SpeI site at the 5′ end and XbaI and XhoI sites at the 3′ end. The fusion PCR method has been followed to obtain a final PCR product where the 1st PCR-amplified product of the target gene was used as a template in the 2nd PCR and then the 2nd PCR-amplified product was used as a template in the 3rd PCR and vice versa to obtain the final product. The purified PCR product was digested with SpeI and the vector was digested with SpeI and SmaI. They were ligated together using T4 DNA ligase enzyme and subsequently transformed into DH5α. Each of the individual clusters with two designed artificial miRNAs (AMIRs) are connected together by a connector sequence. The individual miRNA clusters were joined together to obtain a Super miRNA cluster. The overexpression vector pK7LIC1.0 was digested using XbaI and XhoI enzymes with the removal of 9bp from the vector. However, the insert was digested with the enzymes SpeI and XhoI. T4 DNA ligase was used for ligation followed by transformation into DH5α. 

To produce microRNA sensory vectors, a duplex DNA structure was formed when two oligos containing an amiRCP binding site were annealed together, creating “TCGA” and “CTAG” 50 overhangs at both ends. The pMS4 vector was digested with XhoI and XbaI and ligated with the insert at 75 °C for 10 min with a gradual decrease in temperature of 0.1 °C/cycle to a temperature of 22 °C. The ligation product was transformed into DH5α competence cells.

### 4.2. Tobacco Transformation

Plants were grown aseptically in a Phytotray II box containing MS media until about two months of age. Plant leaves were cut into 0.5 cm × 0.5 cm pieces and placed in 10 mL of liquid basal medium (BM) in a Petri dish. A 1 ml volume of Agrobacterium culture containing AMIR constructs was added to the Petri dish and shaken for 45 min. 

Alternatively, we removed the leaf base and sliced the leaf blade into feather-like structures and placed 2~3 leaf feathers per plates. Excess liquid was blotted off from the leaf pieces by using a sterile filter paper and placed the leaf pieces on the surface of Petri plates containing basal medium plus cytokinin (BM+C, 15 pieces/plates). The plates were incubated in the dark at room temperature for 2–3 days. Then, the leaf pieces were transferred into new plates containing basal medium, cytokinin, plant selection antibiotics and carbenicillin (BM+C+AC, 10/plates). After 3–4 weeks, shoot initiation started from callus on some tissues. The shoots were cut off and the stem pushed into rooting media (RM) plus carbenicillin in a Phytotray II box (Sigma-Aldrich, St. Louis, MO, USA). After two weeks, roots developed and rooted plants were shifted to soil mixture in pots. 

### 4.3. Tomato Transformation

Agrobacterium-mediated tomato transformation was performed for pAMIN14 and pAMIE14. Tomato seeds were surface sterilized with 75% ethanol and 50% 84 disinfectant for 15 min. The sterilized seeds were transferred to Magenta boxes containing ½ MS media. The 10-day-old seedlings were shifted to damp sterile filter paper. Both the tip and petiole of cotyledon were removed and the remaining part was cut into two pieces. The cotyledon explants were placed on Petri plates containing KCMS medium covered with damp filter paper. The Petri plate was sealed with parafilm. The plates were cultured for 24 h in darkness. The explants were transferred to a large Petri dish containing 50 mL of 0.2 liquid MS medium. Almost 150 µL of Agrobacterium tumefaciens GV3101 harboring the pAMIN14 and pAMIE14 vectors of optical density (at 600 nm) was added in it and shaken gently for 4 minutes. The explants were blotted dry on sterile filter paper to remove any remaining bacteria. The explants were co-cultivated for 48 h in the dark by placing them upside-down on the original (KCMS) plates. The explants were transferred adaxial-side-up to 2Z medium. The plates were kept under white light with a 16 hour photoperiod. The plants developed callus on this medium and bud initiation started after 1 to 2 weeks. The explants that developed buds were shifted to 0.2 Z medium. On this medium, shoots were regenerated from callus within next 2–3 weeks. When shoots were 2 cm long, callus was removed and planted in conical flasks. They were kept under white fluorescent light for 16 h. Within 1–2 weeks, the regenerated plants developed roots. At 4–5 leaf stage, they were planted in soil.

### 4.4. Transient Assay by Agro-Infiltration

Agrobacterium cultures were incubated overnight in the shaker at 28 °C in 4 mL LB liquid media with 4 uL of 50 mg/mL spectinomycin and 50 mg/mL rifampicin each as selection antibiotics. Then, 100 μL of grown culture was shifted to induced media by adding 50 mL of LB media with the antibiotics used in the starter cultures, followed by adding 10 uL of 100 μM acetosyringone and 1 mL of 50 mM MES, and then put on the shaker for 16 h at 28 °C. After incubation, centrifugation of cultures was performed at 3000× *g* for 5 min. Pellets were formed which then dissolved in infiltration media (1 mL of 50 mM MES + 10 uL of 100 μM acetosyringone) to make the final O.D600 of 0.1. Agrobacterium suspension was infiltrated into 3-week-old *N. benthamiana* (WT) and *N. tabacum* transgenic plant leaves using a 1 mL syringe. After infiltration, plants were again kept in growth room conditions at 25 °C for three to four days. After 3–4 days, GFP expression was examined under a 365 nm UV lamp.

### 4.5. Preparation of TYLCLV Infectious Clones

One unit length of the TYLCLV genome was assembled using the gene synthesis approach as described before [34]. DNA oligos of 58 bp covering the entire length of the TYLCLV genome in both strands with a 29 bp overlapping region were ordered (Table S1) and treated with PNK to phosphorylate their 5′ ends. The treated oligos were ligated using *Taq* DNA ligase at high temperature and then amplified using terminal primers with *BamHI* sites and adapters for ligation-independent cloning (LIC) [38] (Table S1). The amplified TYLCLV genome was insert into the PHLic14 vector linearized with the enzyme *SmaI* using the LIC method to generate pTYLCLV-1. The cloned TYLCLV genome was verified by sequencing, released from the vector DNA by *BamHI* digestion and recovered through gel extraction. The recovered fragment was self-ligated using T4 DNA ligase. The self-ligated fragment was amplified with primers producing a fragment of 2.1 kb. The resulting fragment was treated with T4 DNA polymerase enzyme and ligated by LIC in pH7LIC14 to generate pTYLCLV-0.5. Finally, the one-unit TYLCLV genome from pTYLCLV-1 was cloned into the *BamHI* site in pTYLCLV-0.5 using restriction and ligation to produce pTYLCLV-1.5. The digested vector was subjected to phosphatase treatment to avoid self-ligation of the vector. Dephosphorylation of the vector was performed at 37 °C for half an hour and then enzyme inactivation was performed at 75 °C for 10 min.

### 4.6. TYLCLV Infection via Agrobacterium-Mediated Infiltration

TYLCLV infectious clone DNA was transformed into Agrobacterium using the heatshock method. Agrobacterium-mediated infiltration was essentially conducted as described before [39] with modifications. In brief, the OD600 of Agrobacterium harboring each TYLCLV clone was diluted to 1.0 and then mixed in equal volume before infiltration.

### 4.7. TYLCLV Infection via Whitefly Infestation

Virus-free whitefly was maintained in a cage feeding on virus-free tomato seedlings, which were replaced with healthy seedlings every two weeks. After passage of 3 to 5 generations, virus-free plants were replaced with TYLCLV-infected tomato seedlings. Whiteflies were allowed to feed on the infected plants for 3–5 days and then released into a plastic house with tomato plants to be infected. Pesticide was sprayed in the plastic house to kill whiteflies 10 days after they were released to protect plants from damage by the insects.

### 4.8. Disease Index and Infection Rate Calculation

The severity of tomato yellow leaf curl disease was divided into six levels, marked by the numbers 0, 1, 3, 5, 7 and 9 (Figure S4A) according to reported method [40]. The formula for calculating the disease index is as follows:

$$Disease\;Index=\frac{100\times \sum \;\left(\mathrm{No}.\;\mathrm{of}\;\mathrm{sick}\;\mathrm{leaves}\;\mathrm{at}\;\mathrm{all}\;\mathrm{levels}\times \;\mathrm{Representative}\;\mathrm{values}\;\mathrm{at}\;\mathrm{all}\;\mathrm{levels}\right)\;}{\mathrm{Total}\;\mathrm{number}\;\mathrm{of}\;\mathrm{leaves}\;\mathrm{in}\;\mathrm{the}\;\mathrm{survey}\times \mathrm{Highest}\;\mathrm{representative}\;\mathrm{value}}$$

$$Disease\;Rate=\frac{\mathrm{Number}\;\mathrm{of}\;\mathrm{diseased}\;\mathrm{plants}}{\mathrm{Total}\;\mathrm{number}\;\mathrm{of}\;\mathrm{plants}\;\mathrm{investigated}}\times 100$$

Four sets of diseased seedlings were studied to determine the disease index. Each set contained 20 plants for each genotype.

## Data Availability

Not applicable.

## Supplementary Materials

The following supporting information can be downloaded at: https://www.mdpi.com/article/10.3390/plants12112179/s1, Data S1: Genome sequences of TYLCLV for target site analysis; Data S2: AMIE and AMIN expression vector sequences; Data S3: miRNA sensor construct sequences; Table S1: Primers used for this study; Table S2: Information for cloned TYLCLV species; Table S3: Nutritional assessment of transgenic tomato fruit; Figure S1: Target site selection in TYLCLV- and satellite-virus-encoded genes; Figure S2: Mechanism for restoration of restriction sites in vector after ligation between vector and insert; Figure S3: Validation of amiRNA expression; Figure S4: Detection of TYLCLV DNA in systemic leaves of infected transgenic and wild type plants; Figure S5: Phenotyping viral resistance in plastic house condition.
