# Supplementary material for: Large Artificial microRNA Cluster Genes Confer Effective Resistance against Multiple Tomato Yellow Leaf Curl Viruses in Transgenic Tomato
Source: Plants (Basel). 2023 May 31;12(11):2179. doi: 10.3390/plants12112179 (PMC10255879; doi:10.3390/plants12112179)
Supplement: Supplementary file 1 [file plants-12-02179-s001.zip › plants-2357753-supplementary/plants-2357753-SM/Supplemental data S3 pMS4 sensor.docx]

**Supplemental data S3 microRNA pMS4 sensor constructs.** 35S promoter and GFP sequences are shaded in red and gray respectively. ER peptide signal sequences are bolded green. miRNAs binding site a are in bold red while miRNAs binding site b are in red italicized. The XbaI and XhoI restriction sites are in bold brown and underlined.

>pMS4-AMIRC1 (**AK0062 (1bp - 14675bp, direct) 14675bp**)

GGCCGCACTAGTGATATCCCGCGGCCATGGCGGCCGGGAGCATGCGACGTCGGGCCCAATTCGCCCTATA

GTGAGTCGTATTACAATTCACTGGCCGTCGTTTTACAACGTCGTGACTGGGAAAACCCTGGCGTTACCCA

ACTTAATCGCCTTGCAGCACATCCCCCTTTCGCCAGCTGGCGTAATAGCGAAGAGGCCCGCACCGATCGC

CCTTCCCAACAGTTGCGCAGCCTGAATGGCGAATGGAAATTGTAAACGTTAATGGGTTTCTGGAGTTTAA

TGAGCTAAGCACATACGTCAGAAACCATTATTGCGCGTTCAAAAGTCGCCTAAGGTCACTATCAGCTAGC

AAATATTTCTTGTCAAAAATGCTCCACTGACGTTCCATAAATTCCCCTCGGTATCCAATTAGAGTCTCAT

ATTCACTCTCAATCCAAATAATCTGCAATGGCAATTACCTTATCCGCAACTTCTTTACCTATTTCCGCCC

GGATCCGGGCAGGTTCTCCGGCCGCTTGGGTGGAGAGGCTATTCGGCTATGACTGGGCACAACAGACAAT

CGGCTGCTCTGATGCCGCCGTGTTCCGGCTGTCAGCGCAGGGGCGCCCGGTTCTTTTTGTCAAGACCGAC

CTGTCCGGTGCCCTGAATGAACTGCAGGACGAGGCAGCGCGGCTATCGTGGCTGGCCACGACGGGCGTTC

CTTGCGCAGCTGTGCTCGACGTTGTCACTGAAGCGGGAAGGGACTGGCTGCTATTGGGCGAAGTGCCGGG

GCAGGATCTCCTGTCATCTCACCTTGCTCCTGCCGAGAAAGTATCCATCATGGCTGATGCAATGCGGCGG

CTGCATACGCTTGATCCGGCTACCTGCCCATTCGACCACCAAGCGAAACATCGCATCGAGCGAGCACGTA

CTCGGATGGAAGCCGGTCTTGTCGATCAGGATGATCTGGACGAAGAGCATCAGGGGCTCGCGCCAGCCGA

ACTGTTCGCCAGGCTCAAGGCGCGCATGCCCGACGGCGAGGATCTCGTCGTGACCCATGGCGATGCCTGC

TTGCCGAATATCATGGTGGAAAATGGCCGCTTTTCTGGATTCATCGACTGTGGCCGGCTGGGTGTGGCGG

ACCGCTATCAGGACATAGCGTTGGCTACCCGTGATATTGCTGAAGAGCTTGGCGGCGAATGGGCTGACCG

CTTCCTCGTGCTTTACGGTATCGCCGCTCCCGATTCGCAGCGCATCGCCTTCTATCGCCTTCTTGACGAG

TTCTTCTGAGCGGGACTCTGGGGTTCGAAATGACCGACCAAGCGACGCCCAACCTGCCATCACGAGATTT

CGATTCCACCGCCGCCTTCTATGAAAGGTTGGGCTTCGGAATCGTTTTCCGGGACGCCGGCTGGATGATC

CTCCAGCGCGGGGATCTCATGCTGGAGTTCTTCGCCCACCCCGATCCAACACTTACGTTTGCAACGTCCA

AGAGCAAATAGACCACGAACGCCGGAAGGTTGCCGCAGCGTGTGGATTGCGTCTCAATTCTCTCTTGCAG

GAATGCAATGATGAATATGATACTGACTATGAAACTTTGAGGGAATACTGCCTAGCACCGTCACCTCATA

ACGTGCATCATGCATGCCCTGACAACATGGAACATCGCTATTTTTCTGAAGAATTATGCTCGTTGGAGGA

TGTCGCGGCAATTGCAGCTATTGCCAACATCGAACTACCCCTCACGCATGCATTCATCAATATTATTCAT

GCGGGGAAAGGCAAGATTAATCCAACTGGCAAATCATCCAGCGTGATTGGTAACTTCAGTTCCAGCGACT

TGATTCGTTTTGGTGCTACCCACGTTTTCAATAAGGACGAGATGGTGGAGTAAAGAAGGAGTGCGTCGAA

GCAGATCGTTCAAACATTTGGCAATAAAGTTTCTTAAGATTGAATCCTGTTGCCGGTCTTGCGATGATTA

TCATATAATTTCTGTTGAATTACGTTAAGCATGTAATAATTAACATGTAATGCATGACGTTATTTATGAG

ATGGGTTTTTATGATTAGAGTCCCGCAATTATACATTTAATACGCGATAGAAAACAAAATATAGCGCGCA

AACTAGGATAAATTATCGCGCGCGGTGTCATCTATGTTACTAGATCGAATTAATTCCAGGCGGTGAAGGG

CAATCAGCTGTTGCCCGTCTCACTGGTGAAAAGAAAAACCACCCCAGTACATTAAAAACGTCCGCAATGT

GTTATTAAGTTGTCTAAGCGTCAATTTGTTTACACCACAATATATCCTGCCACCAGCCAGCCAACAGCTC

CCCGACCGGCAGCTCGGCACAAAATCACCACTCGATACAGGCAGCCCATCAGTCCGGGACGGCGTCAGCG

GGAGAGCCGTTGTAAGGCGGCAGACTTTGCTCATGTTACCGATGCTATTCGGAAGAACGGCAACTAAGCT

GCCGGGTTTGAAACACGGATGATCTCGCGGAGGGTAGCATGTTGATTGTAACGATGACAGAGCGTTGCTG

CCTGTGATCAAATATCATCTCCCTCGCAGAGATCCGAATTATCAGCCTTCTTATTCATTTCTCGCTTAAC

CGTGACAGGCTGTCGATCTTGAGAACTATGCCGACATAATAGGAAATCGCTGGATAAAGCCGCTGAGGAA

GCTGAGTGGCGCTATTTCTTTAGAAGTGAACGTTGACGATGTCGACGGATCTTTTCCGCTGCATAACCCT

GCTTCGGGGTCATTATAGCGATTTTTTCGGTATATCCATCCTTTTTCGCACGATATACAGGATTTTGCCA

AAGGGTTCGTGTAGACTTTCCTTGGTGTATCCAACGGCGTCAGCCGGGCAGGATAGGTGAAGTAGGCCCA

CCCGCGAGCGGGTGTTCCTTCTTCACTGTCCCTTATTCGCACCTGGCGGTGCTCAACGGGAATCCTGCTC

TGCGAGGCTGGCCGGCTACCGCCGGCGTAACAGATGAGGGCAAGCGGATGGCTGATGAAACCAAGCCAAC

CAGGGGTGATGCTGCCAACTTACTGATTTAGTGTATGATGGTGTTTTTGAGGTGCTCCAGTGGCTTCTGT

TTCTATCAGCTGTCCCTCCTGTTCAGCTACTGACGGGGTGGTGCGTAACGGCAAAAGCACCGCCGGACAT

CAGCGCTATCTCTGCTCTCACTGCCGTAAAACATGGCAACTGCAGTTCACTTACACCGCTTCTCAACCCG

GTACGCACCAGAAAATCATTGATATGGCCATGAATGGCGTTGGATGCCGGGCAACAGCCCGCATTATGGG

CGTTGGCCTCAACACGATTTTACGTCACTTAAAAAACTCAGGCCGCAGTCGGTAACCTCGCGCATACAGC

CGGGCAGTGACGTCATCGTCTGCGCGGAAATGGACGAACAGTGGGGCTATGTCGGGGCTAAATCGCGCCA

GCGCTGGCTGTTTTACGCGTATGACAGTCTCCGGAAGACGGTTGTTGCGCACGTATTCGGTGAACGCACT

ATGGCGACGCTGGGGCGTCTTATGAGCCTGCTGTCACCCTTTGACGTGGTGATATGGATGACGGATGGCT

GGCCGCTGTATGAATCCCGCCTGAAGGGAAAGCTGCACGTAATCAGCAAGCGATATACGCAGCGAATTGA

GCGGCATAACCTGAATCTGAGGCAGCACCTGGCACGGCTGGGACGGAAGTCGCTGTCGTTCTCAAAATCG

GTGGAGCTGCATGACAAAGTCATCGGGCATTATCTGAACATAAAACACTATCAATAAGTTGGAGTCATTA

CCCAACCAGGAAGGGCAGCCCACCTATCAAGGTGTACTGCCTTCCAGACGAACGAAGAGCGATTGAGGAA

AAGGCGGCGGCGGCCGGCATGAGCCTGTCGGCCTACCTGCTGGCCGTCGGCCAGGGCTACAAAATCACGG

GCGTCGTGGACTATGAGCACGTCCGCGAGCTGGCCCGCATCAATGGCGACCTGGGCCGCCTGGGCGGCCT

GCTGAAACTCTGGCTCACCGACGACCCGCGCACGGCGCGGTTCGGTGATGCCACGATCCTCGCCCTGCTG

GCGAAGATCGAAGAGAAGCAGGACGAGCTTGGCAAGGTCATGATGGGCGTGGTCCGCCCGAGGGCAGAGC

CATGACTTTTTTAGCCGCTAAAACGGCCGGGGGGTGCGCGTGATTGCCAAGCACGTCCCCATGCGCTCCA

TCAAGAAGAGCGACTTCGCGGAGCTGGTATTCGTGCAGGGCAAGATTCGGAATACCAAGTACGAGAAGGA

CGGCCAGACGGTCTACGGGACCGACTTCATTGCCGATAAGGTGGATTATCTGGACACCAAGGCACCAGGC

GGGTCAAATCAGGAATAAGGGCACATTGCCCCGGCGTGAGTCGGGGCAATCCCGCAAGGAGGGTGAATGA

ATCGGACGTTTGACCGGAAGGCATACAGGCAAGAACTGATCGACGCGGGGTTTTCCGCCGAGGATGCCGA

AACCATCGCAAGCCGCACCGTCATGCGTGCGCCCCGCGAAACCTTCCAGTCCGTCGGCTCGATGGTCCAG

CAAGCTACGGCCAAGATCGAGCGCGACAGCGTGCAACTGGCTCCCCCTGCCCTGCCCGCGCCATCGGCCG

CCGTGGAGCGTTCGCGTCGTCTCGAACAGGAGGCGGCAGGTTTGGCGAAGTCGATGACCATCGACACGCG

AGGAACTATGACGACCAAGAAGCGAAAAACCGCCGGCGAGGACCTGGCAAAACAGGTCAGCGAGGCCAAG

CAGGCCGCGTTGCTGAAACACACGAAGCAGCAGATCAAGGAAATGCAGCTTTCCTTGTTCGATATTGCGC

CGTGGCCGGACACGATGCGAGCGATGCCAAACGACACGGCCCGCTCTGCCCTGTTCACCACGCGCAACAA

GAAAATCCCGCGCGAGGCGCTGCAAAACAAGGTCATTTTCCACGTCAACAAGGACGTGAAGATCACCTAC

ACCGGCGTCGAGCTGCGGGCCGACGATGACGAACTGGTGTGGCAGCAGGTGTTGGAGTACGCGAAGCGCA

CCCCTATCGGCGAGCCGATCACCTTCACGTTCTACGAGCTTTGCCAGGACCTGGGCTGGTCGATCAATGG

CCGGTATTACACGAAGGCCGAGGAATGCCTGTCGCGCCTACAGGCGACGGCGATGGGCTTCACGTCCGAC

CGCGTTGGGCACCTGGAATCGGTGTCGCTGCTGCACCGCTTCCGCGTCCTGGACCGTGGCAAGAAAACGT

CCCGTTGCCAGGTCCTGATCGACGAGGAAATCGTCGTGCTGTTTGCTGGCGACCACTACACGAAATTCAT

ATGGGAGAAGTACCGCAAGCTGTCGCCGACGGCCCGACGGATGTTCGACTATTTCAGCTCGCACCGGGAG

CCGTACCCGCTCAAGCTGGAAACCTTCCGCCTCATGTGCGGATCGGATTCCACCCGCGTGAAGAAGTGGC

GCGAGCAGGTCGGCGAAGCCTGCGAAGAGTTGCGAGGCAGCGGCCTGGTGGAACACGCCTGGGTCAATGA

TGACCTGGTGCATTGCAAACGCTAGGGCCTTGTGGGGTCAGTTCCGGCTGGGGGTTCAGCAGCCAGCGCT

TTACTGGCATTTCAGGAACAAGCGGGCACTGCTCGACGCACTTGCTTCGCTCAGTATCGCTCGGGACGCA

CGGCGCGCTCTACGAACTGCCGATAAACAGAGGATTAAAATTGACAATTGTGATTAAGGCTCAGATTCGA

CGGCTTGGAGCGGCCGACGTGCAGGATTTCCGCGAGATCCGATTGTCGGCCCTGAAGAAAGCTCCAGAGA

TGTTCGGGTCCGTTTACGAGCACGAGGAGAAAAAGCCCATGGAGGCGTTCGCTGAACGGTTGCGAGATGC

CGTGGCATTCGGCGCCTACATCGACGGCGAGATCATTGGGCTGTCGGTCTTCAAACAGGAGGACGGCCCC

AAGGACGCTCACAAGGCGCATCTGTCCGGCGTTTTCGTGGAGCCCGAACAGCGAGGCCGAGGGGTCGCCG

GTATGCTGCTGCGGGCGTTGCCGGCGGGTTTATTGCTCGTGATGATCGTCCGACAGATTCCAACGGGAAT

CTGGTGGATGCGCATCTTCATCCTCGGCGCACTTAATATTTCGCTATTCTGGAGCTTGTTGTTTATTTCG

GTCTACCGCCTGCCGGGCGGGGTCGCGGCGACGGTAGGCGCTGTGCAGCCGCTGATGGTCGTGTTCATCT

CTGCCGCTCTGCTAGGTAGCCCGATACGATTGATGGCGGTCCTGGGGGCTATTTGCGGAACTGCGGGCGT

GGCGCTGTTGGTGTTGACACCAAACGCAGCGCTAGATCCTGTCGGCGTCGCAGCGGGCCTGGCGGGGGCG

GTTTCCATGGCGTTCGGAACCGTGCTGACCCGCAAGTGGCAACCTCCCGTGCCTCTGCTCACCTTTACCG

CCTGGCAACTGGCGGCCGGAGGACTTCTGCTCGTTCCAGTAGCTTTAGTGTTTGATCCGCCAATCCCGAT

GCCTACAGGAACCAATGTTCTCGGCCTGGCGTGGCTCGGCCTGATCGGAGCGGGTTTAACCTACTTCCTT

TGGTTCCGGGGGATCTCGCGACTCGAACCTACAGTTGTTTCCTTACTGGGCTTTCTCAGCCGGGATGGCG

CTAAGAAGCTATTGCCGCCGATCTTCATATGCGGTGTGAAATACCGCACAGATGCGTAAGGAGAAAATAC

CGCATCAGGCGCTCTTCCGCTTCCTCGCTCACTGACTCGCTGCGCTCGGTCGTTCGGCTGCGGCGAGCGG

TATCAGCTCACTCAAAGGCGGTAATACGGTTATCCACAGAATCAGGGGATAACGCAGGAAAGAACATGTG

AGCAAAAGGCCAGCAAAAGGCCAGGAACCGTAAAAAGGCCGCGTTGCTGGCGTTTTTCCATAGGCTCCGC

CCCCCTGACGAGCATCACAAAAATCGACGCTCAAGTCAGAGGTGGCGAAACCCGACAGGACTATAAAGAT

ACCAGGCGTTTCCCCCTGGAAGCTCCCTCGTGCGCTCTCCTGTTCCGACCCTGCCGCTTACCGGATACCT

GTCCGCCTTTCTCCCTTCGGGAAGCGTGGCGCTTTCTCAATGCTCACGCTGTAGGTATCTCAGTTCGGTG

TAGGTCGTTCGCTCCAAGCTGGGCTGTGTGCACGAACCCCCCGTTCAGCCCGACCGCTGCGCCTTATCCG

GTAACTATCGTCTTGAGTCCAACCCGGTAAGACACGACTTATCGCCACTGGCAGCAGCCACTGGTAACAG

GATTAGCAGAGCGAGGTATGTAGGCGGTGCTACAGAGTTCTTGAAGTGGTGGCCTAACTACGGCTACACT

AGAAGGACAGTATTTGGTATCTGCGCTCTGCTGAAGCCAGTTACCTTCGGAAAAAGAGTTGGTAGCTCTT

GATCCGGCAAACAAACCACCGCTGGTAGCGGTGGTTTTTTTGTTTGCAAGCAGCAGATTACGCGCAGAAA

AAAAGGATATCAAGAAGATCCTTTGATCTTTTCTACGGGGTCTGACGCTCAGTGGAACGAAAACTCACGT

TAAGGGATTTTGGTCATGAGATTATCAAAAAGGATCTTCACCTAGATCCTTTTAAATTAAAAATGAAGTT

TTAAATCAATCTAAAGTATATATGAGTAAACTTGGTCTGACAGTTACCAATGCTTAATCAGTGAGGCACC

TATCTCAGCGATCTGTCTATTTCGTTCATCCATAGTTGCCTGACTCCCCGTCGTGTAGATAACTACGATA

CGGGAGGGCTTACCATCTGGCCCCAGTGCTGCAATGATACCGCGAGACCCACGCTCACCGGCTCCAGATT

TATCAGCAATAAACCAGCCAGCCGGAAGGGCCGAGCGCAGAAGTGGTCCTGCAACTTTATCCGCCTCCAT

CCAGTCTATTAAACAAGTGGCAGCAACGGATTCGCAAACCTGTCACGCCTTTTGTGCCAAAAGCCGCGCC

AGGTTTGCGATCCGCTGTGCCAGGCGTTAGGCGTCATATGAAGATTTCGGTGATCCCTGAGCAGGTGGCG

GAAACATTGGATGCTGAGAACCATTTCATTGTTCGTGAAGTGTTCGATGTGCACCTATCCGACCAAGGCT

TTGAACTATCTACCAGAAGTGTGAGCCCCTACCGGAAGGATTACATCTCGGATGATGACTCTGATGAAGA

CTCTGCTTGCTATGGCGCATTCATCGACCAAGAGCTTGTCGGGAAGATTGAACTCAACTCAACATGGAAC

GATCTAGCCTCTATCGAACACATTGTTGTGTCGCACACGCACCGAGGCAAAGGAGTCGCGCACAGTCTCA

TCGAATTTGCGAAAAAGTGGGCACTAAGCAGACAGCTCCTTGGCATACGATTAGAGACACAAACGAACAA

TGTACCTGCCTGCAATTTGTACGCAAAATGTGGCTTTACTCTCGGCGGCATTGACCTGTTCACGTATAAA

ACTAGACCTCAAGTCTCGAACGAAACAGCGATGTACTGGTACTGGTTCTCGGGAGCACAGGATGACGCCT

AACAATTCATTCAAGCCGACACCGCTTCGCGGCGCGGCTTAATTCAGGAGTTAAACATCATGAGGGAAGC

GGTGATCGCCGAAGTATCGACTCAACTATCAGAGGTAGTTGGCGTCATCGAGCGCCATCTCGAACCGACG

TTGCTGGCCGTACATTTGTACGGCTCCGCAGTGGATGGCGGCCTGAAGCCACACAGTGATATTGATTTGC

TGGTTACGGTGACCGTAAGGCTTGATGAAACAACGCGGCGAGCTTTGATCAACGACCTTTTGGAAACTTC

GGCTTCCCCTGGAGAGAGCGAGATTCTCCGCGCTGTAGAAGTCACCATTGTTGTGCACGACGACATCATT

CCGTGGCGTTATCCAGCTAAGCGCGAACTGCAATTTGGAGAATGGCAGCGCAATGACATTCTTGCAGGTA

TCTTCGAGCCAGCCACGATCGACATTGATCTGGCTATCTTGCTGACAAAAGCAAGAGAACATAGCGTTGC

CTTGGTAGGTCCAGCGGCGGAGGAACTCTTTGATCCGGTTCCTGAACAGGATCTATTTGAGGCGCTAAAT

GAAACCTTAACGCTATGGAACTCGCCGCCCGACTGGGCTGGCGATGAGCGAAATGTAGTGCTTACGTTGT

CCCGCATTTGGTACAGCGCAGTAACCGGCAAAATCGCGCCGAAGGATGTCGCTGCCGACTGGGCAATGGA

GCGCCTGCCGGCCCAGTATCAGCCCGTCATACTTGAAGCTAGGCAGGCTTATCTTGGACAAGAAGATCGC

TTGGCCTCGCGCGCAGATCAGTTGGAAGAATTTGTTCACTACGTGAAAGGCGAGATCACCAAGGTAGTCG

GCAAATAATGTCTAACAATTCGTTCAAGCCGACGCCGCTTCGCGGCGCGGCTTAACTCAAGCGTTAGAGA

GCTGGGGAAGACTATGCGCGATCTGTTGAAGGTGGTTCTAAGCCTCGTACTTGCGATGGCATCGGGGCAG

GCACTTGCTGACCTGCCAATTGTTTTAGTGGATGAAGCTCGTCTTCCCTATGACTACTCCCCATCCAACT

ACGACATTTCTCCAAGCAACTACGACAACTCCATAAGCAATTACGACAATAGTCCATCAAATTACGACAA

CTCTGAGAGCAACTACGATAATAGTTCATCCAATTACGACAATAGTCGCAACGGAAATCGTAGGCTTATA

TATAGCGCAAATGGGTCTCGCACTTTCGCCGGCTACTACGTCATTGCCAACAATGGGACAACGAACTTCT

TTTCCACATCTGGCAAAAGGATGTTCTACACCCCAAAAGGGGGGCGCGGCGTCTATGGCGGCAAAGATGG

GAGCTTCTGCGGGGCATTGGTCGTCATAAATGGCCAATTTTCGCTTGCCCTGACAGATAACGGCCTGAAG

ATCATGTATCTAAGCAACTAGCCTGCTCTCTAATAAAATGTTAGGAGCTTGGCTGCCATTTTTGGGGTGA

GGCCGTTCGCGGCCGAGGGGCGCAGCCCCTGGGGGGATGGGAGGCCCGCGTTAGCGGGCCGGGAGGGTTC

GAGAAGGGGGGGCACCCCCCTTCGGCGTGCGCGGTCACGCGCCAGGGCGCAGCCCTGGTTAAAAACAAGG

TTTATAAATATTGGTTTAAAAGCAGGTTAAAAGACAGGTTAGCGGTGGCCGAAAAACGGGCGGAAACCCT

TGCAAATGCTGGATTTTCTGCCTGTGGACAGCCCCTCAAATGTCAATAGGTGCGCCCCTCATCTGTCAGC

ACTCTGCCCCTCAAGTGTCAAGGATCGCGCCCCTCATCTGTCAGTAGTCGCGCCCCTCAAGTGTCAATAC

CGCAGGGCACTTATCCCCAGGCTTGTCCACATCATCTGTGGGAAACTCGCGTAAAATCAGGCGTTTTCGC

CGATTTGCGAGGCTGGCCAGCTCCACGTCGCCGGCCGAAATCGAGCCTGCCCCTCATCTGTCAACGCCGC

GCCGGGTGAGTCGGCCCCTCAAGTGTCAACGTCCGCCCCTCATCTGTCAGTGAGGGCCAAGTTTTCCGCG

AGGTATCCACAACGCCGGCGGCCGGCCGCGGTGTCTCGCACACGGCTTCGACGGCGTTTCTGGCGCGTTT

GCAGGGCCATAGACGGCCGCCAGCCCAGCGGCGAGGGCAACCAGCCCGGTGAGCGTCGGAAAGGGTCGAC

ATCTTGCTGCGTTCGGATATTTTCGTGGAGTTCCCGCCACAGACCCGGATTGAAGGCGAGATCCAGCAAC

TCGCGCCAGATCATCCTGTGACGGAACTTTGGCGCGTGATGACTGGCCAGGACGTCGGCCGAAAGAGCGA

CAAGCAGATCACGATTTTCGACAGCGTCGGATTTGCGATCGAGGATTTTTCGGCGCTGCGCTACGTCCGC

GACCGCGTTGAGGGATCAAGCCACAGCAGCCCACTCGACCTTCTAGCCGACCCAGACGAGCCAAGGGATC

TTTTTGGAATGCTGCTCCGTCGTCAGGCTTTCCGACGTTTGGGTGGTTGAACAGAAGTCATTATCGTACG

GAATGCCAGCACTCCCGAGGGGAACCCTGTGGTTGGCATGCACATACAAATGGACGAACGGATAAACCTT

TTCACGCCCTTTTAAATATCCGTTATTCTAATAAACGCTCTTTTCTCTTAGGTTTACCCGCCAATATATC

CTGTCAAACACTGATAGTTTAAACTGAAGGCGGGAAACGACAATCTGATCATGAGCGGAGAATTAAGGGA

GTCACGTTATGACCCCCGCCGATGACGCGGGACAAGCCGTTTTACGTTTGGAACTGACAGAACCGCAACG

ATTGAAGGAGCCACTCAGCCCCAATACGCAAACCGCCTCTCCCCGCGCGTTGGCCGATTCATTAATGCAG

CTGGCACGACAGGTTTCCCGACTGGAAAGCGGGCAGTGAGCGCAACGCAATTAATGTGAGTTAGCTCACT

CATTAGGCACCCCAGGCTTTACACTTTATGCTTCCGGCTCGTATGTTGTGTGGAATTGTGAGCGGATAAC

AATTTCACACAGGAAACAGCTATGACCATGATTACGCCAAGCTATTTAGGTGACACTATAGAATACTCAA

GCTATGCATCCAACGCGTTGGGAGCTCTCCCATATCGACCTGCAGGCGGCCGCTCGACGAATTAATTCCA

ATCCCACAAAAATCTGAGCTTAACAGCACAGTTGCTCCTCTCAGAGCAGAATCGGGTATTCAACACCCTC

ATATCAACTACTACGTTGTGTATAACGGTCCACATGCCGGTATATACGATGACTGGGGTTGTACAAAGGC

GGCAACAAACGGCGTTCCCGGAGTTGCACACAAGAAATTTGCCACTATTACAGAGGCAAGAGCAGCAGCT

GACGCGTACACAACAAGTCAGCAAACAGACAGGTTGAACTTCATCCCCAAAGGAGAAGCTCAACTCAAGC

CCAAGAGCTTTGCTAAGGCCCTAACAAGCCCACCAAAGCAAAAAGCCCACTGGCTCACGCTAGGAACCAA

AAGGCCCAGCAGTGATCCAGCCCCAAAAGAGATCTCCTTTGCCCCGGAGATTACAATGGACGATTTCCTC

TATCTTTACGATCTAGGAAGGAAGTTCGAAGGTGAAGGTGACGACACTATGTTCACCACTGATAATGAGA

AGGTTAGCCTCTTCAATTTCAGAAAGAATGCTGACCCACAGATGGTTAGAGAGGCCTACGCAGCAGGTCT

CATCAAGACGATCTACCCGAGTAACAATCTCCAGGAGATCAAATACCTTCCCAAGAAGGTTAAAGATGCA

GTCAAAAGATTCAGGACTAATTGCATCAAGAACACAGAGAAAGACATATTTCTCAAGATCAGAAGTACTA

TTCCAGTATGGACGATTCAAGGCTTGCTTCATAAACCAAGGCAAGTAATAGAGATTGGAGTCTCTAAAAA

GGTAGTTCCTACTGAATCTAAGGCCATGCATGGAGTCTAAGATTCAAATCGAGGATCTAACAGAACTCGC

CGTGAAGACTGGCGAACAGTTCATACAGAGTCTTTTACGACTCAATGACAAGAAGAAAATCTTCGTCAAC

ATGGTGGAGCACGACACTCTGGTCTACTCCAAAAATGTCAAAGATACAGTCTCAGAAGACCAAAGGGCTA

TTGAGACTTTTCAACAAAGGATAATTTCGGGAAACCTCCTCGGATTCCATTGCCCAGCTATCTGTCACTT

CATCGAAAGGACAGTAGAAAAGGAAGGTGGCTCCTACAAATGCCATCATTGCGATAAAGGAAAGGCTATC

ATTCAAGATCTCTCTGCCGACAGTGGTCCCAAAGATGGACCCCCACCCACGAGGAGCATCGTGGAAAAAG

AAGACGTTCCAACCACGTCTTCAAAGCAAGTGGATTGATGTGACATCTCCACTGACGTAAGGGATGACGC

ACAATCCCACTATCCTTCGCAAGACCCTTCCTCTATATAAGGAAGTTCATTTCATTTGGAGAGGACACGC

TCGACTATTTTTACAACAATTACC**ATGAAGACTAATCTTTTTCTCTTTCTCATCTTTTCACTTCTCCTAT**

**CATTATCCTCGGCCGAATTCCTCGAG**TCAC**GCCCAAAGGGACTGGCAAAGCA***ACAATGTGGGCCAGGTCT*

*CTAA***TCTAGA**AGTAAAGGAGAAGAACTTTTCACTGGAGTTGTCCCAATTCTTGTTGAATTAGATGGTGAT

GTTAATGGGTACAAATTTTCTGTCAGTGGAGAGGGTGAAGGTGATGCAACATACGGAAAACTTACCCTTA

AATTTATTTGCACTACTGGAAAACTACCTGTTCCATGGCCAACACTTGTCACTACTTTCTCTTATGGTGT

TCAATGCTTTTCAAGATACCCAGATCATATGAAGCGGCACGACTTCTTCAAGAGCGCCATGCCTGAGGGA

TACGTGCAGGAGAGGACCATCTTCTTCAAGGACGACGGGAACTACAAGACACGTGCTGAAGTCAAGTTTG

AGGGAGACACCCTCGTCAACAGGATCGAGCTTAAGGGAATCGATTTCAAGGAGGACGGAAACATCCTCGG

CCACAAGTTGGAATACAACTACAACTCCCACAACGTATACATCATGGCCGACAAGCAAAAGAACGGCATC

AAAGCCAACTTCAAGACCCGCCACAACATCGAAGACGGCGGCGTGCAACTCGCTGATCATTATCAACAAA

ATACTCCAATTGGCGATGGCCCTGTCCTTTTACCAGACAACCATTACCTGTCCACACAATCTGCCCTTTC

GAAAGATCCCAACGAAAAGAGAGACCACATGGTCCTTCTTGAGTTTGTAACAGCTGCTGGGATTACACAT

GGCATGGATGAACTATACAAACATGATGAGCTTTGAACTAGAGTCCTGCTTTAATGAGATATGCGAGACG

CCTATGATCGCATGATATTTGCTTTCAATTCTGTTGTGCACGTTGTAAAAAACCTGAGCATGTGTAGCTC

AGATCCTTACCGCCGGTTTCGGTTCATTCTAATGAATATATCACCCGTTACTATCGTATTTTTATGAATA

ATATTCTCCGTTCAATTTACTGATTGTACCCTACTACTTATATGTACAATATTAAAATGAAAACAATATA

TTGTGCTGAATAGGTTTATAGCGACATCTATGATAGAGCGCCACAATAACAAACAATTGCGTTTTATTAT

TACAAATCCAATTTTAAAAAAAGCGGCAGAACCGGTCAAACCTAAAAGACTGATTACATAAATCTTATTC

AAATTTCAAAAGGCCCCAGGGGCTAGTATCTACGACACACCGAGCGGCGAACTAATAACGTTCACTGAAG

GGAACTCCGGTTCCCCGCCGGCGCGCATGGGTGAGATTCCTTGAAGTTGAGTATTGGCCGTCCGCTCTAC

CGAAAGTTACGGGCACCATTCAACCCGGTCCAGCACGGCGGCCGGGTAACCGACTTGCTGCCCCGAGAAT

TATGCAGCATTTTTTTGGTGTATGTGGGCCCCAAATGAAGTGCAGGTCAAACCTTGACAGTGACGACAAA

TCGTTGGGCGGGTCCAGGGCGAATTTTGCGACAACATGTCGAGGCTCAGCAGGACCTGCAGGCATGCAAG

CTAGCTTACTAGTGATGCATATTCTATAGTGTCACCTAAATCTGC

>pMS4-AMIRC2 (**AK0063 (1bp - 14675bp, direct) 14675bp**)

GGCCGCACTAGTGATATCCCGCGGCCATGGCGGCCGGGAGCATGCGACGTCGGGCCCAATTCGCCCTATA

GTGAGTCGTATTACAATTCACTGGCCGTCGTTTTACAACGTCGTGACTGGGAAAACCCTGGCGTTACCCA

ACTTAATCGCCTTGCAGCACATCCCCCTTTCGCCAGCTGGCGTAATAGCGAAGAGGCCCGCACCGATCGC

CCTTCCCAACAGTTGCGCAGCCTGAATGGCGAATGGAAATTGTAAACGTTAATGGGTTTCTGGAGTTTAA

TGAGCTAAGCACATACGTCAGAAACCATTATTGCGCGTTCAAAAGTCGCCTAAGGTCACTATCAGCTAGC

AAATATTTCTTGTCAAAAATGCTCCACTGACGTTCCATAAATTCCCCTCGGTATCCAATTAGAGTCTCAT

ATTCACTCTCAATCCAAATAATCTGCAATGGCAATTACCTTATCCGCAACTTCTTTACCTATTTCCGCCC

GGATCCGGGCAGGTTCTCCGGCCGCTTGGGTGGAGAGGCTATTCGGCTATGACTGGGCACAACAGACAAT

CGGCTGCTCTGATGCCGCCGTGTTCCGGCTGTCAGCGCAGGGGCGCCCGGTTCTTTTTGTCAAGACCGAC

CTGTCCGGTGCCCTGAATGAACTGCAGGACGAGGCAGCGCGGCTATCGTGGCTGGCCACGACGGGCGTTC

CTTGCGCAGCTGTGCTCGACGTTGTCACTGAAGCGGGAAGGGACTGGCTGCTATTGGGCGAAGTGCCGGG

GCAGGATCTCCTGTCATCTCACCTTGCTCCTGCCGAGAAAGTATCCATCATGGCTGATGCAATGCGGCGG

CTGCATACGCTTGATCCGGCTACCTGCCCATTCGACCACCAAGCGAAACATCGCATCGAGCGAGCACGTA

CTCGGATGGAAGCCGGTCTTGTCGATCAGGATGATCTGGACGAAGAGCATCAGGGGCTCGCGCCAGCCGA

ACTGTTCGCCAGGCTCAAGGCGCGCATGCCCGACGGCGAGGATCTCGTCGTGACCCATGGCGATGCCTGC

TTGCCGAATATCATGGTGGAAAATGGCCGCTTTTCTGGATTCATCGACTGTGGCCGGCTGGGTGTGGCGG

ACCGCTATCAGGACATAGCGTTGGCTACCCGTGATATTGCTGAAGAGCTTGGCGGCGAATGGGCTGACCG

CTTCCTCGTGCTTTACGGTATCGCCGCTCCCGATTCGCAGCGCATCGCCTTCTATCGCCTTCTTGACGAG

TTCTTCTGAGCGGGACTCTGGGGTTCGAAATGACCGACCAAGCGACGCCCAACCTGCCATCACGAGATTT

CGATTCCACCGCCGCCTTCTATGAAAGGTTGGGCTTCGGAATCGTTTTCCGGGACGCCGGCTGGATGATC

CTCCAGCGCGGGGATCTCATGCTGGAGTTCTTCGCCCACCCCGATCCAACACTTACGTTTGCAACGTCCA

AGAGCAAATAGACCACGAACGCCGGAAGGTTGCCGCAGCGTGTGGATTGCGTCTCAATTCTCTCTTGCAG

GAATGCAATGATGAATATGATACTGACTATGAAACTTTGAGGGAATACTGCCTAGCACCGTCACCTCATA

ACGTGCATCATGCATGCCCTGACAACATGGAACATCGCTATTTTTCTGAAGAATTATGCTCGTTGGAGGA

TGTCGCGGCAATTGCAGCTATTGCCAACATCGAACTACCCCTCACGCATGCATTCATCAATATTATTCAT

GCGGGGAAAGGCAAGATTAATCCAACTGGCAAATCATCCAGCGTGATTGGTAACTTCAGTTCCAGCGACT

TGATTCGTTTTGGTGCTACCCACGTTTTCAATAAGGACGAGATGGTGGAGTAAAGAAGGAGTGCGTCGAA

GCAGATCGTTCAAACATTTGGCAATAAAGTTTCTTAAGATTGAATCCTGTTGCCGGTCTTGCGATGATTA

TCATATAATTTCTGTTGAATTACGTTAAGCATGTAATAATTAACATGTAATGCATGACGTTATTTATGAG

ATGGGTTTTTATGATTAGAGTCCCGCAATTATACATTTAATACGCGATAGAAAACAAAATATAGCGCGCA

AACTAGGATAAATTATCGCGCGCGGTGTCATCTATGTTACTAGATCGAATTAATTCCAGGCGGTGAAGGG

CAATCAGCTGTTGCCCGTCTCACTGGTGAAAAGAAAAACCACCCCAGTACATTAAAAACGTCCGCAATGT

GTTATTAAGTTGTCTAAGCGTCAATTTGTTTACACCACAATATATCCTGCCACCAGCCAGCCAACAGCTC

CCCGACCGGCAGCTCGGCACAAAATCACCACTCGATACAGGCAGCCCATCAGTCCGGGACGGCGTCAGCG

GGAGAGCCGTTGTAAGGCGGCAGACTTTGCTCATGTTACCGATGCTATTCGGAAGAACGGCAACTAAGCT

GCCGGGTTTGAAACACGGATGATCTCGCGGAGGGTAGCATGTTGATTGTAACGATGACAGAGCGTTGCTG

CCTGTGATCAAATATCATCTCCCTCGCAGAGATCCGAATTATCAGCCTTCTTATTCATTTCTCGCTTAAC

CGTGACAGGCTGTCGATCTTGAGAACTATGCCGACATAATAGGAAATCGCTGGATAAAGCCGCTGAGGAA

GCTGAGTGGCGCTATTTCTTTAGAAGTGAACGTTGACGATGTCGACGGATCTTTTCCGCTGCATAACCCT

GCTTCGGGGTCATTATAGCGATTTTTTCGGTATATCCATCCTTTTTCGCACGATATACAGGATTTTGCCA

AAGGGTTCGTGTAGACTTTCCTTGGTGTATCCAACGGCGTCAGCCGGGCAGGATAGGTGAAGTAGGCCCA

CCCGCGAGCGGGTGTTCCTTCTTCACTGTCCCTTATTCGCACCTGGCGGTGCTCAACGGGAATCCTGCTC

TGCGAGGCTGGCCGGCTACCGCCGGCGTAACAGATGAGGGCAAGCGGATGGCTGATGAAACCAAGCCAAC

CAGGGGTGATGCTGCCAACTTACTGATTTAGTGTATGATGGTGTTTTTGAGGTGCTCCAGTGGCTTCTGT

TTCTATCAGCTGTCCCTCCTGTTCAGCTACTGACGGGGTGGTGCGTAACGGCAAAAGCACCGCCGGACAT

CAGCGCTATCTCTGCTCTCACTGCCGTAAAACATGGCAACTGCAGTTCACTTACACCGCTTCTCAACCCG

GTACGCACCAGAAAATCATTGATATGGCCATGAATGGCGTTGGATGCCGGGCAACAGCCCGCATTATGGG

CGTTGGCCTCAACACGATTTTACGTCACTTAAAAAACTCAGGCCGCAGTCGGTAACCTCGCGCATACAGC

CGGGCAGTGACGTCATCGTCTGCGCGGAAATGGACGAACAGTGGGGCTATGTCGGGGCTAAATCGCGCCA

GCGCTGGCTGTTTTACGCGTATGACAGTCTCCGGAAGACGGTTGTTGCGCACGTATTCGGTGAACGCACT

ATGGCGACGCTGGGGCGTCTTATGAGCCTGCTGTCACCCTTTGACGTGGTGATATGGATGACGGATGGCT

GGCCGCTGTATGAATCCCGCCTGAAGGGAAAGCTGCACGTAATCAGCAAGCGATATACGCAGCGAATTGA

GCGGCATAACCTGAATCTGAGGCAGCACCTGGCACGGCTGGGACGGAAGTCGCTGTCGTTCTCAAAATCG

GTGGAGCTGCATGACAAAGTCATCGGGCATTATCTGAACATAAAACACTATCAATAAGTTGGAGTCATTA

CCCAACCAGGAAGGGCAGCCCACCTATCAAGGTGTACTGCCTTCCAGACGAACGAAGAGCGATTGAGGAA

AAGGCGGCGGCGGCCGGCATGAGCCTGTCGGCCTACCTGCTGGCCGTCGGCCAGGGCTACAAAATCACGG

GCGTCGTGGACTATGAGCACGTCCGCGAGCTGGCCCGCATCAATGGCGACCTGGGCCGCCTGGGCGGCCT

GCTGAAACTCTGGCTCACCGACGACCCGCGCACGGCGCGGTTCGGTGATGCCACGATCCTCGCCCTGCTG

GCGAAGATCGAAGAGAAGCAGGACGAGCTTGGCAAGGTCATGATGGGCGTGGTCCGCCCGAGGGCAGAGC

CATGACTTTTTTAGCCGCTAAAACGGCCGGGGGGTGCGCGTGATTGCCAAGCACGTCCCCATGCGCTCCA

TCAAGAAGAGCGACTTCGCGGAGCTGGTATTCGTGCAGGGCAAGATTCGGAATACCAAGTACGAGAAGGA

CGGCCAGACGGTCTACGGGACCGACTTCATTGCCGATAAGGTGGATTATCTGGACACCAAGGCACCAGGC

GGGTCAAATCAGGAATAAGGGCACATTGCCCCGGCGTGAGTCGGGGCAATCCCGCAAGGAGGGTGAATGA

ATCGGACGTTTGACCGGAAGGCATACAGGCAAGAACTGATCGACGCGGGGTTTTCCGCCGAGGATGCCGA

AACCATCGCAAGCCGCACCGTCATGCGTGCGCCCCGCGAAACCTTCCAGTCCGTCGGCTCGATGGTCCAG

CAAGCTACGGCCAAGATCGAGCGCGACAGCGTGCAACTGGCTCCCCCTGCCCTGCCCGCGCCATCGGCCG

CCGTGGAGCGTTCGCGTCGTCTCGAACAGGAGGCGGCAGGTTTGGCGAAGTCGATGACCATCGACACGCG

AGGAACTATGACGACCAAGAAGCGAAAAACCGCCGGCGAGGACCTGGCAAAACAGGTCAGCGAGGCCAAG

CAGGCCGCGTTGCTGAAACACACGAAGCAGCAGATCAAGGAAATGCAGCTTTCCTTGTTCGATATTGCGC

CGTGGCCGGACACGATGCGAGCGATGCCAAACGACACGGCCCGCTCTGCCCTGTTCACCACGCGCAACAA

GAAAATCCCGCGCGAGGCGCTGCAAAACAAGGTCATTTTCCACGTCAACAAGGACGTGAAGATCACCTAC

ACCGGCGTCGAGCTGCGGGCCGACGATGACGAACTGGTGTGGCAGCAGGTGTTGGAGTACGCGAAGCGCA

CCCCTATCGGCGAGCCGATCACCTTCACGTTCTACGAGCTTTGCCAGGACCTGGGCTGGTCGATCAATGG

CCGGTATTACACGAAGGCCGAGGAATGCCTGTCGCGCCTACAGGCGACGGCGATGGGCTTCACGTCCGAC

CGCGTTGGGCACCTGGAATCGGTGTCGCTGCTGCACCGCTTCCGCGTCCTGGACCGTGGCAAGAAAACGT

CCCGTTGCCAGGTCCTGATCGACGAGGAAATCGTCGTGCTGTTTGCTGGCGACCACTACACGAAATTCAT

ATGGGAGAAGTACCGCAAGCTGTCGCCGACGGCCCGACGGATGTTCGACTATTTCAGCTCGCACCGGGAG

CCGTACCCGCTCAAGCTGGAAACCTTCCGCCTCATGTGCGGATCGGATTCCACCCGCGTGAAGAAGTGGC

GCGAGCAGGTCGGCGAAGCCTGCGAAGAGTTGCGAGGCAGCGGCCTGGTGGAACACGCCTGGGTCAATGA

TGACCTGGTGCATTGCAAACGCTAGGGCCTTGTGGGGTCAGTTCCGGCTGGGGGTTCAGCAGCCAGCGCT

TTACTGGCATTTCAGGAACAAGCGGGCACTGCTCGACGCACTTGCTTCGCTCAGTATCGCTCGGGACGCA

CGGCGCGCTCTACGAACTGCCGATAAACAGAGGATTAAAATTGACAATTGTGATTAAGGCTCAGATTCGA

CGGCTTGGAGCGGCCGACGTGCAGGATTTCCGCGAGATCCGATTGTCGGCCCTGAAGAAAGCTCCAGAGA

TGTTCGGGTCCGTTTACGAGCACGAGGAGAAAAAGCCCATGGAGGCGTTCGCTGAACGGTTGCGAGATGC

CGTGGCATTCGGCGCCTACATCGACGGCGAGATCATTGGGCTGTCGGTCTTCAAACAGGAGGACGGCCCC

AAGGACGCTCACAAGGCGCATCTGTCCGGCGTTTTCGTGGAGCCCGAACAGCGAGGCCGAGGGGTCGCCG

GTATGCTGCTGCGGGCGTTGCCGGCGGGTTTATTGCTCGTGATGATCGTCCGACAGATTCCAACGGGAAT

CTGGTGGATGCGCATCTTCATCCTCGGCGCACTTAATATTTCGCTATTCTGGAGCTTGTTGTTTATTTCG

GTCTACCGCCTGCCGGGCGGGGTCGCGGCGACGGTAGGCGCTGTGCAGCCGCTGATGGTCGTGTTCATCT

CTGCCGCTCTGCTAGGTAGCCCGATACGATTGATGGCGGTCCTGGGGGCTATTTGCGGAACTGCGGGCGT

GGCGCTGTTGGTGTTGACACCAAACGCAGCGCTAGATCCTGTCGGCGTCGCAGCGGGCCTGGCGGGGGCG

GTTTCCATGGCGTTCGGAACCGTGCTGACCCGCAAGTGGCAACCTCCCGTGCCTCTGCTCACCTTTACCG

CCTGGCAACTGGCGGCCGGAGGACTTCTGCTCGTTCCAGTAGCTTTAGTGTTTGATCCGCCAATCCCGAT

GCCTACAGGAACCAATGTTCTCGGCCTGGCGTGGCTCGGCCTGATCGGAGCGGGTTTAACCTACTTCCTT

TGGTTCCGGGGGATCTCGCGACTCGAACCTACAGTTGTTTCCTTACTGGGCTTTCTCAGCCGGGATGGCG

CTAAGAAGCTATTGCCGCCGATCTTCATATGCGGTGTGAAATACCGCACAGATGCGTAAGGAGAAAATAC

CGCATCAGGCGCTCTTCCGCTTCCTCGCTCACTGACTCGCTGCGCTCGGTCGTTCGGCTGCGGCGAGCGG

TATCAGCTCACTCAAAGGCGGTAATACGGTTATCCACAGAATCAGGGGATAACGCAGGAAAGAACATGTG

AGCAAAAGGCCAGCAAAAGGCCAGGAACCGTAAAAAGGCCGCGTTGCTGGCGTTTTTCCATAGGCTCCGC

CCCCCTGACGAGCATCACAAAAATCGACGCTCAAGTCAGAGGTGGCGAAACCCGACAGGACTATAAAGAT

ACCAGGCGTTTCCCCCTGGAAGCTCCCTCGTGCGCTCTCCTGTTCCGACCCTGCCGCTTACCGGATACCT

GTCCGCCTTTCTCCCTTCGGGAAGCGTGGCGCTTTCTCAATGCTCACGCTGTAGGTATCTCAGTTCGGTG

TAGGTCGTTCGCTCCAAGCTGGGCTGTGTGCACGAACCCCCCGTTCAGCCCGACCGCTGCGCCTTATCCG

GTAACTATCGTCTTGAGTCCAACCCGGTAAGACACGACTTATCGCCACTGGCAGCAGCCACTGGTAACAG

GATTAGCAGAGCGAGGTATGTAGGCGGTGCTACAGAGTTCTTGAAGTGGTGGCCTAACTACGGCTACACT

AGAAGGACAGTATTTGGTATCTGCGCTCTGCTGAAGCCAGTTACCTTCGGAAAAAGAGTTGGTAGCTCTT

GATCCGGCAAACAAACCACCGCTGGTAGCGGTGGTTTTTTTGTTTGCAAGCAGCAGATTACGCGCAGAAA

AAAAGGATATCAAGAAGATCCTTTGATCTTTTCTACGGGGTCTGACGCTCAGTGGAACGAAAACTCACGT

TAAGGGATTTTGGTCATGAGATTATCAAAAAGGATCTTCACCTAGATCCTTTTAAATTAAAAATGAAGTT

TTAAATCAATCTAAAGTATATATGAGTAAACTTGGTCTGACAGTTACCAATGCTTAATCAGTGAGGCACC

TATCTCAGCGATCTGTCTATTTCGTTCATCCATAGTTGCCTGACTCCCCGTCGTGTAGATAACTACGATA

CGGGAGGGCTTACCATCTGGCCCCAGTGCTGCAATGATACCGCGAGACCCACGCTCACCGGCTCCAGATT

TATCAGCAATAAACCAGCCAGCCGGAAGGGCCGAGCGCAGAAGTGGTCCTGCAACTTTATCCGCCTCCAT

CCAGTCTATTAAACAAGTGGCAGCAACGGATTCGCAAACCTGTCACGCCTTTTGTGCCAAAAGCCGCGCC

AGGTTTGCGATCCGCTGTGCCAGGCGTTAGGCGTCATATGAAGATTTCGGTGATCCCTGAGCAGGTGGCG

GAAACATTGGATGCTGAGAACCATTTCATTGTTCGTGAAGTGTTCGATGTGCACCTATCCGACCAAGGCT

TTGAACTATCTACCAGAAGTGTGAGCCCCTACCGGAAGGATTACATCTCGGATGATGACTCTGATGAAGA

CTCTGCTTGCTATGGCGCATTCATCGACCAAGAGCTTGTCGGGAAGATTGAACTCAACTCAACATGGAAC

GATCTAGCCTCTATCGAACACATTGTTGTGTCGCACACGCACCGAGGCAAAGGAGTCGCGCACAGTCTCA

TCGAATTTGCGAAAAAGTGGGCACTAAGCAGACAGCTCCTTGGCATACGATTAGAGACACAAACGAACAA

TGTACCTGCCTGCAATTTGTACGCAAAATGTGGCTTTACTCTCGGCGGCATTGACCTGTTCACGTATAAA

ACTAGACCTCAAGTCTCGAACGAAACAGCGATGTACTGGTACTGGTTCTCGGGAGCACAGGATGACGCCT

AACAATTCATTCAAGCCGACACCGCTTCGCGGCGCGGCTTAATTCAGGAGTTAAACATCATGAGGGAAGC

GGTGATCGCCGAAGTATCGACTCAACTATCAGAGGTAGTTGGCGTCATCGAGCGCCATCTCGAACCGACG

TTGCTGGCCGTACATTTGTACGGCTCCGCAGTGGATGGCGGCCTGAAGCCACACAGTGATATTGATTTGC

TGGTTACGGTGACCGTAAGGCTTGATGAAACAACGCGGCGAGCTTTGATCAACGACCTTTTGGAAACTTC

GGCTTCCCCTGGAGAGAGCGAGATTCTCCGCGCTGTAGAAGTCACCATTGTTGTGCACGACGACATCATT

CCGTGGCGTTATCCAGCTAAGCGCGAACTGCAATTTGGAGAATGGCAGCGCAATGACATTCTTGCAGGTA

TCTTCGAGCCAGCCACGATCGACATTGATCTGGCTATCTTGCTGACAAAAGCAAGAGAACATAGCGTTGC

CTTGGTAGGTCCAGCGGCGGAGGAACTCTTTGATCCGGTTCCTGAACAGGATCTATTTGAGGCGCTAAAT

GAAACCTTAACGCTATGGAACTCGCCGCCCGACTGGGCTGGCGATGAGCGAAATGTAGTGCTTACGTTGT

CCCGCATTTGGTACAGCGCAGTAACCGGCAAAATCGCGCCGAAGGATGTCGCTGCCGACTGGGCAATGGA

GCGCCTGCCGGCCCAGTATCAGCCCGTCATACTTGAAGCTAGGCAGGCTTATCTTGGACAAGAAGATCGC

TTGGCCTCGCGCGCAGATCAGTTGGAAGAATTTGTTCACTACGTGAAAGGCGAGATCACCAAGGTAGTCG

GCAAATAATGTCTAACAATTCGTTCAAGCCGACGCCGCTTCGCGGCGCGGCTTAACTCAAGCGTTAGAGA

GCTGGGGAAGACTATGCGCGATCTGTTGAAGGTGGTTCTAAGCCTCGTACTTGCGATGGCATCGGGGCAG

GCACTTGCTGACCTGCCAATTGTTTTAGTGGATGAAGCTCGTCTTCCCTATGACTACTCCCCATCCAACT

ACGACATTTCTCCAAGCAACTACGACAACTCCATAAGCAATTACGACAATAGTCCATCAAATTACGACAA

CTCTGAGAGCAACTACGATAATAGTTCATCCAATTACGACAATAGTCGCAACGGAAATCGTAGGCTTATA

TATAGCGCAAATGGGTCTCGCACTTTCGCCGGCTACTACGTCATTGCCAACAATGGGACAACGAACTTCT

TTTCCACATCTGGCAAAAGGATGTTCTACACCCCAAAAGGGGGGCGCGGCGTCTATGGCGGCAAAGATGG

GAGCTTCTGCGGGGCATTGGTCGTCATAAATGGCCAATTTTCGCTTGCCCTGACAGATAACGGCCTGAAG

ATCATGTATCTAAGCAACTAGCCTGCTCTCTAATAAAATGTTAGGAGCTTGGCTGCCATTTTTGGGGTGA

GGCCGTTCGCGGCCGAGGGGCGCAGCCCCTGGGGGGATGGGAGGCCCGCGTTAGCGGGCCGGGAGGGTTC

GAGAAGGGGGGGCACCCCCCTTCGGCGTGCGCGGTCACGCGCCAGGGCGCAGCCCTGGTTAAAAACAAGG

TTTATAAATATTGGTTTAAAAGCAGGTTAAAAGACAGGTTAGCGGTGGCCGAAAAACGGGCGGAAACCCT

TGCAAATGCTGGATTTTCTGCCTGTGGACAGCCCCTCAAATGTCAATAGGTGCGCCCCTCATCTGTCAGC

ACTCTGCCCCTCAAGTGTCAAGGATCGCGCCCCTCATCTGTCAGTAGTCGCGCCCCTCAAGTGTCAATAC

CGCAGGGCACTTATCCCCAGGCTTGTCCACATCATCTGTGGGAAACTCGCGTAAAATCAGGCGTTTTCGC

CGATTTGCGAGGCTGGCCAGCTCCACGTCGCCGGCCGAAATCGAGCCTGCCCCTCATCTGTCAACGCCGC

GCCGGGTGAGTCGGCCCCTCAAGTGTCAACGTCCGCCCCTCATCTGTCAGTGAGGGCCAAGTTTTCCGCG

AGGTATCCACAACGCCGGCGGCCGGCCGCGGTGTCTCGCACACGGCTTCGACGGCGTTTCTGGCGCGTTT

GCAGGGCCATAGACGGCCGCCAGCCCAGCGGCGAGGGCAACCAGCCCGGTGAGCGTCGGAAAGGGTCGAC

ATCTTGCTGCGTTCGGATATTTTCGTGGAGTTCCCGCCACAGACCCGGATTGAAGGCGAGATCCAGCAAC

TCGCGCCAGATCATCCTGTGACGGAACTTTGGCGCGTGATGACTGGCCAGGACGTCGGCCGAAAGAGCGA

CAAGCAGATCACGATTTTCGACAGCGTCGGATTTGCGATCGAGGATTTTTCGGCGCTGCGCTACGTCCGC

GACCGCGTTGAGGGATCAAGCCACAGCAGCCCACTCGACCTTCTAGCCGACCCAGACGAGCCAAGGGATC

TTTTTGGAATGCTGCTCCGTCGTCAGGCTTTCCGACGTTTGGGTGGTTGAACAGAAGTCATTATCGTACG

GAATGCCAGCACTCCCGAGGGGAACCCTGTGGTTGGCATGCACATACAAATGGACGAACGGATAAACCTT

TTCACGCCCTTTTAAATATCCGTTATTCTAATAAACGCTCTTTTCTCTTAGGTTTACCCGCCAATATATC

CTGTCAAACACTGATAGTTTAAACTGAAGGCGGGAAACGACAATCTGATCATGAGCGGAGAATTAAGGGA

GTCACGTTATGACCCCCGCCGATGACGCGGGACAAGCCGTTTTACGTTTGGAACTGACAGAACCGCAACG

ATTGAAGGAGCCACTCAGCCCCAATACGCAAACCGCCTCTCCCCGCGCGTTGGCCGATTCATTAATGCAG

CTGGCACGACAGGTTTCCCGACTGGAAAGCGGGCAGTGAGCGCAACGCAATTAATGTGAGTTAGCTCACT

CATTAGGCACCCCAGGCTTTACACTTTATGCTTCCGGCTCGTATGTTGTGTGGAATTGTGAGCGGATAAC

AATTTCACACAGGAAACAGCTATGACCATGATTACGCCAAGCTATTTAGGTGACACTATAGAATACTCAA

GCTATGCATCCAACGCGTTGGGAGCTCTCCCATATCGACCTGCAGGCGGCCGCTCGACGAATTAATTCCA

ATCCCACAAAAATCTGAGCTTAACAGCACAGTTGCTCCTCTCAGAGCAGAATCGGGTATTCAACACCCTC

ATATCAACTACTACGTTGTGTATAACGGTCCACATGCCGGTATATACGATGACTGGGGTTGTACAAAGGC

GGCAACAAACGGCGTTCCCGGAGTTGCACACAAGAAATTTGCCACTATTACAGAGGCAAGAGCAGCAGCT

GACGCGTACACAACAAGTCAGCAAACAGACAGGTTGAACTTCATCCCCAAAGGAGAAGCTCAACTCAAGC

CCAAGAGCTTTGCTAAGGCCCTAACAAGCCCACCAAAGCAAAAAGCCCACTGGCTCACGCTAGGAACCAA

AAGGCCCAGCAGTGATCCAGCCCCAAAAGAGATCTCCTTTGCCCCGGAGATTACAATGGACGATTTCCTC

TATCTTTACGATCTAGGAAGGAAGTTCGAAGGTGAAGGTGACGACACTATGTTCACCACTGATAATGAGA

AGGTTAGCCTCTTCAATTTCAGAAAGAATGCTGACCCACAGATGGTTAGAGAGGCCTACGCAGCAGGTCT

CATCAAGACGATCTACCCGAGTAACAATCTCCAGGAGATCAAATACCTTCCCAAGAAGGTTAAAGATGCA

GTCAAAAGATTCAGGACTAATTGCATCAAGAACACAGAGAAAGACATATTTCTCAAGATCAGAAGTACTA

TTCCAGTATGGACGATTCAAGGCTTGCTTCATAAACCAAGGCAAGTAATAGAGATTGGAGTCTCTAAAAA

GGTAGTTCCTACTGAATCTAAGGCCATGCATGGAGTCTAAGATTCAAATCGAGGATCTAACAGAACTCGC

CGTGAAGACTGGCGAACAGTTCATACAGAGTCTTTTACGACTCAATGACAAGAAGAAAATCTTCGTCAAC

ATGGTGGAGCACGACACTCTGGTCTACTCCAAAAATGTCAAAGATACAGTCTCAGAAGACCAAAGGGCTA

TTGAGACTTTTCAACAAAGGATAATTTCGGGAAACCTCCTCGGATTCCATTGCCCAGCTATCTGTCACTT

CATCGAAAGGACAGTAGAAAAGGAAGGTGGCTCCTACAAATGCCATCATTGCGATAAAGGAAAGGCTATC

ATTCAAGATCTCTCTGCCGACAGTGGTCCCAAAGATGGACCCCCACCCACGAGGAGCATCGTGGAAAAAG

AAGACGTTCCAACCACGTCTTCAAAGCAAGTGGATTGATGTGACATCTCCACTGACGTAAGGGATGACGC

ACAATCCCACTATCCTTCGCAAGACCCTTCCTCTATATAAGGAAGTTCATTTCATTTGGAGAGGACACGC

TCGACTATTTTTACAACAATTACC**ATGAAGACTAATCTTTTTCTCTTTCTCATCTTTTCACTTCTCCTAT**

**CATTATCCTCGGCCGAATTCCTCGAG**TCAC**CATGGATTCACACACAGGGGAA***TGGCGTCTTTATCTGGAA*

*GATA***TCTAGA**AGTAAAGGAGAAGAACTTTTCACTGGAGTTGTCCCAATTCTTGTTGAATTAGATGGTGAT

GTTAATGGGTACAAATTTTCTGTCAGTGGAGAGGGTGAAGGTGATGCAACATACGGAAAACTTACCCTTA

AATTTATTTGCACTACTGGAAAACTACCTGTTCCATGGCCAACACTTGTCACTACTTTCTCTTATGGTGT

TCAATGCTTTTCAAGATACCCAGATCATATGAAGCGGCACGACTTCTTCAAGAGCGCCATGCCTGAGGGA

TACGTGCAGGAGAGGACCATCTTCTTCAAGGACGACGGGAACTACAAGACACGTGCTGAAGTCAAGTTTG

AGGGAGACACCCTCGTCAACAGGATCGAGCTTAAGGGAATCGATTTCAAGGAGGACGGAAACATCCTCGG

CCACAAGTTGGAATACAACTACAACTCCCACAACGTATACATCATGGCCGACAAGCAAAAGAACGGCATC

AAAGCCAACTTCAAGACCCGCCACAACATCGAAGACGGCGGCGTGCAACTCGCTGATCATTATCAACAAA

ATACTCCAATTGGCGATGGCCCTGTCCTTTTACCAGACAACCATTACCTGTCCACACAATCTGCCCTTTC

GAAAGATCCCAACGAAAAGAGAGACCACATGGTCCTTCTTGAGTTTGTAACAGCTGCTGGGATTACACAT

GGCATGGATGAACTATACAAACATGATGAGCTTTGAACTAGAGTCCTGCTTTAATGAGATATGCGAGACG

CCTATGATCGCATGATATTTGCTTTCAATTCTGTTGTGCACGTTGTAAAAAACCTGAGCATGTGTAGCTC

AGATCCTTACCGCCGGTTTCGGTTCATTCTAATGAATATATCACCCGTTACTATCGTATTTTTATGAATA

ATATTCTCCGTTCAATTTACTGATTGTACCCTACTACTTATATGTACAATATTAAAATGAAAACAATATA

TTGTGCTGAATAGGTTTATAGCGACATCTATGATAGAGCGCCACAATAACAAACAATTGCGTTTTATTAT

TACAAATCCAATTTTAAAAAAAGCGGCAGAACCGGTCAAACCTAAAAGACTGATTACATAAATCTTATTC

AAATTTCAAAAGGCCCCAGGGGCTAGTATCTACGACACACCGAGCGGCGAACTAATAACGTTCACTGAAG

GGAACTCCGGTTCCCCGCCGGCGCGCATGGGTGAGATTCCTTGAAGTTGAGTATTGGCCGTCCGCTCTAC

CGAAAGTTACGGGCACCATTCAACCCGGTCCAGCACGGCGGCCGGGTAACCGACTTGCTGCCCCGAGAAT

TATGCAGCATTTTTTTGGTGTATGTGGGCCCCAAATGAAGTGCAGGTCAAACCTTGACAGTGACGACAAA

TCGTTGGGCGGGTCCAGGGCGAATTTTGCGACAACATGTCGAGGCTCAGCAGGACCTGCAGGCATGCAAG

CTAGCTTACTAGTGATGCATATTCTATAGTGTCACCTAAATCTGC

>pMS4-AMIRC3 (**AK0064 (1bp - 14675bp, direct) 14675bp**)

GGCCGCACTAGTGATATCCCGCGGCCATGGCGGCCGGGAGCATGCGACGTCGGGCCCAATTCGCCCTATA

GTGAGTCGTATTACAATTCACTGGCCGTCGTTTTACAACGTCGTGACTGGGAAAACCCTGGCGTTACCCA

ACTTAATCGCCTTGCAGCACATCCCCCTTTCGCCAGCTGGCGTAATAGCGAAGAGGCCCGCACCGATCGC

CCTTCCCAACAGTTGCGCAGCCTGAATGGCGAATGGAAATTGTAAACGTTAATGGGTTTCTGGAGTTTAA

TGAGCTAAGCACATACGTCAGAAACCATTATTGCGCGTTCAAAAGTCGCCTAAGGTCACTATCAGCTAGC

AAATATTTCTTGTCAAAAATGCTCCACTGACGTTCCATAAATTCCCCTCGGTATCCAATTAGAGTCTCAT

ATTCACTCTCAATCCAAATAATCTGCAATGGCAATTACCTTATCCGCAACTTCTTTACCTATTTCCGCCC

GGATCCGGGCAGGTTCTCCGGCCGCTTGGGTGGAGAGGCTATTCGGCTATGACTGGGCACAACAGACAAT

CGGCTGCTCTGATGCCGCCGTGTTCCGGCTGTCAGCGCAGGGGCGCCCGGTTCTTTTTGTCAAGACCGAC

CTGTCCGGTGCCCTGAATGAACTGCAGGACGAGGCAGCGCGGCTATCGTGGCTGGCCACGACGGGCGTTC

CTTGCGCAGCTGTGCTCGACGTTGTCACTGAAGCGGGAAGGGACTGGCTGCTATTGGGCGAAGTGCCGGG

GCAGGATCTCCTGTCATCTCACCTTGCTCCTGCCGAGAAAGTATCCATCATGGCTGATGCAATGCGGCGG

CTGCATACGCTTGATCCGGCTACCTGCCCATTCGACCACCAAGCGAAACATCGCATCGAGCGAGCACGTA

CTCGGATGGAAGCCGGTCTTGTCGATCAGGATGATCTGGACGAAGAGCATCAGGGGCTCGCGCCAGCCGA

ACTGTTCGCCAGGCTCAAGGCGCGCATGCCCGACGGCGAGGATCTCGTCGTGACCCATGGCGATGCCTGC

TTGCCGAATATCATGGTGGAAAATGGCCGCTTTTCTGGATTCATCGACTGTGGCCGGCTGGGTGTGGCGG

ACCGCTATCAGGACATAGCGTTGGCTACCCGTGATATTGCTGAAGAGCTTGGCGGCGAATGGGCTGACCG

CTTCCTCGTGCTTTACGGTATCGCCGCTCCCGATTCGCAGCGCATCGCCTTCTATCGCCTTCTTGACGAG

TTCTTCTGAGCGGGACTCTGGGGTTCGAAATGACCGACCAAGCGACGCCCAACCTGCCATCACGAGATTT

CGATTCCACCGCCGCCTTCTATGAAAGGTTGGGCTTCGGAATCGTTTTCCGGGACGCCGGCTGGATGATC

CTCCAGCGCGGGGATCTCATGCTGGAGTTCTTCGCCCACCCCGATCCAACACTTACGTTTGCAACGTCCA

AGAGCAAATAGACCACGAACGCCGGAAGGTTGCCGCAGCGTGTGGATTGCGTCTCAATTCTCTCTTGCAG

GAATGCAATGATGAATATGATACTGACTATGAAACTTTGAGGGAATACTGCCTAGCACCGTCACCTCATA

ACGTGCATCATGCATGCCCTGACAACATGGAACATCGCTATTTTTCTGAAGAATTATGCTCGTTGGAGGA

TGTCGCGGCAATTGCAGCTATTGCCAACATCGAACTACCCCTCACGCATGCATTCATCAATATTATTCAT

GCGGGGAAAGGCAAGATTAATCCAACTGGCAAATCATCCAGCGTGATTGGTAACTTCAGTTCCAGCGACT

TGATTCGTTTTGGTGCTACCCACGTTTTCAATAAGGACGAGATGGTGGAGTAAAGAAGGAGTGCGTCGAA

GCAGATCGTTCAAACATTTGGCAATAAAGTTTCTTAAGATTGAATCCTGTTGCCGGTCTTGCGATGATTA

TCATATAATTTCTGTTGAATTACGTTAAGCATGTAATAATTAACATGTAATGCATGACGTTATTTATGAG

ATGGGTTTTTATGATTAGAGTCCCGCAATTATACATTTAATACGCGATAGAAAACAAAATATAGCGCGCA

AACTAGGATAAATTATCGCGCGCGGTGTCATCTATGTTACTAGATCGAATTAATTCCAGGCGGTGAAGGG

CAATCAGCTGTTGCCCGTCTCACTGGTGAAAAGAAAAACCACCCCAGTACATTAAAAACGTCCGCAATGT

GTTATTAAGTTGTCTAAGCGTCAATTTGTTTACACCACAATATATCCTGCCACCAGCCAGCCAACAGCTC

CCCGACCGGCAGCTCGGCACAAAATCACCACTCGATACAGGCAGCCCATCAGTCCGGGACGGCGTCAGCG

GGAGAGCCGTTGTAAGGCGGCAGACTTTGCTCATGTTACCGATGCTATTCGGAAGAACGGCAACTAAGCT

GCCGGGTTTGAAACACGGATGATCTCGCGGAGGGTAGCATGTTGATTGTAACGATGACAGAGCGTTGCTG

CCTGTGATCAAATATCATCTCCCTCGCAGAGATCCGAATTATCAGCCTTCTTATTCATTTCTCGCTTAAC

CGTGACAGGCTGTCGATCTTGAGAACTATGCCGACATAATAGGAAATCGCTGGATAAAGCCGCTGAGGAA

GCTGAGTGGCGCTATTTCTTTAGAAGTGAACGTTGACGATGTCGACGGATCTTTTCCGCTGCATAACCCT

GCTTCGGGGTCATTATAGCGATTTTTTCGGTATATCCATCCTTTTTCGCACGATATACAGGATTTTGCCA

AAGGGTTCGTGTAGACTTTCCTTGGTGTATCCAACGGCGTCAGCCGGGCAGGATAGGTGAAGTAGGCCCA

CCCGCGAGCGGGTGTTCCTTCTTCACTGTCCCTTATTCGCACCTGGCGGTGCTCAACGGGAATCCTGCTC

TGCGAGGCTGGCCGGCTACCGCCGGCGTAACAGATGAGGGCAAGCGGATGGCTGATGAAACCAAGCCAAC

CAGGGGTGATGCTGCCAACTTACTGATTTAGTGTATGATGGTGTTTTTGAGGTGCTCCAGTGGCTTCTGT

TTCTATCAGCTGTCCCTCCTGTTCAGCTACTGACGGGGTGGTGCGTAACGGCAAAAGCACCGCCGGACAT

CAGCGCTATCTCTGCTCTCACTGCCGTAAAACATGGCAACTGCAGTTCACTTACACCGCTTCTCAACCCG

GTACGCACCAGAAAATCATTGATATGGCCATGAATGGCGTTGGATGCCGGGCAACAGCCCGCATTATGGG

CGTTGGCCTCAACACGATTTTACGTCACTTAAAAAACTCAGGCCGCAGTCGGTAACCTCGCGCATACAGC

CGGGCAGTGACGTCATCGTCTGCGCGGAAATGGACGAACAGTGGGGCTATGTCGGGGCTAAATCGCGCCA

GCGCTGGCTGTTTTACGCGTATGACAGTCTCCGGAAGACGGTTGTTGCGCACGTATTCGGTGAACGCACT

ATGGCGACGCTGGGGCGTCTTATGAGCCTGCTGTCACCCTTTGACGTGGTGATATGGATGACGGATGGCT

GGCCGCTGTATGAATCCCGCCTGAAGGGAAAGCTGCACGTAATCAGCAAGCGATATACGCAGCGAATTGA

GCGGCATAACCTGAATCTGAGGCAGCACCTGGCACGGCTGGGACGGAAGTCGCTGTCGTTCTCAAAATCG

GTGGAGCTGCATGACAAAGTCATCGGGCATTATCTGAACATAAAACACTATCAATAAGTTGGAGTCATTA

CCCAACCAGGAAGGGCAGCCCACCTATCAAGGTGTACTGCCTTCCAGACGAACGAAGAGCGATTGAGGAA

AAGGCGGCGGCGGCCGGCATGAGCCTGTCGGCCTACCTGCTGGCCGTCGGCCAGGGCTACAAAATCACGG

GCGTCGTGGACTATGAGCACGTCCGCGAGCTGGCCCGCATCAATGGCGACCTGGGCCGCCTGGGCGGCCT

GCTGAAACTCTGGCTCACCGACGACCCGCGCACGGCGCGGTTCGGTGATGCCACGATCCTCGCCCTGCTG

GCGAAGATCGAAGAGAAGCAGGACGAGCTTGGCAAGGTCATGATGGGCGTGGTCCGCCCGAGGGCAGAGC

CATGACTTTTTTAGCCGCTAAAACGGCCGGGGGGTGCGCGTGATTGCCAAGCACGTCCCCATGCGCTCCA

TCAAGAAGAGCGACTTCGCGGAGCTGGTATTCGTGCAGGGCAAGATTCGGAATACCAAGTACGAGAAGGA

CGGCCAGACGGTCTACGGGACCGACTTCATTGCCGATAAGGTGGATTATCTGGACACCAAGGCACCAGGC

GGGTCAAATCAGGAATAAGGGCACATTGCCCCGGCGTGAGTCGGGGCAATCCCGCAAGGAGGGTGAATGA

ATCGGACGTTTGACCGGAAGGCATACAGGCAAGAACTGATCGACGCGGGGTTTTCCGCCGAGGATGCCGA

AACCATCGCAAGCCGCACCGTCATGCGTGCGCCCCGCGAAACCTTCCAGTCCGTCGGCTCGATGGTCCAG

CAAGCTACGGCCAAGATCGAGCGCGACAGCGTGCAACTGGCTCCCCCTGCCCTGCCCGCGCCATCGGCCG

CCGTGGAGCGTTCGCGTCGTCTCGAACAGGAGGCGGCAGGTTTGGCGAAGTCGATGACCATCGACACGCG

AGGAACTATGACGACCAAGAAGCGAAAAACCGCCGGCGAGGACCTGGCAAAACAGGTCAGCGAGGCCAAG

CAGGCCGCGTTGCTGAAACACACGAAGCAGCAGATCAAGGAAATGCAGCTTTCCTTGTTCGATATTGCGC

CGTGGCCGGACACGATGCGAGCGATGCCAAACGACACGGCCCGCTCTGCCCTGTTCACCACGCGCAACAA

GAAAATCCCGCGCGAGGCGCTGCAAAACAAGGTCATTTTCCACGTCAACAAGGACGTGAAGATCACCTAC

ACCGGCGTCGAGCTGCGGGCCGACGATGACGAACTGGTGTGGCAGCAGGTGTTGGAGTACGCGAAGCGCA

CCCCTATCGGCGAGCCGATCACCTTCACGTTCTACGAGCTTTGCCAGGACCTGGGCTGGTCGATCAATGG

CCGGTATTACACGAAGGCCGAGGAATGCCTGTCGCGCCTACAGGCGACGGCGATGGGCTTCACGTCCGAC

CGCGTTGGGCACCTGGAATCGGTGTCGCTGCTGCACCGCTTCCGCGTCCTGGACCGTGGCAAGAAAACGT

CCCGTTGCCAGGTCCTGATCGACGAGGAAATCGTCGTGCTGTTTGCTGGCGACCACTACACGAAATTCAT

ATGGGAGAAGTACCGCAAGCTGTCGCCGACGGCCCGACGGATGTTCGACTATTTCAGCTCGCACCGGGAG

CCGTACCCGCTCAAGCTGGAAACCTTCCGCCTCATGTGCGGATCGGATTCCACCCGCGTGAAGAAGTGGC

GCGAGCAGGTCGGCGAAGCCTGCGAAGAGTTGCGAGGCAGCGGCCTGGTGGAACACGCCTGGGTCAATGA

TGACCTGGTGCATTGCAAACGCTAGGGCCTTGTGGGGTCAGTTCCGGCTGGGGGTTCAGCAGCCAGCGCT

TTACTGGCATTTCAGGAACAAGCGGGCACTGCTCGACGCACTTGCTTCGCTCAGTATCGCTCGGGACGCA

CGGCGCGCTCTACGAACTGCCGATAAACAGAGGATTAAAATTGACAATTGTGATTAAGGCTCAGATTCGA

CGGCTTGGAGCGGCCGACGTGCAGGATTTCCGCGAGATCCGATTGTCGGCCCTGAAGAAAGCTCCAGAGA

TGTTCGGGTCCGTTTACGAGCACGAGGAGAAAAAGCCCATGGAGGCGTTCGCTGAACGGTTGCGAGATGC

CGTGGCATTCGGCGCCTACATCGACGGCGAGATCATTGGGCTGTCGGTCTTCAAACAGGAGGACGGCCCC

AAGGACGCTCACAAGGCGCATCTGTCCGGCGTTTTCGTGGAGCCCGAACAGCGAGGCCGAGGGGTCGCCG

GTATGCTGCTGCGGGCGTTGCCGGCGGGTTTATTGCTCGTGATGATCGTCCGACAGATTCCAACGGGAAT

CTGGTGGATGCGCATCTTCATCCTCGGCGCACTTAATATTTCGCTATTCTGGAGCTTGTTGTTTATTTCG

GTCTACCGCCTGCCGGGCGGGGTCGCGGCGACGGTAGGCGCTGTGCAGCCGCTGATGGTCGTGTTCATCT

CTGCCGCTCTGCTAGGTAGCCCGATACGATTGATGGCGGTCCTGGGGGCTATTTGCGGAACTGCGGGCGT

GGCGCTGTTGGTGTTGACACCAAACGCAGCGCTAGATCCTGTCGGCGTCGCAGCGGGCCTGGCGGGGGCG

GTTTCCATGGCGTTCGGAACCGTGCTGACCCGCAAGTGGCAACCTCCCGTGCCTCTGCTCACCTTTACCG

CCTGGCAACTGGCGGCCGGAGGACTTCTGCTCGTTCCAGTAGCTTTAGTGTTTGATCCGCCAATCCCGAT

GCCTACAGGAACCAATGTTCTCGGCCTGGCGTGGCTCGGCCTGATCGGAGCGGGTTTAACCTACTTCCTT

TGGTTCCGGGGGATCTCGCGACTCGAACCTACAGTTGTTTCCTTACTGGGCTTTCTCAGCCGGGATGGCG

CTAAGAAGCTATTGCCGCCGATCTTCATATGCGGTGTGAAATACCGCACAGATGCGTAAGGAGAAAATAC

CGCATCAGGCGCTCTTCCGCTTCCTCGCTCACTGACTCGCTGCGCTCGGTCGTTCGGCTGCGGCGAGCGG

TATCAGCTCACTCAAAGGCGGTAATACGGTTATCCACAGAATCAGGGGATAACGCAGGAAAGAACATGTG

AGCAAAAGGCCAGCAAAAGGCCAGGAACCGTAAAAAGGCCGCGTTGCTGGCGTTTTTCCATAGGCTCCGC

CCCCCTGACGAGCATCACAAAAATCGACGCTCAAGTCAGAGGTGGCGAAACCCGACAGGACTATAAAGAT

ACCAGGCGTTTCCCCCTGGAAGCTCCCTCGTGCGCTCTCCTGTTCCGACCCTGCCGCTTACCGGATACCT

GTCCGCCTTTCTCCCTTCGGGAAGCGTGGCGCTTTCTCAATGCTCACGCTGTAGGTATCTCAGTTCGGTG

TAGGTCGTTCGCTCCAAGCTGGGCTGTGTGCACGAACCCCCCGTTCAGCCCGACCGCTGCGCCTTATCCG

GTAACTATCGTCTTGAGTCCAACCCGGTAAGACACGACTTATCGCCACTGGCAGCAGCCACTGGTAACAG

GATTAGCAGAGCGAGGTATGTAGGCGGTGCTACAGAGTTCTTGAAGTGGTGGCCTAACTACGGCTACACT

AGAAGGACAGTATTTGGTATCTGCGCTCTGCTGAAGCCAGTTACCTTCGGAAAAAGAGTTGGTAGCTCTT

GATCCGGCAAACAAACCACCGCTGGTAGCGGTGGTTTTTTTGTTTGCAAGCAGCAGATTACGCGCAGAAA

AAAAGGATATCAAGAAGATCCTTTGATCTTTTCTACGGGGTCTGACGCTCAGTGGAACGAAAACTCACGT

TAAGGGATTTTGGTCATGAGATTATCAAAAAGGATCTTCACCTAGATCCTTTTAAATTAAAAATGAAGTT

TTAAATCAATCTAAAGTATATATGAGTAAACTTGGTCTGACAGTTACCAATGCTTAATCAGTGAGGCACC

TATCTCAGCGATCTGTCTATTTCGTTCATCCATAGTTGCCTGACTCCCCGTCGTGTAGATAACTACGATA

CGGGAGGGCTTACCATCTGGCCCCAGTGCTGCAATGATACCGCGAGACCCACGCTCACCGGCTCCAGATT

TATCAGCAATAAACCAGCCAGCCGGAAGGGCCGAGCGCAGAAGTGGTCCTGCAACTTTATCCGCCTCCAT

CCAGTCTATTAAACAAGTGGCAGCAACGGATTCGCAAACCTGTCACGCCTTTTGTGCCAAAAGCCGCGCC

AGGTTTGCGATCCGCTGTGCCAGGCGTTAGGCGTCATATGAAGATTTCGGTGATCCCTGAGCAGGTGGCG

GAAACATTGGATGCTGAGAACCATTTCATTGTTCGTGAAGTGTTCGATGTGCACCTATCCGACCAAGGCT

TTGAACTATCTACCAGAAGTGTGAGCCCCTACCGGAAGGATTACATCTCGGATGATGACTCTGATGAAGA

CTCTGCTTGCTATGGCGCATTCATCGACCAAGAGCTTGTCGGGAAGATTGAACTCAACTCAACATGGAAC

GATCTAGCCTCTATCGAACACATTGTTGTGTCGCACACGCACCGAGGCAAAGGAGTCGCGCACAGTCTCA

TCGAATTTGCGAAAAAGTGGGCACTAAGCAGACAGCTCCTTGGCATACGATTAGAGACACAAACGAACAA

TGTACCTGCCTGCAATTTGTACGCAAAATGTGGCTTTACTCTCGGCGGCATTGACCTGTTCACGTATAAA

ACTAGACCTCAAGTCTCGAACGAAACAGCGATGTACTGGTACTGGTTCTCGGGAGCACAGGATGACGCCT

AACAATTCATTCAAGCCGACACCGCTTCGCGGCGCGGCTTAATTCAGGAGTTAAACATCATGAGGGAAGC

GGTGATCGCCGAAGTATCGACTCAACTATCAGAGGTAGTTGGCGTCATCGAGCGCCATCTCGAACCGACG

TTGCTGGCCGTACATTTGTACGGCTCCGCAGTGGATGGCGGCCTGAAGCCACACAGTGATATTGATTTGC

TGGTTACGGTGACCGTAAGGCTTGATGAAACAACGCGGCGAGCTTTGATCAACGACCTTTTGGAAACTTC

GGCTTCCCCTGGAGAGAGCGAGATTCTCCGCGCTGTAGAAGTCACCATTGTTGTGCACGACGACATCATT

CCGTGGCGTTATCCAGCTAAGCGCGAACTGCAATTTGGAGAATGGCAGCGCAATGACATTCTTGCAGGTA

TCTTCGAGCCAGCCACGATCGACATTGATCTGGCTATCTTGCTGACAAAAGCAAGAGAACATAGCGTTGC

CTTGGTAGGTCCAGCGGCGGAGGAACTCTTTGATCCGGTTCCTGAACAGGATCTATTTGAGGCGCTAAAT

GAAACCTTAACGCTATGGAACTCGCCGCCCGACTGGGCTGGCGATGAGCGAAATGTAGTGCTTACGTTGT

CCCGCATTTGGTACAGCGCAGTAACCGGCAAAATCGCGCCGAAGGATGTCGCTGCCGACTGGGCAATGGA

GCGCCTGCCGGCCCAGTATCAGCCCGTCATACTTGAAGCTAGGCAGGCTTATCTTGGACAAGAAGATCGC

TTGGCCTCGCGCGCAGATCAGTTGGAAGAATTTGTTCACTACGTGAAAGGCGAGATCACCAAGGTAGTCG

GCAAATAATGTCTAACAATTCGTTCAAGCCGACGCCGCTTCGCGGCGCGGCTTAACTCAAGCGTTAGAGA

GCTGGGGAAGACTATGCGCGATCTGTTGAAGGTGGTTCTAAGCCTCGTACTTGCGATGGCATCGGGGCAG

GCACTTGCTGACCTGCCAATTGTTTTAGTGGATGAAGCTCGTCTTCCCTATGACTACTCCCCATCCAACT

ACGACATTTCTCCAAGCAACTACGACAACTCCATAAGCAATTACGACAATAGTCCATCAAATTACGACAA

CTCTGAGAGCAACTACGATAATAGTTCATCCAATTACGACAATAGTCGCAACGGAAATCGTAGGCTTATA

TATAGCGCAAATGGGTCTCGCACTTTCGCCGGCTACTACGTCATTGCCAACAATGGGACAACGAACTTCT

TTTCCACATCTGGCAAAAGGATGTTCTACACCCCAAAAGGGGGGCGCGGCGTCTATGGCGGCAAAGATGG

GAGCTTCTGCGGGGCATTGGTCGTCATAAATGGCCAATTTTCGCTTGCCCTGACAGATAACGGCCTGAAG

ATCATGTATCTAAGCAACTAGCCTGCTCTCTAATAAAATGTTAGGAGCTTGGCTGCCATTTTTGGGGTGA

GGCCGTTCGCGGCCGAGGGGCGCAGCCCCTGGGGGGATGGGAGGCCCGCGTTAGCGGGCCGGGAGGGTTC

GAGAAGGGGGGGCACCCCCCTTCGGCGTGCGCGGTCACGCGCCAGGGCGCAGCCCTGGTTAAAAACAAGG

TTTATAAATATTGGTTTAAAAGCAGGTTAAAAGACAGGTTAGCGGTGGCCGAAAAACGGGCGGAAACCCT

TGCAAATGCTGGATTTTCTGCCTGTGGACAGCCCCTCAAATGTCAATAGGTGCGCCCCTCATCTGTCAGC

ACTCTGCCCCTCAAGTGTCAAGGATCGCGCCCCTCATCTGTCAGTAGTCGCGCCCCTCAAGTGTCAATAC

CGCAGGGCACTTATCCCCAGGCTTGTCCACATCATCTGTGGGAAACTCGCGTAAAATCAGGCGTTTTCGC

CGATTTGCGAGGCTGGCCAGCTCCACGTCGCCGGCCGAAATCGAGCCTGCCCCTCATCTGTCAACGCCGC

GCCGGGTGAGTCGGCCCCTCAAGTGTCAACGTCCGCCCCTCATCTGTCAGTGAGGGCCAAGTTTTCCGCG

AGGTATCCACAACGCCGGCGGCCGGCCGCGGTGTCTCGCACACGGCTTCGACGGCGTTTCTGGCGCGTTT

GCAGGGCCATAGACGGCCGCCAGCCCAGCGGCGAGGGCAACCAGCCCGGTGAGCGTCGGAAAGGGTCGAC

ATCTTGCTGCGTTCGGATATTTTCGTGGAGTTCCCGCCACAGACCCGGATTGAAGGCGAGATCCAGCAAC

TCGCGCCAGATCATCCTGTGACGGAACTTTGGCGCGTGATGACTGGCCAGGACGTCGGCCGAAAGAGCGA

CAAGCAGATCACGATTTTCGACAGCGTCGGATTTGCGATCGAGGATTTTTCGGCGCTGCGCTACGTCCGC

GACCGCGTTGAGGGATCAAGCCACAGCAGCCCACTCGACCTTCTAGCCGACCCAGACGAGCCAAGGGATC

TTTTTGGAATGCTGCTCCGTCGTCAGGCTTTCCGACGTTTGGGTGGTTGAACAGAAGTCATTATCGTACG

GAATGCCAGCACTCCCGAGGGGAACCCTGTGGTTGGCATGCACATACAAATGGACGAACGGATAAACCTT

TTCACGCCCTTTTAAATATCCGTTATTCTAATAAACGCTCTTTTCTCTTAGGTTTACCCGCCAATATATC

CTGTCAAACACTGATAGTTTAAACTGAAGGCGGGAAACGACAATCTGATCATGAGCGGAGAATTAAGGGA

GTCACGTTATGACCCCCGCCGATGACGCGGGACAAGCCGTTTTACGTTTGGAACTGACAGAACCGCAACG

ATTGAAGGAGCCACTCAGCCCCAATACGCAAACCGCCTCTCCCCGCGCGTTGGCCGATTCATTAATGCAG

CTGGCACGACAGGTTTCCCGACTGGAAAGCGGGCAGTGAGCGCAACGCAATTAATGTGAGTTAGCTCACT

CATTAGGCACCCCAGGCTTTACACTTTATGCTTCCGGCTCGTATGTTGTGTGGAATTGTGAGCGGATAAC

AATTTCACACAGGAAACAGCTATGACCATGATTACGCCAAGCTATTTAGGTGACACTATAGAATACTCAA

GCTATGCATCCAACGCGTTGGGAGCTCTCCCATATCGACCTGCAGGCGGCCGCTCGACGAATTAATTCCA

ATCCCACAAAAATCTGAGCTTAACAGCACAGTTGCTCCTCTCAGAGCAGAATCGGGTATTCAACACCCTC

ATATCAACTACTACGTTGTGTATAACGGTCCACATGCCGGTATATACGATGACTGGGGTTGTACAAAGGC

GGCAACAAACGGCGTTCCCGGAGTTGCACACAAGAAATTTGCCACTATTACAGAGGCAAGAGCAGCAGCT

GACGCGTACACAACAAGTCAGCAAACAGACAGGTTGAACTTCATCCCCAAAGGAGAAGCTCAACTCAAGC

CCAAGAGCTTTGCTAAGGCCCTAACAAGCCCACCAAAGCAAAAAGCCCACTGGCTCACGCTAGGAACCAA

AAGGCCCAGCAGTGATCCAGCCCCAAAAGAGATCTCCTTTGCCCCGGAGATTACAATGGACGATTTCCTC

TATCTTTACGATCTAGGAAGGAAGTTCGAAGGTGAAGGTGACGACACTATGTTCACCACTGATAATGAGA

AGGTTAGCCTCTTCAATTTCAGAAAGAATGCTGACCCACAGATGGTTAGAGAGGCCTACGCAGCAGGTCT

CATCAAGACGATCTACCCGAGTAACAATCTCCAGGAGATCAAATACCTTCCCAAGAAGGTTAAAGATGCA

GTCAAAAGATTCAGGACTAATTGCATCAAGAACACAGAGAAAGACATATTTCTCAAGATCAGAAGTACTA

TTCCAGTATGGACGATTCAAGGCTTGCTTCATAAACCAAGGCAAGTAATAGAGATTGGAGTCTCTAAAAA

GGTAGTTCCTACTGAATCTAAGGCCATGCATGGAGTCTAAGATTCAAATCGAGGATCTAACAGAACTCGC

CGTGAAGACTGGCGAACAGTTCATACAGAGTCTTTTACGACTCAATGACAAGAAGAAAATCTTCGTCAAC

ATGGTGGAGCACGACACTCTGGTCTACTCCAAAAATGTCAAAGATACAGTCTCAGAAGACCAAAGGGCTA

TTGAGACTTTTCAACAAAGGATAATTTCGGGAAACCTCCTCGGATTCCATTGCCCAGCTATCTGTCACTT

CATCGAAAGGACAGTAGAAAAGGAAGGTGGCTCCTACAAATGCCATCATTGCGATAAAGGAAAGGCTATC

ATTCAAGATCTCTCTGCCGACAGTGGTCCCAAAGATGGACCCCCACCCACGAGGAGCATCGTGGAAAAAG

AAGACGTTCCAACCACGTCTTCAAAGCAAGTGGATTGATGTGACATCTCCACTGACGTAAGGGATGACGC

ACAATCCCACTATCCTTCGCAAGACCCTTCCTCTATATAAGGAAGTTCATTTCATTTGGAGAGGACACGC

TCGACTATTTTTACAACAATTACC**ATGAAGACTAATCTTTTTCTCTTTCTCATCTTTTCACTTCTCCTAT**

**CATTATCCTCGGCCGAATTCCTCGAG**TCAC**CAGGCAAAGAATGGCGTCTTTA***GGGATTCACAAATGTTTT*

*CTCA***TCTAGA**AGTAAAGGAGAAGAACTTTTCACTGGAGTTGTCCCAATTCTTGTTGAATTAGATGGTGAT

GTTAATGGGTACAAATTTTCTGTCAGTGGAGAGGGTGAAGGTGATGCAACATACGGAAAACTTACCCTTA

AATTTATTTGCACTACTGGAAAACTACCTGTTCCATGGCCAACACTTGTCACTACTTTCTCTTATGGTGT

TCAATGCTTTTCAAGATACCCAGATCATATGAAGCGGCACGACTTCTTCAAGAGCGCCATGCCTGAGGGA

TACGTGCAGGAGAGGACCATCTTCTTCAAGGACGACGGGAACTACAAGACACGTGCTGAAGTCAAGTTTG

AGGGAGACACCCTCGTCAACAGGATCGAGCTTAAGGGAATCGATTTCAAGGAGGACGGAAACATCCTCGG

CCACAAGTTGGAATACAACTACAACTCCCACAACGTATACATCATGGCCGACAAGCAAAAGAACGGCATC

AAAGCCAACTTCAAGACCCGCCACAACATCGAAGACGGCGGCGTGCAACTCGCTGATCATTATCAACAAA

ATACTCCAATTGGCGATGGCCCTGTCCTTTTACCAGACAACCATTACCTGTCCACACAATCTGCCCTTTC

GAAAGATCCCAACGAAAAGAGAGACCACATGGTCCTTCTTGAGTTTGTAACAGCTGCTGGGATTACACAT

GGCATGGATGAACTATACAAACATGATGAGCTTTGAACTAGAGTCCTGCTTTAATGAGATATGCGAGACG

CCTATGATCGCATGATATTTGCTTTCAATTCTGTTGTGCACGTTGTAAAAAACCTGAGCATGTGTAGCTC

AGATCCTTACCGCCGGTTTCGGTTCATTCTAATGAATATATCACCCGTTACTATCGTATTTTTATGAATA

ATATTCTCCGTTCAATTTACTGATTGTACCCTACTACTTATATGTACAATATTAAAATGAAAACAATATA

TTGTGCTGAATAGGTTTATAGCGACATCTATGATAGAGCGCCACAATAACAAACAATTGCGTTTTATTAT

TACAAATCCAATTTTAAAAAAAGCGGCAGAACCGGTCAAACCTAAAAGACTGATTACATAAATCTTATTC

AAATTTCAAAAGGCCCCAGGGGCTAGTATCTACGACACACCGAGCGGCGAACTAATAACGTTCACTGAAG

GGAACTCCGGTTCCCCGCCGGCGCGCATGGGTGAGATTCCTTGAAGTTGAGTATTGGCCGTCCGCTCTAC

CGAAAGTTACGGGCACCATTCAACCCGGTCCAGCACGGCGGCCGGGTAACCGACTTGCTGCCCCGAGAAT

TATGCAGCATTTTTTTGGTGTATGTGGGCCCCAAATGAAGTGCAGGTCAAACCTTGACAGTGACGACAAA

TCGTTGGGCGGGTCCAGGGCGAATTTTGCGACAACATGTCGAGGCTCAGCAGGACCTGCAGGCATGCAAG

CTAGCTTACTAGTGATGCATATTCTATAGTGTCACCTAAATCTGC

>pMS4-AMIRC4 (**AK0065 (1bp - 14675bp, direct) 14675bp**)

GGCCGCACTAGTGATATCCCGCGGCCATGGCGGCCGGGAGCATGCGACGTCGGGCCCAATTCGCCCTATA

GTGAGTCGTATTACAATTCACTGGCCGTCGTTTTACAACGTCGTGACTGGGAAAACCCTGGCGTTACCCA

ACTTAATCGCCTTGCAGCACATCCCCCTTTCGCCAGCTGGCGTAATAGCGAAGAGGCCCGCACCGATCGC

CCTTCCCAACAGTTGCGCAGCCTGAATGGCGAATGGAAATTGTAAACGTTAATGGGTTTCTGGAGTTTAA

TGAGCTAAGCACATACGTCAGAAACCATTATTGCGCGTTCAAAAGTCGCCTAAGGTCACTATCAGCTAGC

AAATATTTCTTGTCAAAAATGCTCCACTGACGTTCCATAAATTCCCCTCGGTATCCAATTAGAGTCTCAT

ATTCACTCTCAATCCAAATAATCTGCAATGGCAATTACCTTATCCGCAACTTCTTTACCTATTTCCGCCC

GGATCCGGGCAGGTTCTCCGGCCGCTTGGGTGGAGAGGCTATTCGGCTATGACTGGGCACAACAGACAAT

CGGCTGCTCTGATGCCGCCGTGTTCCGGCTGTCAGCGCAGGGGCGCCCGGTTCTTTTTGTCAAGACCGAC

CTGTCCGGTGCCCTGAATGAACTGCAGGACGAGGCAGCGCGGCTATCGTGGCTGGCCACGACGGGCGTTC

CTTGCGCAGCTGTGCTCGACGTTGTCACTGAAGCGGGAAGGGACTGGCTGCTATTGGGCGAAGTGCCGGG

GCAGGATCTCCTGTCATCTCACCTTGCTCCTGCCGAGAAAGTATCCATCATGGCTGATGCAATGCGGCGG

CTGCATACGCTTGATCCGGCTACCTGCCCATTCGACCACCAAGCGAAACATCGCATCGAGCGAGCACGTA

CTCGGATGGAAGCCGGTCTTGTCGATCAGGATGATCTGGACGAAGAGCATCAGGGGCTCGCGCCAGCCGA

ACTGTTCGCCAGGCTCAAGGCGCGCATGCCCGACGGCGAGGATCTCGTCGTGACCCATGGCGATGCCTGC

TTGCCGAATATCATGGTGGAAAATGGCCGCTTTTCTGGATTCATCGACTGTGGCCGGCTGGGTGTGGCGG

ACCGCTATCAGGACATAGCGTTGGCTACCCGTGATATTGCTGAAGAGCTTGGCGGCGAATGGGCTGACCG

CTTCCTCGTGCTTTACGGTATCGCCGCTCCCGATTCGCAGCGCATCGCCTTCTATCGCCTTCTTGACGAG

TTCTTCTGAGCGGGACTCTGGGGTTCGAAATGACCGACCAAGCGACGCCCAACCTGCCATCACGAGATTT

CGATTCCACCGCCGCCTTCTATGAAAGGTTGGGCTTCGGAATCGTTTTCCGGGACGCCGGCTGGATGATC

CTCCAGCGCGGGGATCTCATGCTGGAGTTCTTCGCCCACCCCGATCCAACACTTACGTTTGCAACGTCCA

AGAGCAAATAGACCACGAACGCCGGAAGGTTGCCGCAGCGTGTGGATTGCGTCTCAATTCTCTCTTGCAG

GAATGCAATGATGAATATGATACTGACTATGAAACTTTGAGGGAATACTGCCTAGCACCGTCACCTCATA

ACGTGCATCATGCATGCCCTGACAACATGGAACATCGCTATTTTTCTGAAGAATTATGCTCGTTGGAGGA

TGTCGCGGCAATTGCAGCTATTGCCAACATCGAACTACCCCTCACGCATGCATTCATCAATATTATTCAT

GCGGGGAAAGGCAAGATTAATCCAACTGGCAAATCATCCAGCGTGATTGGTAACTTCAGTTCCAGCGACT

TGATTCGTTTTGGTGCTACCCACGTTTTCAATAAGGACGAGATGGTGGAGTAAAGAAGGAGTGCGTCGAA

GCAGATCGTTCAAACATTTGGCAATAAAGTTTCTTAAGATTGAATCCTGTTGCCGGTCTTGCGATGATTA

TCATATAATTTCTGTTGAATTACGTTAAGCATGTAATAATTAACATGTAATGCATGACGTTATTTATGAG

ATGGGTTTTTATGATTAGAGTCCCGCAATTATACATTTAATACGCGATAGAAAACAAAATATAGCGCGCA

AACTAGGATAAATTATCGCGCGCGGTGTCATCTATGTTACTAGATCGAATTAATTCCAGGCGGTGAAGGG

CAATCAGCTGTTGCCCGTCTCACTGGTGAAAAGAAAAACCACCCCAGTACATTAAAAACGTCCGCAATGT

GTTATTAAGTTGTCTAAGCGTCAATTTGTTTACACCACAATATATCCTGCCACCAGCCAGCCAACAGCTC

CCCGACCGGCAGCTCGGCACAAAATCACCACTCGATACAGGCAGCCCATCAGTCCGGGACGGCGTCAGCG

GGAGAGCCGTTGTAAGGCGGCAGACTTTGCTCATGTTACCGATGCTATTCGGAAGAACGGCAACTAAGCT

GCCGGGTTTGAAACACGGATGATCTCGCGGAGGGTAGCATGTTGATTGTAACGATGACAGAGCGTTGCTG

CCTGTGATCAAATATCATCTCCCTCGCAGAGATCCGAATTATCAGCCTTCTTATTCATTTCTCGCTTAAC

CGTGACAGGCTGTCGATCTTGAGAACTATGCCGACATAATAGGAAATCGCTGGATAAAGCCGCTGAGGAA

GCTGAGTGGCGCTATTTCTTTAGAAGTGAACGTTGACGATGTCGACGGATCTTTTCCGCTGCATAACCCT

GCTTCGGGGTCATTATAGCGATTTTTTCGGTATATCCATCCTTTTTCGCACGATATACAGGATTTTGCCA

AAGGGTTCGTGTAGACTTTCCTTGGTGTATCCAACGGCGTCAGCCGGGCAGGATAGGTGAAGTAGGCCCA

CCCGCGAGCGGGTGTTCCTTCTTCACTGTCCCTTATTCGCACCTGGCGGTGCTCAACGGGAATCCTGCTC

TGCGAGGCTGGCCGGCTACCGCCGGCGTAACAGATGAGGGCAAGCGGATGGCTGATGAAACCAAGCCAAC

CAGGGGTGATGCTGCCAACTTACTGATTTAGTGTATGATGGTGTTTTTGAGGTGCTCCAGTGGCTTCTGT

TTCTATCAGCTGTCCCTCCTGTTCAGCTACTGACGGGGTGGTGCGTAACGGCAAAAGCACCGCCGGACAT

CAGCGCTATCTCTGCTCTCACTGCCGTAAAACATGGCAACTGCAGTTCACTTACACCGCTTCTCAACCCG

GTACGCACCAGAAAATCATTGATATGGCCATGAATGGCGTTGGATGCCGGGCAACAGCCCGCATTATGGG

CGTTGGCCTCAACACGATTTTACGTCACTTAAAAAACTCAGGCCGCAGTCGGTAACCTCGCGCATACAGC

CGGGCAGTGACGTCATCGTCTGCGCGGAAATGGACGAACAGTGGGGCTATGTCGGGGCTAAATCGCGCCA

GCGCTGGCTGTTTTACGCGTATGACAGTCTCCGGAAGACGGTTGTTGCGCACGTATTCGGTGAACGCACT

ATGGCGACGCTGGGGCGTCTTATGAGCCTGCTGTCACCCTTTGACGTGGTGATATGGATGACGGATGGCT

GGCCGCTGTATGAATCCCGCCTGAAGGGAAAGCTGCACGTAATCAGCAAGCGATATACGCAGCGAATTGA

GCGGCATAACCTGAATCTGAGGCAGCACCTGGCACGGCTGGGACGGAAGTCGCTGTCGTTCTCAAAATCG

GTGGAGCTGCATGACAAAGTCATCGGGCATTATCTGAACATAAAACACTATCAATAAGTTGGAGTCATTA

CCCAACCAGGAAGGGCAGCCCACCTATCAAGGTGTACTGCCTTCCAGACGAACGAAGAGCGATTGAGGAA

AAGGCGGCGGCGGCCGGCATGAGCCTGTCGGCCTACCTGCTGGCCGTCGGCCAGGGCTACAAAATCACGG

GCGTCGTGGACTATGAGCACGTCCGCGAGCTGGCCCGCATCAATGGCGACCTGGGCCGCCTGGGCGGCCT

GCTGAAACTCTGGCTCACCGACGACCCGCGCACGGCGCGGTTCGGTGATGCCACGATCCTCGCCCTGCTG

GCGAAGATCGAAGAGAAGCAGGACGAGCTTGGCAAGGTCATGATGGGCGTGGTCCGCCCGAGGGCAGAGC

CATGACTTTTTTAGCCGCTAAAACGGCCGGGGGGTGCGCGTGATTGCCAAGCACGTCCCCATGCGCTCCA

TCAAGAAGAGCGACTTCGCGGAGCTGGTATTCGTGCAGGGCAAGATTCGGAATACCAAGTACGAGAAGGA

CGGCCAGACGGTCTACGGGACCGACTTCATTGCCGATAAGGTGGATTATCTGGACACCAAGGCACCAGGC

GGGTCAAATCAGGAATAAGGGCACATTGCCCCGGCGTGAGTCGGGGCAATCCCGCAAGGAGGGTGAATGA

ATCGGACGTTTGACCGGAAGGCATACAGGCAAGAACTGATCGACGCGGGGTTTTCCGCCGAGGATGCCGA

AACCATCGCAAGCCGCACCGTCATGCGTGCGCCCCGCGAAACCTTCCAGTCCGTCGGCTCGATGGTCCAG

CAAGCTACGGCCAAGATCGAGCGCGACAGCGTGCAACTGGCTCCCCCTGCCCTGCCCGCGCCATCGGCCG

CCGTGGAGCGTTCGCGTCGTCTCGAACAGGAGGCGGCAGGTTTGGCGAAGTCGATGACCATCGACACGCG

AGGAACTATGACGACCAAGAAGCGAAAAACCGCCGGCGAGGACCTGGCAAAACAGGTCAGCGAGGCCAAG

CAGGCCGCGTTGCTGAAACACACGAAGCAGCAGATCAAGGAAATGCAGCTTTCCTTGTTCGATATTGCGC

CGTGGCCGGACACGATGCGAGCGATGCCAAACGACACGGCCCGCTCTGCCCTGTTCACCACGCGCAACAA

GAAAATCCCGCGCGAGGCGCTGCAAAACAAGGTCATTTTCCACGTCAACAAGGACGTGAAGATCACCTAC

ACCGGCGTCGAGCTGCGGGCCGACGATGACGAACTGGTGTGGCAGCAGGTGTTGGAGTACGCGAAGCGCA

CCCCTATCGGCGAGCCGATCACCTTCACGTTCTACGAGCTTTGCCAGGACCTGGGCTGGTCGATCAATGG

CCGGTATTACACGAAGGCCGAGGAATGCCTGTCGCGCCTACAGGCGACGGCGATGGGCTTCACGTCCGAC

CGCGTTGGGCACCTGGAATCGGTGTCGCTGCTGCACCGCTTCCGCGTCCTGGACCGTGGCAAGAAAACGT

CCCGTTGCCAGGTCCTGATCGACGAGGAAATCGTCGTGCTGTTTGCTGGCGACCACTACACGAAATTCAT

ATGGGAGAAGTACCGCAAGCTGTCGCCGACGGCCCGACGGATGTTCGACTATTTCAGCTCGCACCGGGAG

CCGTACCCGCTCAAGCTGGAAACCTTCCGCCTCATGTGCGGATCGGATTCCACCCGCGTGAAGAAGTGGC

GCGAGCAGGTCGGCGAAGCCTGCGAAGAGTTGCGAGGCAGCGGCCTGGTGGAACACGCCTGGGTCAATGA

TGACCTGGTGCATTGCAAACGCTAGGGCCTTGTGGGGTCAGTTCCGGCTGGGGGTTCAGCAGCCAGCGCT

TTACTGGCATTTCAGGAACAAGCGGGCACTGCTCGACGCACTTGCTTCGCTCAGTATCGCTCGGGACGCA

CGGCGCGCTCTACGAACTGCCGATAAACAGAGGATTAAAATTGACAATTGTGATTAAGGCTCAGATTCGA

CGGCTTGGAGCGGCCGACGTGCAGGATTTCCGCGAGATCCGATTGTCGGCCCTGAAGAAAGCTCCAGAGA

TGTTCGGGTCCGTTTACGAGCACGAGGAGAAAAAGCCCATGGAGGCGTTCGCTGAACGGTTGCGAGATGC

CGTGGCATTCGGCGCCTACATCGACGGCGAGATCATTGGGCTGTCGGTCTTCAAACAGGAGGACGGCCCC

AAGGACGCTCACAAGGCGCATCTGTCCGGCGTTTTCGTGGAGCCCGAACAGCGAGGCCGAGGGGTCGCCG

GTATGCTGCTGCGGGCGTTGCCGGCGGGTTTATTGCTCGTGATGATCGTCCGACAGATTCCAACGGGAAT

CTGGTGGATGCGCATCTTCATCCTCGGCGCACTTAATATTTCGCTATTCTGGAGCTTGTTGTTTATTTCG

GTCTACCGCCTGCCGGGCGGGGTCGCGGCGACGGTAGGCGCTGTGCAGCCGCTGATGGTCGTGTTCATCT

CTGCCGCTCTGCTAGGTAGCCCGATACGATTGATGGCGGTCCTGGGGGCTATTTGCGGAACTGCGGGCGT

GGCGCTGTTGGTGTTGACACCAAACGCAGCGCTAGATCCTGTCGGCGTCGCAGCGGGCCTGGCGGGGGCG

GTTTCCATGGCGTTCGGAACCGTGCTGACCCGCAAGTGGCAACCTCCCGTGCCTCTGCTCACCTTTACCG

CCTGGCAACTGGCGGCCGGAGGACTTCTGCTCGTTCCAGTAGCTTTAGTGTTTGATCCGCCAATCCCGAT

GCCTACAGGAACCAATGTTCTCGGCCTGGCGTGGCTCGGCCTGATCGGAGCGGGTTTAACCTACTTCCTT

TGGTTCCGGGGGATCTCGCGACTCGAACCTACAGTTGTTTCCTTACTGGGCTTTCTCAGCCGGGATGGCG

CTAAGAAGCTATTGCCGCCGATCTTCATATGCGGTGTGAAATACCGCACAGATGCGTAAGGAGAAAATAC

CGCATCAGGCGCTCTTCCGCTTCCTCGCTCACTGACTCGCTGCGCTCGGTCGTTCGGCTGCGGCGAGCGG

TATCAGCTCACTCAAAGGCGGTAATACGGTTATCCACAGAATCAGGGGATAACGCAGGAAAGAACATGTG

AGCAAAAGGCCAGCAAAAGGCCAGGAACCGTAAAAAGGCCGCGTTGCTGGCGTTTTTCCATAGGCTCCGC

CCCCCTGACGAGCATCACAAAAATCGACGCTCAAGTCAGAGGTGGCGAAACCCGACAGGACTATAAAGAT

ACCAGGCGTTTCCCCCTGGAAGCTCCCTCGTGCGCTCTCCTGTTCCGACCCTGCCGCTTACCGGATACCT

GTCCGCCTTTCTCCCTTCGGGAAGCGTGGCGCTTTCTCAATGCTCACGCTGTAGGTATCTCAGTTCGGTG

TAGGTCGTTCGCTCCAAGCTGGGCTGTGTGCACGAACCCCCCGTTCAGCCCGACCGCTGCGCCTTATCCG

GTAACTATCGTCTTGAGTCCAACCCGGTAAGACACGACTTATCGCCACTGGCAGCAGCCACTGGTAACAG

GATTAGCAGAGCGAGGTATGTAGGCGGTGCTACAGAGTTCTTGAAGTGGTGGCCTAACTACGGCTACACT

AGAAGGACAGTATTTGGTATCTGCGCTCTGCTGAAGCCAGTTACCTTCGGAAAAAGAGTTGGTAGCTCTT

GATCCGGCAAACAAACCACCGCTGGTAGCGGTGGTTTTTTTGTTTGCAAGCAGCAGATTACGCGCAGAAA

AAAAGGATATCAAGAAGATCCTTTGATCTTTTCTACGGGGTCTGACGCTCAGTGGAACGAAAACTCACGT

TAAGGGATTTTGGTCATGAGATTATCAAAAAGGATCTTCACCTAGATCCTTTTAAATTAAAAATGAAGTT

TTAAATCAATCTAAAGTATATATGAGTAAACTTGGTCTGACAGTTACCAATGCTTAATCAGTGAGGCACC

TATCTCAGCGATCTGTCTATTTCGTTCATCCATAGTTGCCTGACTCCCCGTCGTGTAGATAACTACGATA

CGGGAGGGCTTACCATCTGGCCCCAGTGCTGCAATGATACCGCGAGACCCACGCTCACCGGCTCCAGATT

TATCAGCAATAAACCAGCCAGCCGGAAGGGCCGAGCGCAGAAGTGGTCCTGCAACTTTATCCGCCTCCAT

CCAGTCTATTAAACAAGTGGCAGCAACGGATTCGCAAACCTGTCACGCCTTTTGTGCCAAAAGCCGCGCC

AGGTTTGCGATCCGCTGTGCCAGGCGTTAGGCGTCATATGAAGATTTCGGTGATCCCTGAGCAGGTGGCG

GAAACATTGGATGCTGAGAACCATTTCATTGTTCGTGAAGTGTTCGATGTGCACCTATCCGACCAAGGCT

TTGAACTATCTACCAGAAGTGTGAGCCCCTACCGGAAGGATTACATCTCGGATGATGACTCTGATGAAGA

CTCTGCTTGCTATGGCGCATTCATCGACCAAGAGCTTGTCGGGAAGATTGAACTCAACTCAACATGGAAC

GATCTAGCCTCTATCGAACACATTGTTGTGTCGCACACGCACCGAGGCAAAGGAGTCGCGCACAGTCTCA

TCGAATTTGCGAAAAAGTGGGCACTAAGCAGACAGCTCCTTGGCATACGATTAGAGACACAAACGAACAA

TGTACCTGCCTGCAATTTGTACGCAAAATGTGGCTTTACTCTCGGCGGCATTGACCTGTTCACGTATAAA

ACTAGACCTCAAGTCTCGAACGAAACAGCGATGTACTGGTACTGGTTCTCGGGAGCACAGGATGACGCCT

AACAATTCATTCAAGCCGACACCGCTTCGCGGCGCGGCTTAATTCAGGAGTTAAACATCATGAGGGAAGC

GGTGATCGCCGAAGTATCGACTCAACTATCAGAGGTAGTTGGCGTCATCGAGCGCCATCTCGAACCGACG

TTGCTGGCCGTACATTTGTACGGCTCCGCAGTGGATGGCGGCCTGAAGCCACACAGTGATATTGATTTGC

TGGTTACGGTGACCGTAAGGCTTGATGAAACAACGCGGCGAGCTTTGATCAACGACCTTTTGGAAACTTC

GGCTTCCCCTGGAGAGAGCGAGATTCTCCGCGCTGTAGAAGTCACCATTGTTGTGCACGACGACATCATT

CCGTGGCGTTATCCAGCTAAGCGCGAACTGCAATTTGGAGAATGGCAGCGCAATGACATTCTTGCAGGTA

TCTTCGAGCCAGCCACGATCGACATTGATCTGGCTATCTTGCTGACAAAAGCAAGAGAACATAGCGTTGC

CTTGGTAGGTCCAGCGGCGGAGGAACTCTTTGATCCGGTTCCTGAACAGGATCTATTTGAGGCGCTAAAT

GAAACCTTAACGCTATGGAACTCGCCGCCCGACTGGGCTGGCGATGAGCGAAATGTAGTGCTTACGTTGT

CCCGCATTTGGTACAGCGCAGTAACCGGCAAAATCGCGCCGAAGGATGTCGCTGCCGACTGGGCAATGGA

GCGCCTGCCGGCCCAGTATCAGCCCGTCATACTTGAAGCTAGGCAGGCTTATCTTGGACAAGAAGATCGC

TTGGCCTCGCGCGCAGATCAGTTGGAAGAATTTGTTCACTACGTGAAAGGCGAGATCACCAAGGTAGTCG

GCAAATAATGTCTAACAATTCGTTCAAGCCGACGCCGCTTCGCGGCGCGGCTTAACTCAAGCGTTAGAGA

GCTGGGGAAGACTATGCGCGATCTGTTGAAGGTGGTTCTAAGCCTCGTACTTGCGATGGCATCGGGGCAG

GCACTTGCTGACCTGCCAATTGTTTTAGTGGATGAAGCTCGTCTTCCCTATGACTACTCCCCATCCAACT

ACGACATTTCTCCAAGCAACTACGACAACTCCATAAGCAATTACGACAATAGTCCATCAAATTACGACAA

CTCTGAGAGCAACTACGATAATAGTTCATCCAATTACGACAATAGTCGCAACGGAAATCGTAGGCTTATA

TATAGCGCAAATGGGTCTCGCACTTTCGCCGGCTACTACGTCATTGCCAACAATGGGACAACGAACTTCT

TTTCCACATCTGGCAAAAGGATGTTCTACACCCCAAAAGGGGGGCGCGGCGTCTATGGCGGCAAAGATGG

GAGCTTCTGCGGGGCATTGGTCGTCATAAATGGCCAATTTTCGCTTGCCCTGACAGATAACGGCCTGAAG

ATCATGTATCTAAGCAACTAGCCTGCTCTCTAATAAAATGTTAGGAGCTTGGCTGCCATTTTTGGGGTGA

GGCCGTTCGCGGCCGAGGGGCGCAGCCCCTGGGGGGATGGGAGGCCCGCGTTAGCGGGCCGGGAGGGTTC

GAGAAGGGGGGGCACCCCCCTTCGGCGTGCGCGGTCACGCGCCAGGGCGCAGCCCTGGTTAAAAACAAGG

TTTATAAATATTGGTTTAAAAGCAGGTTAAAAGACAGGTTAGCGGTGGCCGAAAAACGGGCGGAAACCCT

TGCAAATGCTGGATTTTCTGCCTGTGGACAGCCCCTCAAATGTCAATAGGTGCGCCCCTCATCTGTCAGC

ACTCTGCCCCTCAAGTGTCAAGGATCGCGCCCCTCATCTGTCAGTAGTCGCGCCCCTCAAGTGTCAATAC

CGCAGGGCACTTATCCCCAGGCTTGTCCACATCATCTGTGGGAAACTCGCGTAAAATCAGGCGTTTTCGC

CGATTTGCGAGGCTGGCCAGCTCCACGTCGCCGGCCGAAATCGAGCCTGCCCCTCATCTGTCAACGCCGC

GCCGGGTGAGTCGGCCCCTCAAGTGTCAACGTCCGCCCCTCATCTGTCAGTGAGGGCCAAGTTTTCCGCG

AGGTATCCACAACGCCGGCGGCCGGCCGCGGTGTCTCGCACACGGCTTCGACGGCGTTTCTGGCGCGTTT

GCAGGGCCATAGACGGCCGCCAGCCCAGCGGCGAGGGCAACCAGCCCGGTGAGCGTCGGAAAGGGTCGAC

ATCTTGCTGCGTTCGGATATTTTCGTGGAGTTCCCGCCACAGACCCGGATTGAAGGCGAGATCCAGCAAC

TCGCGCCAGATCATCCTGTGACGGAACTTTGGCGCGTGATGACTGGCCAGGACGTCGGCCGAAAGAGCGA

CAAGCAGATCACGATTTTCGACAGCGTCGGATTTGCGATCGAGGATTTTTCGGCGCTGCGCTACGTCCGC

GACCGCGTTGAGGGATCAAGCCACAGCAGCCCACTCGACCTTCTAGCCGACCCAGACGAGCCAAGGGATC

TTTTTGGAATGCTGCTCCGTCGTCAGGCTTTCCGACGTTTGGGTGGTTGAACAGAAGTCATTATCGTACG

GAATGCCAGCACTCCCGAGGGGAACCCTGTGGTTGGCATGCACATACAAATGGACGAACGGATAAACCTT

TTCACGCCCTTTTAAATATCCGTTATTCTAATAAACGCTCTTTTCTCTTAGGTTTACCCGCCAATATATC

CTGTCAAACACTGATAGTTTAAACTGAAGGCGGGAAACGACAATCTGATCATGAGCGGAGAATTAAGGGA

GTCACGTTATGACCCCCGCCGATGACGCGGGACAAGCCGTTTTACGTTTGGAACTGACAGAACCGCAACG

ATTGAAGGAGCCACTCAGCCCCAATACGCAAACCGCCTCTCCCCGCGCGTTGGCCGATTCATTAATGCAG

CTGGCACGACAGGTTTCCCGACTGGAAAGCGGGCAGTGAGCGCAACGCAATTAATGTGAGTTAGCTCACT

CATTAGGCACCCCAGGCTTTACACTTTATGCTTCCGGCTCGTATGTTGTGTGGAATTGTGAGCGGATAAC

AATTTCACACAGGAAACAGCTATGACCATGATTACGCCAAGCTATTTAGGTGACACTATAGAATACTCAA

GCTATGCATCCAACGCGTTGGGAGCTCTCCCATATCGACCTGCAGGCGGCCGCTCGACGAATTAATTCCA

ATCCCACAAAAATCTGAGCTTAACAGCACAGTTGCTCCTCTCAGAGCAGAATCGGGTATTCAACACCCTC

ATATCAACTACTACGTTGTGTATAACGGTCCACATGCCGGTATATACGATGACTGGGGTTGTACAAAGGC

GGCAACAAACGGCGTTCCCGGAGTTGCACACAAGAAATTTGCCACTATTACAGAGGCAAGAGCAGCAGCT

GACGCGTACACAACAAGTCAGCAAACAGACAGGTTGAACTTCATCCCCAAAGGAGAAGCTCAACTCAAGC

CCAAGAGCTTTGCTAAGGCCCTAACAAGCCCACCAAAGCAAAAAGCCCACTGGCTCACGCTAGGAACCAA

AAGGCCCAGCAGTGATCCAGCCCCAAAAGAGATCTCCTTTGCCCCGGAGATTACAATGGACGATTTCCTC

TATCTTTACGATCTAGGAAGGAAGTTCGAAGGTGAAGGTGACGACACTATGTTCACCACTGATAATGAGA

AGGTTAGCCTCTTCAATTTCAGAAAGAATGCTGACCCACAGATGGTTAGAGAGGCCTACGCAGCAGGTCT

CATCAAGACGATCTACCCGAGTAACAATCTCCAGGAGATCAAATACCTTCCCAAGAAGGTTAAAGATGCA

GTCAAAAGATTCAGGACTAATTGCATCAAGAACACAGAGAAAGACATATTTCTCAAGATCAGAAGTACTA

TTCCAGTATGGACGATTCAAGGCTTGCTTCATAAACCAAGGCAAGTAATAGAGATTGGAGTCTCTAAAAA

GGTAGTTCCTACTGAATCTAAGGCCATGCATGGAGTCTAAGATTCAAATCGAGGATCTAACAGAACTCGC

CGTGAAGACTGGCGAACAGTTCATACAGAGTCTTTTACGACTCAATGACAAGAAGAAAATCTTCGTCAAC

ATGGTGGAGCACGACACTCTGGTCTACTCCAAAAATGTCAAAGATACAGTCTCAGAAGACCAAAGGGCTA

TTGAGACTTTTCAACAAAGGATAATTTCGGGAAACCTCCTCGGATTCCATTGCCCAGCTATCTGTCACTT

CATCGAAAGGACAGTAGAAAAGGAAGGTGGCTCCTACAAATGCCATCATTGCGATAAAGGAAAGGCTATC

ATTCAAGATCTCTCTGCCGACAGTGGTCCCAAAGATGGACCCCCACCCACGAGGAGCATCGTGGAAAAAG

AAGACGTTCCAACCACGTCTTCAAAGCAAGTGGATTGATGTGACATCTCCACTGACGTAAGGGATGACGC

ACAATCCCACTATCCTTCGCAAGACCCTTCCTCTATATAAGGAAGTTCATTTCATTTGGAGAGGACACGC

TCGACTATTTTTACAACAATTACC**ATGAAGACTAATCTTTTTCTCTTTCTCATCTTTTCACTTCTCCTAT**

**CATTATCCTCGGCCGAATTCCTCGAG**TCAC**CACATTTCCATCCAAACATTCA***GCCAACGACGCATACGCC*

*GAAA***TCTAGA**AGTAAAGGAGAAGAACTTTTCACTGGAGTTGTCCCAATTCTTGTTGAATTAGATGGTGAT

GTTAATGGGTACAAATTTTCTGTCAGTGGAGAGGGTGAAGGTGATGCAACATACGGAAAACTTACCCTTA

AATTTATTTGCACTACTGGAAAACTACCTGTTCCATGGCCAACACTTGTCACTACTTTCTCTTATGGTGT

TCAATGCTTTTCAAGATACCCAGATCATATGAAGCGGCACGACTTCTTCAAGAGCGCCATGCCTGAGGGA

TACGTGCAGGAGAGGACCATCTTCTTCAAGGACGACGGGAACTACAAGACACGTGCTGAAGTCAAGTTTG

AGGGAGACACCCTCGTCAACAGGATCGAGCTTAAGGGAATCGATTTCAAGGAGGACGGAAACATCCTCGG

CCACAAGTTGGAATACAACTACAACTCCCACAACGTATACATCATGGCCGACAAGCAAAAGAACGGCATC

AAAGCCAACTTCAAGACCCGCCACAACATCGAAGACGGCGGCGTGCAACTCGCTGATCATTATCAACAAA

ATACTCCAATTGGCGATGGCCCTGTCCTTTTACCAGACAACCATTACCTGTCCACACAATCTGCCCTTTC

GAAAGATCCCAACGAAAAGAGAGACCACATGGTCCTTCTTGAGTTTGTAACAGCTGCTGGGATTACACAT

GGCATGGATGAACTATACAAACATGATGAGCTTTGAACTAGAGTCCTGCTTTAATGAGATATGCGAGACG

CCTATGATCGCATGATATTTGCTTTCAATTCTGTTGTGCACGTTGTAAAAAACCTGAGCATGTGTAGCTC

AGATCCTTACCGCCGGTTTCGGTTCATTCTAATGAATATATCACCCGTTACTATCGTATTTTTATGAATA

ATATTCTCCGTTCAATTTACTGATTGTACCCTACTACTTATATGTACAATATTAAAATGAAAACAATATA

TTGTGCTGAATAGGTTTATAGCGACATCTATGATAGAGCGCCACAATAACAAACAATTGCGTTTTATTAT

TACAAATCCAATTTTAAAAAAAGCGGCAGAACCGGTCAAACCTAAAAGACTGATTACATAAATCTTATTC

AAATTTCAAAAGGCCCCAGGGGCTAGTATCTACGACACACCGAGCGGCGAACTAATAACGTTCACTGAAG

GGAACTCCGGTTCCCCGCCGGCGCGCATGGGTGAGATTCCTTGAAGTTGAGTATTGGCCGTCCGCTCTAC

CGAAAGTTACGGGCACCATTCAACCCGGTCCAGCACGGCGGCCGGGTAACCGACTTGCTGCCCCGAGAAT

TATGCAGCATTTTTTTGGTGTATGTGGGCCCCAAATGAAGTGCAGGTCAAACCTTGACAGTGACGACAAA

TCGTTGGGCGGGTCCAGGGCGAATTTTGCGACAACATGTCGAGGCTCAGCAGGACCTGCAGGCATGCAAG

CTAGCTTACTAGTGATGCATATTCTATAGTGTCACCTAAATCTGC

>pMS4-AMIRCP (**AK0066 (1bp - 14675bp, direct) 14675bp**)

GGCCGCACTAGTGATATCCCGCGGCCATGGCGGCCGGGAGCATGCGACGTCGGGCCCAATTCGCCCTATA

GTGAGTCGTATTACAATTCACTGGCCGTCGTTTTACAACGTCGTGACTGGGAAAACCCTGGCGTTACCCA

ACTTAATCGCCTTGCAGCACATCCCCCTTTCGCCAGCTGGCGTAATAGCGAAGAGGCCCGCACCGATCGC

CCTTCCCAACAGTTGCGCAGCCTGAATGGCGAATGGAAATTGTAAACGTTAATGGGTTTCTGGAGTTTAA

TGAGCTAAGCACATACGTCAGAAACCATTATTGCGCGTTCAAAAGTCGCCTAAGGTCACTATCAGCTAGC

AAATATTTCTTGTCAAAAATGCTCCACTGACGTTCCATAAATTCCCCTCGGTATCCAATTAGAGTCTCAT

ATTCACTCTCAATCCAAATAATCTGCAATGGCAATTACCTTATCCGCAACTTCTTTACCTATTTCCGCCC

GGATCCGGGCAGGTTCTCCGGCCGCTTGGGTGGAGAGGCTATTCGGCTATGACTGGGCACAACAGACAAT

CGGCTGCTCTGATGCCGCCGTGTTCCGGCTGTCAGCGCAGGGGCGCCCGGTTCTTTTTGTCAAGACCGAC

CTGTCCGGTGCCCTGAATGAACTGCAGGACGAGGCAGCGCGGCTATCGTGGCTGGCCACGACGGGCGTTC

CTTGCGCAGCTGTGCTCGACGTTGTCACTGAAGCGGGAAGGGACTGGCTGCTATTGGGCGAAGTGCCGGG

GCAGGATCTCCTGTCATCTCACCTTGCTCCTGCCGAGAAAGTATCCATCATGGCTGATGCAATGCGGCGG

CTGCATACGCTTGATCCGGCTACCTGCCCATTCGACCACCAAGCGAAACATCGCATCGAGCGAGCACGTA

CTCGGATGGAAGCCGGTCTTGTCGATCAGGATGATCTGGACGAAGAGCATCAGGGGCTCGCGCCAGCCGA

ACTGTTCGCCAGGCTCAAGGCGCGCATGCCCGACGGCGAGGATCTCGTCGTGACCCATGGCGATGCCTGC

TTGCCGAATATCATGGTGGAAAATGGCCGCTTTTCTGGATTCATCGACTGTGGCCGGCTGGGTGTGGCGG

ACCGCTATCAGGACATAGCGTTGGCTACCCGTGATATTGCTGAAGAGCTTGGCGGCGAATGGGCTGACCG

CTTCCTCGTGCTTTACGGTATCGCCGCTCCCGATTCGCAGCGCATCGCCTTCTATCGCCTTCTTGACGAG

TTCTTCTGAGCGGGACTCTGGGGTTCGAAATGACCGACCAAGCGACGCCCAACCTGCCATCACGAGATTT

CGATTCCACCGCCGCCTTCTATGAAAGGTTGGGCTTCGGAATCGTTTTCCGGGACGCCGGCTGGATGATC

CTCCAGCGCGGGGATCTCATGCTGGAGTTCTTCGCCCACCCCGATCCAACACTTACGTTTGCAACGTCCA

AGAGCAAATAGACCACGAACGCCGGAAGGTTGCCGCAGCGTGTGGATTGCGTCTCAATTCTCTCTTGCAG

GAATGCAATGATGAATATGATACTGACTATGAAACTTTGAGGGAATACTGCCTAGCACCGTCACCTCATA

ACGTGCATCATGCATGCCCTGACAACATGGAACATCGCTATTTTTCTGAAGAATTATGCTCGTTGGAGGA

TGTCGCGGCAATTGCAGCTATTGCCAACATCGAACTACCCCTCACGCATGCATTCATCAATATTATTCAT

GCGGGGAAAGGCAAGATTAATCCAACTGGCAAATCATCCAGCGTGATTGGTAACTTCAGTTCCAGCGACT

TGATTCGTTTTGGTGCTACCCACGTTTTCAATAAGGACGAGATGGTGGAGTAAAGAAGGAGTGCGTCGAA

GCAGATCGTTCAAACATTTGGCAATAAAGTTTCTTAAGATTGAATCCTGTTGCCGGTCTTGCGATGATTA

TCATATAATTTCTGTTGAATTACGTTAAGCATGTAATAATTAACATGTAATGCATGACGTTATTTATGAG

ATGGGTTTTTATGATTAGAGTCCCGCAATTATACATTTAATACGCGATAGAAAACAAAATATAGCGCGCA

AACTAGGATAAATTATCGCGCGCGGTGTCATCTATGTTACTAGATCGAATTAATTCCAGGCGGTGAAGGG

CAATCAGCTGTTGCCCGTCTCACTGGTGAAAAGAAAAACCACCCCAGTACATTAAAAACGTCCGCAATGT

GTTATTAAGTTGTCTAAGCGTCAATTTGTTTACACCACAATATATCCTGCCACCAGCCAGCCAACAGCTC

CCCGACCGGCAGCTCGGCACAAAATCACCACTCGATACAGGCAGCCCATCAGTCCGGGACGGCGTCAGCG

GGAGAGCCGTTGTAAGGCGGCAGACTTTGCTCATGTTACCGATGCTATTCGGAAGAACGGCAACTAAGCT

GCCGGGTTTGAAACACGGATGATCTCGCGGAGGGTAGCATGTTGATTGTAACGATGACAGAGCGTTGCTG

CCTGTGATCAAATATCATCTCCCTCGCAGAGATCCGAATTATCAGCCTTCTTATTCATTTCTCGCTTAAC

CGTGACAGGCTGTCGATCTTGAGAACTATGCCGACATAATAGGAAATCGCTGGATAAAGCCGCTGAGGAA

GCTGAGTGGCGCTATTTCTTTAGAAGTGAACGTTGACGATGTCGACGGATCTTTTCCGCTGCATAACCCT

GCTTCGGGGTCATTATAGCGATTTTTTCGGTATATCCATCCTTTTTCGCACGATATACAGGATTTTGCCA

AAGGGTTCGTGTAGACTTTCCTTGGTGTATCCAACGGCGTCAGCCGGGCAGGATAGGTGAAGTAGGCCCA

CCCGCGAGCGGGTGTTCCTTCTTCACTGTCCCTTATTCGCACCTGGCGGTGCTCAACGGGAATCCTGCTC

TGCGAGGCTGGCCGGCTACCGCCGGCGTAACAGATGAGGGCAAGCGGATGGCTGATGAAACCAAGCCAAC

CAGGGGTGATGCTGCCAACTTACTGATTTAGTGTATGATGGTGTTTTTGAGGTGCTCCAGTGGCTTCTGT

TTCTATCAGCTGTCCCTCCTGTTCAGCTACTGACGGGGTGGTGCGTAACGGCAAAAGCACCGCCGGACAT

CAGCGCTATCTCTGCTCTCACTGCCGTAAAACATGGCAACTGCAGTTCACTTACACCGCTTCTCAACCCG

GTACGCACCAGAAAATCATTGATATGGCCATGAATGGCGTTGGATGCCGGGCAACAGCCCGCATTATGGG

CGTTGGCCTCAACACGATTTTACGTCACTTAAAAAACTCAGGCCGCAGTCGGTAACCTCGCGCATACAGC

CGGGCAGTGACGTCATCGTCTGCGCGGAAATGGACGAACAGTGGGGCTATGTCGGGGCTAAATCGCGCCA

GCGCTGGCTGTTTTACGCGTATGACAGTCTCCGGAAGACGGTTGTTGCGCACGTATTCGGTGAACGCACT

ATGGCGACGCTGGGGCGTCTTATGAGCCTGCTGTCACCCTTTGACGTGGTGATATGGATGACGGATGGCT

GGCCGCTGTATGAATCCCGCCTGAAGGGAAAGCTGCACGTAATCAGCAAGCGATATACGCAGCGAATTGA

GCGGCATAACCTGAATCTGAGGCAGCACCTGGCACGGCTGGGACGGAAGTCGCTGTCGTTCTCAAAATCG

GTGGAGCTGCATGACAAAGTCATCGGGCATTATCTGAACATAAAACACTATCAATAAGTTGGAGTCATTA

CCCAACCAGGAAGGGCAGCCCACCTATCAAGGTGTACTGCCTTCCAGACGAACGAAGAGCGATTGAGGAA

AAGGCGGCGGCGGCCGGCATGAGCCTGTCGGCCTACCTGCTGGCCGTCGGCCAGGGCTACAAAATCACGG

GCGTCGTGGACTATGAGCACGTCCGCGAGCTGGCCCGCATCAATGGCGACCTGGGCCGCCTGGGCGGCCT

GCTGAAACTCTGGCTCACCGACGACCCGCGCACGGCGCGGTTCGGTGATGCCACGATCCTCGCCCTGCTG

GCGAAGATCGAAGAGAAGCAGGACGAGCTTGGCAAGGTCATGATGGGCGTGGTCCGCCCGAGGGCAGAGC

CATGACTTTTTTAGCCGCTAAAACGGCCGGGGGGTGCGCGTGATTGCCAAGCACGTCCCCATGCGCTCCA

TCAAGAAGAGCGACTTCGCGGAGCTGGTATTCGTGCAGGGCAAGATTCGGAATACCAAGTACGAGAAGGA

CGGCCAGACGGTCTACGGGACCGACTTCATTGCCGATAAGGTGGATTATCTGGACACCAAGGCACCAGGC

GGGTCAAATCAGGAATAAGGGCACATTGCCCCGGCGTGAGTCGGGGCAATCCCGCAAGGAGGGTGAATGA

ATCGGACGTTTGACCGGAAGGCATACAGGCAAGAACTGATCGACGCGGGGTTTTCCGCCGAGGATGCCGA

AACCATCGCAAGCCGCACCGTCATGCGTGCGCCCCGCGAAACCTTCCAGTCCGTCGGCTCGATGGTCCAG

CAAGCTACGGCCAAGATCGAGCGCGACAGCGTGCAACTGGCTCCCCCTGCCCTGCCCGCGCCATCGGCCG

CCGTGGAGCGTTCGCGTCGTCTCGAACAGGAGGCGGCAGGTTTGGCGAAGTCGATGACCATCGACACGCG

AGGAACTATGACGACCAAGAAGCGAAAAACCGCCGGCGAGGACCTGGCAAAACAGGTCAGCGAGGCCAAG

CAGGCCGCGTTGCTGAAACACACGAAGCAGCAGATCAAGGAAATGCAGCTTTCCTTGTTCGATATTGCGC

CGTGGCCGGACACGATGCGAGCGATGCCAAACGACACGGCCCGCTCTGCCCTGTTCACCACGCGCAACAA

GAAAATCCCGCGCGAGGCGCTGCAAAACAAGGTCATTTTCCACGTCAACAAGGACGTGAAGATCACCTAC

ACCGGCGTCGAGCTGCGGGCCGACGATGACGAACTGGTGTGGCAGCAGGTGTTGGAGTACGCGAAGCGCA

CCCCTATCGGCGAGCCGATCACCTTCACGTTCTACGAGCTTTGCCAGGACCTGGGCTGGTCGATCAATGG

CCGGTATTACACGAAGGCCGAGGAATGCCTGTCGCGCCTACAGGCGACGGCGATGGGCTTCACGTCCGAC

CGCGTTGGGCACCTGGAATCGGTGTCGCTGCTGCACCGCTTCCGCGTCCTGGACCGTGGCAAGAAAACGT

CCCGTTGCCAGGTCCTGATCGACGAGGAAATCGTCGTGCTGTTTGCTGGCGACCACTACACGAAATTCAT

ATGGGAGAAGTACCGCAAGCTGTCGCCGACGGCCCGACGGATGTTCGACTATTTCAGCTCGCACCGGGAG

CCGTACCCGCTCAAGCTGGAAACCTTCCGCCTCATGTGCGGATCGGATTCCACCCGCGTGAAGAAGTGGC

GCGAGCAGGTCGGCGAAGCCTGCGAAGAGTTGCGAGGCAGCGGCCTGGTGGAACACGCCTGGGTCAATGA

TGACCTGGTGCATTGCAAACGCTAGGGCCTTGTGGGGTCAGTTCCGGCTGGGGGTTCAGCAGCCAGCGCT

TTACTGGCATTTCAGGAACAAGCGGGCACTGCTCGACGCACTTGCTTCGCTCAGTATCGCTCGGGACGCA

CGGCGCGCTCTACGAACTGCCGATAAACAGAGGATTAAAATTGACAATTGTGATTAAGGCTCAGATTCGA

CGGCTTGGAGCGGCCGACGTGCAGGATTTCCGCGAGATCCGATTGTCGGCCCTGAAGAAAGCTCCAGAGA

TGTTCGGGTCCGTTTACGAGCACGAGGAGAAAAAGCCCATGGAGGCGTTCGCTGAACGGTTGCGAGATGC

CGTGGCATTCGGCGCCTACATCGACGGCGAGATCATTGGGCTGTCGGTCTTCAAACAGGAGGACGGCCCC

AAGGACGCTCACAAGGCGCATCTGTCCGGCGTTTTCGTGGAGCCCGAACAGCGAGGCCGAGGGGTCGCCG

GTATGCTGCTGCGGGCGTTGCCGGCGGGTTTATTGCTCGTGATGATCGTCCGACAGATTCCAACGGGAAT

CTGGTGGATGCGCATCTTCATCCTCGGCGCACTTAATATTTCGCTATTCTGGAGCTTGTTGTTTATTTCG

GTCTACCGCCTGCCGGGCGGGGTCGCGGCGACGGTAGGCGCTGTGCAGCCGCTGATGGTCGTGTTCATCT

CTGCCGCTCTGCTAGGTAGCCCGATACGATTGATGGCGGTCCTGGGGGCTATTTGCGGAACTGCGGGCGT

GGCGCTGTTGGTGTTGACACCAAACGCAGCGCTAGATCCTGTCGGCGTCGCAGCGGGCCTGGCGGGGGCG

GTTTCCATGGCGTTCGGAACCGTGCTGACCCGCAAGTGGCAACCTCCCGTGCCTCTGCTCACCTTTACCG

CCTGGCAACTGGCGGCCGGAGGACTTCTGCTCGTTCCAGTAGCTTTAGTGTTTGATCCGCCAATCCCGAT

GCCTACAGGAACCAATGTTCTCGGCCTGGCGTGGCTCGGCCTGATCGGAGCGGGTTTAACCTACTTCCTT

TGGTTCCGGGGGATCTCGCGACTCGAACCTACAGTTGTTTCCTTACTGGGCTTTCTCAGCCGGGATGGCG

CTAAGAAGCTATTGCCGCCGATCTTCATATGCGGTGTGAAATACCGCACAGATGCGTAAGGAGAAAATAC

CGCATCAGGCGCTCTTCCGCTTCCTCGCTCACTGACTCGCTGCGCTCGGTCGTTCGGCTGCGGCGAGCGG

TATCAGCTCACTCAAAGGCGGTAATACGGTTATCCACAGAATCAGGGGATAACGCAGGAAAGAACATGTG

AGCAAAAGGCCAGCAAAAGGCCAGGAACCGTAAAAAGGCCGCGTTGCTGGCGTTTTTCCATAGGCTCCGC

CCCCCTGACGAGCATCACAAAAATCGACGCTCAAGTCAGAGGTGGCGAAACCCGACAGGACTATAAAGAT

ACCAGGCGTTTCCCCCTGGAAGCTCCCTCGTGCGCTCTCCTGTTCCGACCCTGCCGCTTACCGGATACCT

GTCCGCCTTTCTCCCTTCGGGAAGCGTGGCGCTTTCTCAATGCTCACGCTGTAGGTATCTCAGTTCGGTG

TAGGTCGTTCGCTCCAAGCTGGGCTGTGTGCACGAACCCCCCGTTCAGCCCGACCGCTGCGCCTTATCCG

GTAACTATCGTCTTGAGTCCAACCCGGTAAGACACGACTTATCGCCACTGGCAGCAGCCACTGGTAACAG

GATTAGCAGAGCGAGGTATGTAGGCGGTGCTACAGAGTTCTTGAAGTGGTGGCCTAACTACGGCTACACT

AGAAGGACAGTATTTGGTATCTGCGCTCTGCTGAAGCCAGTTACCTTCGGAAAAAGAGTTGGTAGCTCTT

GATCCGGCAAACAAACCACCGCTGGTAGCGGTGGTTTTTTTGTTTGCAAGCAGCAGATTACGCGCAGAAA

AAAAGGATATCAAGAAGATCCTTTGATCTTTTCTACGGGGTCTGACGCTCAGTGGAACGAAAACTCACGT

TAAGGGATTTTGGTCATGAGATTATCAAAAAGGATCTTCACCTAGATCCTTTTAAATTAAAAATGAAGTT

TTAAATCAATCTAAAGTATATATGAGTAAACTTGGTCTGACAGTTACCAATGCTTAATCAGTGAGGCACC

TATCTCAGCGATCTGTCTATTTCGTTCATCCATAGTTGCCTGACTCCCCGTCGTGTAGATAACTACGATA

CGGGAGGGCTTACCATCTGGCCCCAGTGCTGCAATGATACCGCGAGACCCACGCTCACCGGCTCCAGATT

TATCAGCAATAAACCAGCCAGCCGGAAGGGCCGAGCGCAGAAGTGGTCCTGCAACTTTATCCGCCTCCAT

CCAGTCTATTAAACAAGTGGCAGCAACGGATTCGCAAACCTGTCACGCCTTTTGTGCCAAAAGCCGCGCC

AGGTTTGCGATCCGCTGTGCCAGGCGTTAGGCGTCATATGAAGATTTCGGTGATCCCTGAGCAGGTGGCG

GAAACATTGGATGCTGAGAACCATTTCATTGTTCGTGAAGTGTTCGATGTGCACCTATCCGACCAAGGCT

TTGAACTATCTACCAGAAGTGTGAGCCCCTACCGGAAGGATTACATCTCGGATGATGACTCTGATGAAGA

CTCTGCTTGCTATGGCGCATTCATCGACCAAGAGCTTGTCGGGAAGATTGAACTCAACTCAACATGGAAC

GATCTAGCCTCTATCGAACACATTGTTGTGTCGCACACGCACCGAGGCAAAGGAGTCGCGCACAGTCTCA

TCGAATTTGCGAAAAAGTGGGCACTAAGCAGACAGCTCCTTGGCATACGATTAGAGACACAAACGAACAA

TGTACCTGCCTGCAATTTGTACGCAAAATGTGGCTTTACTCTCGGCGGCATTGACCTGTTCACGTATAAA

ACTAGACCTCAAGTCTCGAACGAAACAGCGATGTACTGGTACTGGTTCTCGGGAGCACAGGATGACGCCT

AACAATTCATTCAAGCCGACACCGCTTCGCGGCGCGGCTTAATTCAGGAGTTAAACATCATGAGGGAAGC

GGTGATCGCCGAAGTATCGACTCAACTATCAGAGGTAGTTGGCGTCATCGAGCGCCATCTCGAACCGACG

TTGCTGGCCGTACATTTGTACGGCTCCGCAGTGGATGGCGGCCTGAAGCCACACAGTGATATTGATTTGC

TGGTTACGGTGACCGTAAGGCTTGATGAAACAACGCGGCGAGCTTTGATCAACGACCTTTTGGAAACTTC

GGCTTCCCCTGGAGAGAGCGAGATTCTCCGCGCTGTAGAAGTCACCATTGTTGTGCACGACGACATCATT

CCGTGGCGTTATCCAGCTAAGCGCGAACTGCAATTTGGAGAATGGCAGCGCAATGACATTCTTGCAGGTA

TCTTCGAGCCAGCCACGATCGACATTGATCTGGCTATCTTGCTGACAAAAGCAAGAGAACATAGCGTTGC

CTTGGTAGGTCCAGCGGCGGAGGAACTCTTTGATCCGGTTCCTGAACAGGATCTATTTGAGGCGCTAAAT

GAAACCTTAACGCTATGGAACTCGCCGCCCGACTGGGCTGGCGATGAGCGAAATGTAGTGCTTACGTTGT

CCCGCATTTGGTACAGCGCAGTAACCGGCAAAATCGCGCCGAAGGATGTCGCTGCCGACTGGGCAATGGA

GCGCCTGCCGGCCCAGTATCAGCCCGTCATACTTGAAGCTAGGCAGGCTTATCTTGGACAAGAAGATCGC

TTGGCCTCGCGCGCAGATCAGTTGGAAGAATTTGTTCACTACGTGAAAGGCGAGATCACCAAGGTAGTCG

GCAAATAATGTCTAACAATTCGTTCAAGCCGACGCCGCTTCGCGGCGCGGCTTAACTCAAGCGTTAGAGA

GCTGGGGAAGACTATGCGCGATCTGTTGAAGGTGGTTCTAAGCCTCGTACTTGCGATGGCATCGGGGCAG

GCACTTGCTGACCTGCCAATTGTTTTAGTGGATGAAGCTCGTCTTCCCTATGACTACTCCCCATCCAACT

ACGACATTTCTCCAAGCAACTACGACAACTCCATAAGCAATTACGACAATAGTCCATCAAATTACGACAA

CTCTGAGAGCAACTACGATAATAGTTCATCCAATTACGACAATAGTCGCAACGGAAATCGTAGGCTTATA

TATAGCGCAAATGGGTCTCGCACTTTCGCCGGCTACTACGTCATTGCCAACAATGGGACAACGAACTTCT

TTTCCACATCTGGCAAAAGGATGTTCTACACCCCAAAAGGGGGGCGCGGCGTCTATGGCGGCAAAGATGG

GAGCTTCTGCGGGGCATTGGTCGTCATAAATGGCCAATTTTCGCTTGCCCTGACAGATAACGGCCTGAAG

ATCATGTATCTAAGCAACTAGCCTGCTCTCTAATAAAATGTTAGGAGCTTGGCTGCCATTTTTGGGGTGA

GGCCGTTCGCGGCCGAGGGGCGCAGCCCCTGGGGGGATGGGAGGCCCGCGTTAGCGGGCCGGGAGGGTTC

GAGAAGGGGGGGCACCCCCCTTCGGCGTGCGCGGTCACGCGCCAGGGCGCAGCCCTGGTTAAAAACAAGG

TTTATAAATATTGGTTTAAAAGCAGGTTAAAAGACAGGTTAGCGGTGGCCGAAAAACGGGCGGAAACCCT

TGCAAATGCTGGATTTTCTGCCTGTGGACAGCCCCTCAAATGTCAATAGGTGCGCCCCTCATCTGTCAGC

ACTCTGCCCCTCAAGTGTCAAGGATCGCGCCCCTCATCTGTCAGTAGTCGCGCCCCTCAAGTGTCAATAC

CGCAGGGCACTTATCCCCAGGCTTGTCCACATCATCTGTGGGAAACTCGCGTAAAATCAGGCGTTTTCGC

CGATTTGCGAGGCTGGCCAGCTCCACGTCGCCGGCCGAAATCGAGCCTGCCCCTCATCTGTCAACGCCGC

GCCGGGTGAGTCGGCCCCTCAAGTGTCAACGTCCGCCCCTCATCTGTCAGTGAGGGCCAAGTTTTCCGCG

AGGTATCCACAACGCCGGCGGCCGGCCGCGGTGTCTCGCACACGGCTTCGACGGCGTTTCTGGCGCGTTT

GCAGGGCCATAGACGGCCGCCAGCCCAGCGGCGAGGGCAACCAGCCCGGTGAGCGTCGGAAAGGGTCGAC

ATCTTGCTGCGTTCGGATATTTTCGTGGAGTTCCCGCCACAGACCCGGATTGAAGGCGAGATCCAGCAAC

TCGCGCCAGATCATCCTGTGACGGAACTTTGGCGCGTGATGACTGGCCAGGACGTCGGCCGAAAGAGCGA

CAAGCAGATCACGATTTTCGACAGCGTCGGATTTGCGATCGAGGATTTTTCGGCGCTGCGCTACGTCCGC

GACCGCGTTGAGGGATCAAGCCACAGCAGCCCACTCGACCTTCTAGCCGACCCAGACGAGCCAAGGGATC

TTTTTGGAATGCTGCTCCGTCGTCAGGCTTTCCGACGTTTGGGTGGTTGAACAGAAGTCATTATCGTACG

GAATGCCAGCACTCCCGAGGGGAACCCTGTGGTTGGCATGCACATACAAATGGACGAACGGATAAACCTT

TTCACGCCCTTTTAAATATCCGTTATTCTAATAAACGCTCTTTTCTCTTAGGTTTACCCGCCAATATATC

CTGTCAAACACTGATAGTTTAAACTGAAGGCGGGAAACGACAATCTGATCATGAGCGGAGAATTAAGGGA

GTCACGTTATGACCCCCGCCGATGACGCGGGACAAGCCGTTTTACGTTTGGAACTGACAGAACCGCAACG

ATTGAAGGAGCCACTCAGCCCCAATACGCAAACCGCCTCTCCCCGCGCGTTGGCCGATTCATTAATGCAG

CTGGCACGACAGGTTTCCCGACTGGAAAGCGGGCAGTGAGCGCAACGCAATTAATGTGAGTTAGCTCACT

CATTAGGCACCCCAGGCTTTACACTTTATGCTTCCGGCTCGTATGTTGTGTGGAATTGTGAGCGGATAAC

AATTTCACACAGGAAACAGCTATGACCATGATTACGCCAAGCTATTTAGGTGACACTATAGAATACTCAA

GCTATGCATCCAACGCGTTGGGAGCTCTCCCATATCGACCTGCAGGCGGCCGCTCGACGAATTAATTCCA

ATCCCACAAAAATCTGAGCTTAACAGCACAGTTGCTCCTCTCAGAGCAGAATCGGGTATTCAACACCCTC

ATATCAACTACTACGTTGTGTATAACGGTCCACATGCCGGTATATACGATGACTGGGGTTGTACAAAGGC

GGCAACAAACGGCGTTCCCGGAGTTGCACACAAGAAATTTGCCACTATTACAGAGGCAAGAGCAGCAGCT

GACGCGTACACAACAAGTCAGCAAACAGACAGGTTGAACTTCATCCCCAAAGGAGAAGCTCAACTCAAGC

CCAAGAGCTTTGCTAAGGCCCTAACAAGCCCACCAAAGCAAAAAGCCCACTGGCTCACGCTAGGAACCAA

AAGGCCCAGCAGTGATCCAGCCCCAAAAGAGATCTCCTTTGCCCCGGAGATTACAATGGACGATTTCCTC

TATCTTTACGATCTAGGAAGGAAGTTCGAAGGTGAAGGTGACGACACTATGTTCACCACTGATAATGAGA

AGGTTAGCCTCTTCAATTTCAGAAAGAATGCTGACCCACAGATGGTTAGAGAGGCCTACGCAGCAGGTCT

CATCAAGACGATCTACCCGAGTAACAATCTCCAGGAGATCAAATACCTTCCCAAGAAGGTTAAAGATGCA

GTCAAAAGATTCAGGACTAATTGCATCAAGAACACAGAGAAAGACATATTTCTCAAGATCAGAAGTACTA

TTCCAGTATGGACGATTCAAGGCTTGCTTCATAAACCAAGGCAAGTAATAGAGATTGGAGTCTCTAAAAA

GGTAGTTCCTACTGAATCTAAGGCCATGCATGGAGTCTAAGATTCAAATCGAGGATCTAACAGAACTCGC

CGTGAAGACTGGCGAACAGTTCATACAGAGTCTTTTACGACTCAATGACAAGAAGAAAATCTTCGTCAAC

ATGGTGGAGCACGACACTCTGGTCTACTCCAAAAATGTCAAAGATACAGTCTCAGAAGACCAAAGGGCTA

TTGAGACTTTTCAACAAAGGATAATTTCGGGAAACCTCCTCGGATTCCATTGCCCAGCTATCTGTCACTT

CATCGAAAGGACAGTAGAAAAGGAAGGTGGCTCCTACAAATGCCATCATTGCGATAAAGGAAAGGCTATC

ATTCAAGATCTCTCTGCCGACAGTGGTCCCAAAGATGGACCCCCACCCACGAGGAGCATCGTGGAAAAAG

AAGACGTTCCAACCACGTCTTCAAAGCAAGTGGATTGATGTGACATCTCCACTGACGTAAGGGATGACGC

ACAATCCCACTATCCTTCGCAAGACCCTTCCTCTATATAAGGAAGTTCATTTCATTTGGAGAGGACACGC

TCGACTATTTTTACAACAATTACC**ATGAAGACTAATCTTTTTCTCTTTCTCATCTTTTCACTTCTCCTAT**

**CATTATCCTCGGCCGAATTCCTCGAG**TCAC**GACATACAGGCCCATGTACCGA***ACAGAATGTACCAAAGCC*

*CTGA***TCTAGA**AGTAAAGGAGAAGAACTTTTCACTGGAGTTGTCCCAATTCTTGTTGAATTAGATGGTGAT

GTTAATGGGTACAAATTTTCTGTCAGTGGAGAGGGTGAAGGTGATGCAACATACGGAAAACTTACCCTTA

AATTTATTTGCACTACTGGAAAACTACCTGTTCCATGGCCAACACTTGTCACTACTTTCTCTTATGGTGT

TCAATGCTTTTCAAGATACCCAGATCATATGAAGCGGCACGACTTCTTCAAGAGCGCCATGCCTGAGGGA

TACGTGCAGGAGAGGACCATCTTCTTCAAGGACGACGGGAACTACAAGACACGTGCTGAAGTCAAGTTTG

AGGGAGACACCCTCGTCAACAGGATCGAGCTTAAGGGAATCGATTTCAAGGAGGACGGAAACATCCTCGG

CCACAAGTTGGAATACAACTACAACTCCCACAACGTATACATCATGGCCGACAAGCAAAAGAACGGCATC

AAAGCCAACTTCAAGACCCGCCACAACATCGAAGACGGCGGCGTGCAACTCGCTGATCATTATCAACAAA

ATACTCCAATTGGCGATGGCCCTGTCCTTTTACCAGACAACCATTACCTGTCCACACAATCTGCCCTTTC

GAAAGATCCCAACGAAAAGAGAGACCACATGGTCCTTCTTGAGTTTGTAACAGCTGCTGGGATTACACAT

GGCATGGATGAACTATACAAACATGATGAGCTTTGAACTAGAGTCCTGCTTTAATGAGATATGCGAGACG

CCTATGATCGCATGATATTTGCTTTCAATTCTGTTGTGCACGTTGTAAAAAACCTGAGCATGTGTAGCTC

AGATCCTTACCGCCGGTTTCGGTTCATTCTAATGAATATATCACCCGTTACTATCGTATTTTTATGAATA

ATATTCTCCGTTCAATTTACTGATTGTACCCTACTACTTATATGTACAATATTAAAATGAAAACAATATA

TTGTGCTGAATAGGTTTATAGCGACATCTATGATAGAGCGCCACAATAACAAACAATTGCGTTTTATTAT

TACAAATCCAATTTTAAAAAAAGCGGCAGAACCGGTCAAACCTAAAAGACTGATTACATAAATCTTATTC

AAATTTCAAAAGGCCCCAGGGGCTAGTATCTACGACACACCGAGCGGCGAACTAATAACGTTCACTGAAG

GGAACTCCGGTTCCCCGCCGGCGCGCATGGGTGAGATTCCTTGAAGTTGAGTATTGGCCGTCCGCTCTAC

CGAAAGTTACGGGCACCATTCAACCCGGTCCAGCACGGCGGCCGGGTAACCGACTTGCTGCCCCGAGAAT

TATGCAGCATTTTTTTGGTGTATGTGGGCCCCAAATGAAGTGCAGGTCAAACCTTGACAGTGACGACAAA

TCGTTGGGCGGGTCCAGGGCGAATTTTGCGACAACATGTCGAGGCTCAGCAGGACCTGCAGGCATGCAAG

CTAGCTTACTAGTGATGCATATTCTATAGTGTCACCTAAATCTGC

>pMS4-AMIRV2 (**AK0067 (1bp - 14675bp, direct) 14675bp**)

GGCCGCACTAGTGATATCCCGCGGCCATGGCGGCCGGGAGCATGCGACGTCGGGCCCAATTCGCCCTATA

GTGAGTCGTATTACAATTCACTGGCCGTCGTTTTACAACGTCGTGACTGGGAAAACCCTGGCGTTACCCA

ACTTAATCGCCTTGCAGCACATCCCCCTTTCGCCAGCTGGCGTAATAGCGAAGAGGCCCGCACCGATCGC

CCTTCCCAACAGTTGCGCAGCCTGAATGGCGAATGGAAATTGTAAACGTTAATGGGTTTCTGGAGTTTAA

TGAGCTAAGCACATACGTCAGAAACCATTATTGCGCGTTCAAAAGTCGCCTAAGGTCACTATCAGCTAGC

AAATATTTCTTGTCAAAAATGCTCCACTGACGTTCCATAAATTCCCCTCGGTATCCAATTAGAGTCTCAT

ATTCACTCTCAATCCAAATAATCTGCAATGGCAATTACCTTATCCGCAACTTCTTTACCTATTTCCGCCC

GGATCCGGGCAGGTTCTCCGGCCGCTTGGGTGGAGAGGCTATTCGGCTATGACTGGGCACAACAGACAAT

CGGCTGCTCTGATGCCGCCGTGTTCCGGCTGTCAGCGCAGGGGCGCCCGGTTCTTTTTGTCAAGACCGAC

CTGTCCGGTGCCCTGAATGAACTGCAGGACGAGGCAGCGCGGCTATCGTGGCTGGCCACGACGGGCGTTC

CTTGCGCAGCTGTGCTCGACGTTGTCACTGAAGCGGGAAGGGACTGGCTGCTATTGGGCGAAGTGCCGGG

GCAGGATCTCCTGTCATCTCACCTTGCTCCTGCCGAGAAAGTATCCATCATGGCTGATGCAATGCGGCGG

CTGCATACGCTTGATCCGGCTACCTGCCCATTCGACCACCAAGCGAAACATCGCATCGAGCGAGCACGTA

CTCGGATGGAAGCCGGTCTTGTCGATCAGGATGATCTGGACGAAGAGCATCAGGGGCTCGCGCCAGCCGA

ACTGTTCGCCAGGCTCAAGGCGCGCATGCCCGACGGCGAGGATCTCGTCGTGACCCATGGCGATGCCTGC

TTGCCGAATATCATGGTGGAAAATGGCCGCTTTTCTGGATTCATCGACTGTGGCCGGCTGGGTGTGGCGG

ACCGCTATCAGGACATAGCGTTGGCTACCCGTGATATTGCTGAAGAGCTTGGCGGCGAATGGGCTGACCG

CTTCCTCGTGCTTTACGGTATCGCCGCTCCCGATTCGCAGCGCATCGCCTTCTATCGCCTTCTTGACGAG

TTCTTCTGAGCGGGACTCTGGGGTTCGAAATGACCGACCAAGCGACGCCCAACCTGCCATCACGAGATTT

CGATTCCACCGCCGCCTTCTATGAAAGGTTGGGCTTCGGAATCGTTTTCCGGGACGCCGGCTGGATGATC

CTCCAGCGCGGGGATCTCATGCTGGAGTTCTTCGCCCACCCCGATCCAACACTTACGTTTGCAACGTCCA

AGAGCAAATAGACCACGAACGCCGGAAGGTTGCCGCAGCGTGTGGATTGCGTCTCAATTCTCTCTTGCAG

GAATGCAATGATGAATATGATACTGACTATGAAACTTTGAGGGAATACTGCCTAGCACCGTCACCTCATA

ACGTGCATCATGCATGCCCTGACAACATGGAACATCGCTATTTTTCTGAAGAATTATGCTCGTTGGAGGA

TGTCGCGGCAATTGCAGCTATTGCCAACATCGAACTACCCCTCACGCATGCATTCATCAATATTATTCAT

GCGGGGAAAGGCAAGATTAATCCAACTGGCAAATCATCCAGCGTGATTGGTAACTTCAGTTCCAGCGACT

TGATTCGTTTTGGTGCTACCCACGTTTTCAATAAGGACGAGATGGTGGAGTAAAGAAGGAGTGCGTCGAA

GCAGATCGTTCAAACATTTGGCAATAAAGTTTCTTAAGATTGAATCCTGTTGCCGGTCTTGCGATGATTA

TCATATAATTTCTGTTGAATTACGTTAAGCATGTAATAATTAACATGTAATGCATGACGTTATTTATGAG

ATGGGTTTTTATGATTAGAGTCCCGCAATTATACATTTAATACGCGATAGAAAACAAAATATAGCGCGCA

AACTAGGATAAATTATCGCGCGCGGTGTCATCTATGTTACTAGATCGAATTAATTCCAGGCGGTGAAGGG

CAATCAGCTGTTGCCCGTCTCACTGGTGAAAAGAAAAACCACCCCAGTACATTAAAAACGTCCGCAATGT

GTTATTAAGTTGTCTAAGCGTCAATTTGTTTACACCACAATATATCCTGCCACCAGCCAGCCAACAGCTC

CCCGACCGGCAGCTCGGCACAAAATCACCACTCGATACAGGCAGCCCATCAGTCCGGGACGGCGTCAGCG

GGAGAGCCGTTGTAAGGCGGCAGACTTTGCTCATGTTACCGATGCTATTCGGAAGAACGGCAACTAAGCT

GCCGGGTTTGAAACACGGATGATCTCGCGGAGGGTAGCATGTTGATTGTAACGATGACAGAGCGTTGCTG

CCTGTGATCAAATATCATCTCCCTCGCAGAGATCCGAATTATCAGCCTTCTTATTCATTTCTCGCTTAAC

CGTGACAGGCTGTCGATCTTGAGAACTATGCCGACATAATAGGAAATCGCTGGATAAAGCCGCTGAGGAA

GCTGAGTGGCGCTATTTCTTTAGAAGTGAACGTTGACGATGTCGACGGATCTTTTCCGCTGCATAACCCT

GCTTCGGGGTCATTATAGCGATTTTTTCGGTATATCCATCCTTTTTCGCACGATATACAGGATTTTGCCA

AAGGGTTCGTGTAGACTTTCCTTGGTGTATCCAACGGCGTCAGCCGGGCAGGATAGGTGAAGTAGGCCCA

CCCGCGAGCGGGTGTTCCTTCTTCACTGTCCCTTATTCGCACCTGGCGGTGCTCAACGGGAATCCTGCTC

TGCGAGGCTGGCCGGCTACCGCCGGCGTAACAGATGAGGGCAAGCGGATGGCTGATGAAACCAAGCCAAC

CAGGGGTGATGCTGCCAACTTACTGATTTAGTGTATGATGGTGTTTTTGAGGTGCTCCAGTGGCTTCTGT

TTCTATCAGCTGTCCCTCCTGTTCAGCTACTGACGGGGTGGTGCGTAACGGCAAAAGCACCGCCGGACAT

CAGCGCTATCTCTGCTCTCACTGCCGTAAAACATGGCAACTGCAGTTCACTTACACCGCTTCTCAACCCG

GTACGCACCAGAAAATCATTGATATGGCCATGAATGGCGTTGGATGCCGGGCAACAGCCCGCATTATGGG

CGTTGGCCTCAACACGATTTTACGTCACTTAAAAAACTCAGGCCGCAGTCGGTAACCTCGCGCATACAGC

CGGGCAGTGACGTCATCGTCTGCGCGGAAATGGACGAACAGTGGGGCTATGTCGGGGCTAAATCGCGCCA

GCGCTGGCTGTTTTACGCGTATGACAGTCTCCGGAAGACGGTTGTTGCGCACGTATTCGGTGAACGCACT

ATGGCGACGCTGGGGCGTCTTATGAGCCTGCTGTCACCCTTTGACGTGGTGATATGGATGACGGATGGCT

GGCCGCTGTATGAATCCCGCCTGAAGGGAAAGCTGCACGTAATCAGCAAGCGATATACGCAGCGAATTGA

GCGGCATAACCTGAATCTGAGGCAGCACCTGGCACGGCTGGGACGGAAGTCGCTGTCGTTCTCAAAATCG

GTGGAGCTGCATGACAAAGTCATCGGGCATTATCTGAACATAAAACACTATCAATAAGTTGGAGTCATTA

CCCAACCAGGAAGGGCAGCCCACCTATCAAGGTGTACTGCCTTCCAGACGAACGAAGAGCGATTGAGGAA

AAGGCGGCGGCGGCCGGCATGAGCCTGTCGGCCTACCTGCTGGCCGTCGGCCAGGGCTACAAAATCACGG

GCGTCGTGGACTATGAGCACGTCCGCGAGCTGGCCCGCATCAATGGCGACCTGGGCCGCCTGGGCGGCCT

GCTGAAACTCTGGCTCACCGACGACCCGCGCACGGCGCGGTTCGGTGATGCCACGATCCTCGCCCTGCTG

GCGAAGATCGAAGAGAAGCAGGACGAGCTTGGCAAGGTCATGATGGGCGTGGTCCGCCCGAGGGCAGAGC

CATGACTTTTTTAGCCGCTAAAACGGCCGGGGGGTGCGCGTGATTGCCAAGCACGTCCCCATGCGCTCCA

TCAAGAAGAGCGACTTCGCGGAGCTGGTATTCGTGCAGGGCAAGATTCGGAATACCAAGTACGAGAAGGA

CGGCCAGACGGTCTACGGGACCGACTTCATTGCCGATAAGGTGGATTATCTGGACACCAAGGCACCAGGC

GGGTCAAATCAGGAATAAGGGCACATTGCCCCGGCGTGAGTCGGGGCAATCCCGCAAGGAGGGTGAATGA

ATCGGACGTTTGACCGGAAGGCATACAGGCAAGAACTGATCGACGCGGGGTTTTCCGCCGAGGATGCCGA

AACCATCGCAAGCCGCACCGTCATGCGTGCGCCCCGCGAAACCTTCCAGTCCGTCGGCTCGATGGTCCAG

CAAGCTACGGCCAAGATCGAGCGCGACAGCGTGCAACTGGCTCCCCCTGCCCTGCCCGCGCCATCGGCCG

CCGTGGAGCGTTCGCGTCGTCTCGAACAGGAGGCGGCAGGTTTGGCGAAGTCGATGACCATCGACACGCG

AGGAACTATGACGACCAAGAAGCGAAAAACCGCCGGCGAGGACCTGGCAAAACAGGTCAGCGAGGCCAAG

CAGGCCGCGTTGCTGAAACACACGAAGCAGCAGATCAAGGAAATGCAGCTTTCCTTGTTCGATATTGCGC

CGTGGCCGGACACGATGCGAGCGATGCCAAACGACACGGCCCGCTCTGCCCTGTTCACCACGCGCAACAA

GAAAATCCCGCGCGAGGCGCTGCAAAACAAGGTCATTTTCCACGTCAACAAGGACGTGAAGATCACCTAC

ACCGGCGTCGAGCTGCGGGCCGACGATGACGAACTGGTGTGGCAGCAGGTGTTGGAGTACGCGAAGCGCA

CCCCTATCGGCGAGCCGATCACCTTCACGTTCTACGAGCTTTGCCAGGACCTGGGCTGGTCGATCAATGG

CCGGTATTACACGAAGGCCGAGGAATGCCTGTCGCGCCTACAGGCGACGGCGATGGGCTTCACGTCCGAC

CGCGTTGGGCACCTGGAATCGGTGTCGCTGCTGCACCGCTTCCGCGTCCTGGACCGTGGCAAGAAAACGT

CCCGTTGCCAGGTCCTGATCGACGAGGAAATCGTCGTGCTGTTTGCTGGCGACCACTACACGAAATTCAT

ATGGGAGAAGTACCGCAAGCTGTCGCCGACGGCCCGACGGATGTTCGACTATTTCAGCTCGCACCGGGAG

CCGTACCCGCTCAAGCTGGAAACCTTCCGCCTCATGTGCGGATCGGATTCCACCCGCGTGAAGAAGTGGC

GCGAGCAGGTCGGCGAAGCCTGCGAAGAGTTGCGAGGCAGCGGCCTGGTGGAACACGCCTGGGTCAATGA

TGACCTGGTGCATTGCAAACGCTAGGGCCTTGTGGGGTCAGTTCCGGCTGGGGGTTCAGCAGCCAGCGCT

TTACTGGCATTTCAGGAACAAGCGGGCACTGCTCGACGCACTTGCTTCGCTCAGTATCGCTCGGGACGCA

CGGCGCGCTCTACGAACTGCCGATAAACAGAGGATTAAAATTGACAATTGTGATTAAGGCTCAGATTCGA

CGGCTTGGAGCGGCCGACGTGCAGGATTTCCGCGAGATCCGATTGTCGGCCCTGAAGAAAGCTCCAGAGA

TGTTCGGGTCCGTTTACGAGCACGAGGAGAAAAAGCCCATGGAGGCGTTCGCTGAACGGTTGCGAGATGC

CGTGGCATTCGGCGCCTACATCGACGGCGAGATCATTGGGCTGTCGGTCTTCAAACAGGAGGACGGCCCC

AAGGACGCTCACAAGGCGCATCTGTCCGGCGTTTTCGTGGAGCCCGAACAGCGAGGCCGAGGGGTCGCCG

GTATGCTGCTGCGGGCGTTGCCGGCGGGTTTATTGCTCGTGATGATCGTCCGACAGATTCCAACGGGAAT

CTGGTGGATGCGCATCTTCATCCTCGGCGCACTTAATATTTCGCTATTCTGGAGCTTGTTGTTTATTTCG

GTCTACCGCCTGCCGGGCGGGGTCGCGGCGACGGTAGGCGCTGTGCAGCCGCTGATGGTCGTGTTCATCT

CTGCCGCTCTGCTAGGTAGCCCGATACGATTGATGGCGGTCCTGGGGGCTATTTGCGGAACTGCGGGCGT

GGCGCTGTTGGTGTTGACACCAAACGCAGCGCTAGATCCTGTCGGCGTCGCAGCGGGCCTGGCGGGGGCG

GTTTCCATGGCGTTCGGAACCGTGCTGACCCGCAAGTGGCAACCTCCCGTGCCTCTGCTCACCTTTACCG

CCTGGCAACTGGCGGCCGGAGGACTTCTGCTCGTTCCAGTAGCTTTAGTGTTTGATCCGCCAATCCCGAT

GCCTACAGGAACCAATGTTCTCGGCCTGGCGTGGCTCGGCCTGATCGGAGCGGGTTTAACCTACTTCCTT

TGGTTCCGGGGGATCTCGCGACTCGAACCTACAGTTGTTTCCTTACTGGGCTTTCTCAGCCGGGATGGCG

CTAAGAAGCTATTGCCGCCGATCTTCATATGCGGTGTGAAATACCGCACAGATGCGTAAGGAGAAAATAC

CGCATCAGGCGCTCTTCCGCTTCCTCGCTCACTGACTCGCTGCGCTCGGTCGTTCGGCTGCGGCGAGCGG

TATCAGCTCACTCAAAGGCGGTAATACGGTTATCCACAGAATCAGGGGATAACGCAGGAAAGAACATGTG

AGCAAAAGGCCAGCAAAAGGCCAGGAACCGTAAAAAGGCCGCGTTGCTGGCGTTTTTCCATAGGCTCCGC

CCCCCTGACGAGCATCACAAAAATCGACGCTCAAGTCAGAGGTGGCGAAACCCGACAGGACTATAAAGAT

ACCAGGCGTTTCCCCCTGGAAGCTCCCTCGTGCGCTCTCCTGTTCCGACCCTGCCGCTTACCGGATACCT

GTCCGCCTTTCTCCCTTCGGGAAGCGTGGCGCTTTCTCAATGCTCACGCTGTAGGTATCTCAGTTCGGTG

TAGGTCGTTCGCTCCAAGCTGGGCTGTGTGCACGAACCCCCCGTTCAGCCCGACCGCTGCGCCTTATCCG

GTAACTATCGTCTTGAGTCCAACCCGGTAAGACACGACTTATCGCCACTGGCAGCAGCCACTGGTAACAG

GATTAGCAGAGCGAGGTATGTAGGCGGTGCTACAGAGTTCTTGAAGTGGTGGCCTAACTACGGCTACACT

AGAAGGACAGTATTTGGTATCTGCGCTCTGCTGAAGCCAGTTACCTTCGGAAAAAGAGTTGGTAGCTCTT

GATCCGGCAAACAAACCACCGCTGGTAGCGGTGGTTTTTTTGTTTGCAAGCAGCAGATTACGCGCAGAAA

AAAAGGATATCAAGAAGATCCTTTGATCTTTTCTACGGGGTCTGACGCTCAGTGGAACGAAAACTCACGT

TAAGGGATTTTGGTCATGAGATTATCAAAAAGGATCTTCACCTAGATCCTTTTAAATTAAAAATGAAGTT

TTAAATCAATCTAAAGTATATATGAGTAAACTTGGTCTGACAGTTACCAATGCTTAATCAGTGAGGCACC

TATCTCAGCGATCTGTCTATTTCGTTCATCCATAGTTGCCTGACTCCCCGTCGTGTAGATAACTACGATA

CGGGAGGGCTTACCATCTGGCCCCAGTGCTGCAATGATACCGCGAGACCCACGCTCACCGGCTCCAGATT

TATCAGCAATAAACCAGCCAGCCGGAAGGGCCGAGCGCAGAAGTGGTCCTGCAACTTTATCCGCCTCCAT

CCAGTCTATTAAACAAGTGGCAGCAACGGATTCGCAAACCTGTCACGCCTTTTGTGCCAAAAGCCGCGCC

AGGTTTGCGATCCGCTGTGCCAGGCGTTAGGCGTCATATGAAGATTTCGGTGATCCCTGAGCAGGTGGCG

GAAACATTGGATGCTGAGAACCATTTCATTGTTCGTGAAGTGTTCGATGTGCACCTATCCGACCAAGGCT

TTGAACTATCTACCAGAAGTGTGAGCCCCTACCGGAAGGATTACATCTCGGATGATGACTCTGATGAAGA

CTCTGCTTGCTATGGCGCATTCATCGACCAAGAGCTTGTCGGGAAGATTGAACTCAACTCAACATGGAAC

GATCTAGCCTCTATCGAACACATTGTTGTGTCGCACACGCACCGAGGCAAAGGAGTCGCGCACAGTCTCA

TCGAATTTGCGAAAAAGTGGGCACTAAGCAGACAGCTCCTTGGCATACGATTAGAGACACAAACGAACAA

TGTACCTGCCTGCAATTTGTACGCAAAATGTGGCTTTACTCTCGGCGGCATTGACCTGTTCACGTATAAA

ACTAGACCTCAAGTCTCGAACGAAACAGCGATGTACTGGTACTGGTTCTCGGGAGCACAGGATGACGCCT

AACAATTCATTCAAGCCGACACCGCTTCGCGGCGCGGCTTAATTCAGGAGTTAAACATCATGAGGGAAGC

GGTGATCGCCGAAGTATCGACTCAACTATCAGAGGTAGTTGGCGTCATCGAGCGCCATCTCGAACCGACG

TTGCTGGCCGTACATTTGTACGGCTCCGCAGTGGATGGCGGCCTGAAGCCACACAGTGATATTGATTTGC

TGGTTACGGTGACCGTAAGGCTTGATGAAACAACGCGGCGAGCTTTGATCAACGACCTTTTGGAAACTTC

GGCTTCCCCTGGAGAGAGCGAGATTCTCCGCGCTGTAGAAGTCACCATTGTTGTGCACGACGACATCATT

CCGTGGCGTTATCCAGCTAAGCGCGAACTGCAATTTGGAGAATGGCAGCGCAATGACATTCTTGCAGGTA

TCTTCGAGCCAGCCACGATCGACATTGATCTGGCTATCTTGCTGACAAAAGCAAGAGAACATAGCGTTGC

CTTGGTAGGTCCAGCGGCGGAGGAACTCTTTGATCCGGTTCCTGAACAGGATCTATTTGAGGCGCTAAAT

GAAACCTTAACGCTATGGAACTCGCCGCCCGACTGGGCTGGCGATGAGCGAAATGTAGTGCTTACGTTGT

CCCGCATTTGGTACAGCGCAGTAACCGGCAAAATCGCGCCGAAGGATGTCGCTGCCGACTGGGCAATGGA

GCGCCTGCCGGCCCAGTATCAGCCCGTCATACTTGAAGCTAGGCAGGCTTATCTTGGACAAGAAGATCGC

TTGGCCTCGCGCGCAGATCAGTTGGAAGAATTTGTTCACTACGTGAAAGGCGAGATCACCAAGGTAGTCG

GCAAATAATGTCTAACAATTCGTTCAAGCCGACGCCGCTTCGCGGCGCGGCTTAACTCAAGCGTTAGAGA

GCTGGGGAAGACTATGCGCGATCTGTTGAAGGTGGTTCTAAGCCTCGTACTTGCGATGGCATCGGGGCAG

GCACTTGCTGACCTGCCAATTGTTTTAGTGGATGAAGCTCGTCTTCCCTATGACTACTCCCCATCCAACT

ACGACATTTCTCCAAGCAACTACGACAACTCCATAAGCAATTACGACAATAGTCCATCAAATTACGACAA

CTCTGAGAGCAACTACGATAATAGTTCATCCAATTACGACAATAGTCGCAACGGAAATCGTAGGCTTATA

TATAGCGCAAATGGGTCTCGCACTTTCGCCGGCTACTACGTCATTGCCAACAATGGGACAACGAACTTCT

TTTCCACATCTGGCAAAAGGATGTTCTACACCCCAAAAGGGGGGCGCGGCGTCTATGGCGGCAAAGATGG

GAGCTTCTGCGGGGCATTGGTCGTCATAAATGGCCAATTTTCGCTTGCCCTGACAGATAACGGCCTGAAG

ATCATGTATCTAAGCAACTAGCCTGCTCTCTAATAAAATGTTAGGAGCTTGGCTGCCATTTTTGGGGTGA

GGCCGTTCGCGGCCGAGGGGCGCAGCCCCTGGGGGGATGGGAGGCCCGCGTTAGCGGGCCGGGAGGGTTC

GAGAAGGGGGGGCACCCCCCTTCGGCGTGCGCGGTCACGCGCCAGGGCGCAGCCCTGGTTAAAAACAAGG

TTTATAAATATTGGTTTAAAAGCAGGTTAAAAGACAGGTTAGCGGTGGCCGAAAAACGGGCGGAAACCCT

TGCAAATGCTGGATTTTCTGCCTGTGGACAGCCCCTCAAATGTCAATAGGTGCGCCCCTCATCTGTCAGC

ACTCTGCCCCTCAAGTGTCAAGGATCGCGCCCCTCATCTGTCAGTAGTCGCGCCCCTCAAGTGTCAATAC

CGCAGGGCACTTATCCCCAGGCTTGTCCACATCATCTGTGGGAAACTCGCGTAAAATCAGGCGTTTTCGC

CGATTTGCGAGGCTGGCCAGCTCCACGTCGCCGGCCGAAATCGAGCCTGCCCCTCATCTGTCAACGCCGC

GCCGGGTGAGTCGGCCCCTCAAGTGTCAACGTCCGCCCCTCATCTGTCAGTGAGGGCCAAGTTTTCCGCG

AGGTATCCACAACGCCGGCGGCCGGCCGCGGTGTCTCGCACACGGCTTCGACGGCGTTTCTGGCGCGTTT

GCAGGGCCATAGACGGCCGCCAGCCCAGCGGCGAGGGCAACCAGCCCGGTGAGCGTCGGAAAGGGTCGAC

ATCTTGCTGCGTTCGGATATTTTCGTGGAGTTCCCGCCACAGACCCGGATTGAAGGCGAGATCCAGCAAC

TCGCGCCAGATCATCCTGTGACGGAACTTTGGCGCGTGATGACTGGCCAGGACGTCGGCCGAAAGAGCGA

CAAGCAGATCACGATTTTCGACAGCGTCGGATTTGCGATCGAGGATTTTTCGGCGCTGCGCTACGTCCGC

GACCGCGTTGAGGGATCAAGCCACAGCAGCCCACTCGACCTTCTAGCCGACCCAGACGAGCCAAGGGATC

TTTTTGGAATGCTGCTCCGTCGTCAGGCTTTCCGACGTTTGGGTGGTTGAACAGAAGTCATTATCGTACG

GAATGCCAGCACTCCCGAGGGGAACCCTGTGGTTGGCATGCACATACAAATGGACGAACGGATAAACCTT

TTCACGCCCTTTTAAATATCCGTTATTCTAATAAACGCTCTTTTCTCTTAGGTTTACCCGCCAATATATC

CTGTCAAACACTGATAGTTTAAACTGAAGGCGGGAAACGACAATCTGATCATGAGCGGAGAATTAAGGGA

GTCACGTTATGACCCCCGCCGATGACGCGGGACAAGCCGTTTTACGTTTGGAACTGACAGAACCGCAACG

ATTGAAGGAGCCACTCAGCCCCAATACGCAAACCGCCTCTCCCCGCGCGTTGGCCGATTCATTAATGCAG

CTGGCACGACAGGTTTCCCGACTGGAAAGCGGGCAGTGAGCGCAACGCAATTAATGTGAGTTAGCTCACT

CATTAGGCACCCCAGGCTTTACACTTTATGCTTCCGGCTCGTATGTTGTGTGGAATTGTGAGCGGATAAC

AATTTCACACAGGAAACAGCTATGACCATGATTACGCCAAGCTATTTAGGTGACACTATAGAATACTCAA

GCTATGCATCCAACGCGTTGGGAGCTCTCCCATATCGACCTGCAGGCGGCCGCTCGACGAATTAATTCCA

ATCCCACAAAAATCTGAGCTTAACAGCACAGTTGCTCCTCTCAGAGCAGAATCGGGTATTCAACACCCTC

ATATCAACTACTACGTTGTGTATAACGGTCCACATGCCGGTATATACGATGACTGGGGTTGTACAAAGGC

GGCAACAAACGGCGTTCCCGGAGTTGCACACAAGAAATTTGCCACTATTACAGAGGCAAGAGCAGCAGCT

GACGCGTACACAACAAGTCAGCAAACAGACAGGTTGAACTTCATCCCCAAAGGAGAAGCTCAACTCAAGC

CCAAGAGCTTTGCTAAGGCCCTAACAAGCCCACCAAAGCAAAAAGCCCACTGGCTCACGCTAGGAACCAA

AAGGCCCAGCAGTGATCCAGCCCCAAAAGAGATCTCCTTTGCCCCGGAGATTACAATGGACGATTTCCTC

TATCTTTACGATCTAGGAAGGAAGTTCGAAGGTGAAGGTGACGACACTATGTTCACCACTGATAATGAGA

AGGTTAGCCTCTTCAATTTCAGAAAGAATGCTGACCCACAGATGGTTAGAGAGGCCTACGCAGCAGGTCT

CATCAAGACGATCTACCCGAGTAACAATCTCCAGGAGATCAAATACCTTCCCAAGAAGGTTAAAGATGCA

GTCAAAAGATTCAGGACTAATTGCATCAAGAACACAGAGAAAGACATATTTCTCAAGATCAGAAGTACTA

TTCCAGTATGGACGATTCAAGGCTTGCTTCATAAACCAAGGCAAGTAATAGAGATTGGAGTCTCTAAAAA

GGTAGTTCCTACTGAATCTAAGGCCATGCATGGAGTCTAAGATTCAAATCGAGGATCTAACAGAACTCGC

CGTGAAGACTGGCGAACAGTTCATACAGAGTCTTTTACGACTCAATGACAAGAAGAAAATCTTCGTCAAC

ATGGTGGAGCACGACACTCTGGTCTACTCCAAAAATGTCAAAGATACAGTCTCAGAAGACCAAAGGGCTA

TTGAGACTTTTCAACAAAGGATAATTTCGGGAAACCTCCTCGGATTCCATTGCCCAGCTATCTGTCACTT

CATCGAAAGGACAGTAGAAAAGGAAGGTGGCTCCTACAAATGCCATCATTGCGATAAAGGAAAGGCTATC

ATTCAAGATCTCTCTGCCGACAGTGGTCCCAAAGATGGACCCCCACCCACGAGGAGCATCGTGGAAAAAG

AAGACGTTCCAACCACGTCTTCAAAGCAAGTGGATTGATGTGACATCTCCACTGACGTAAGGGATGACGC

ACAATCCCACTATCCTTCGCAAGACCCTTCCTCTATATAAGGAAGTTCATTTCATTTGGAGAGGACACGC

TCGACTATTTTTACAACAATTACC**ATGAAGACTAATCTTTTTCTCTTTCTCATCTTTTCACTTCTCCTAT**

**CATTATCCTCGGCCGAATTCCTCGAG**TCAC**CGCCGAAGGCTGAACTTCGACA***TATGTTAGCCATTAAATA*

*CCTA***TCTAGA**AGTAAAGGAGAAGAACTTTTCACTGGAGTTGTCCCAATTCTTGTTGAATTAGATGGTGAT

GTTAATGGGTACAAATTTTCTGTCAGTGGAGAGGGTGAAGGTGATGCAACATACGGAAAACTTACCCTTA

AATTTATTTGCACTACTGGAAAACTACCTGTTCCATGGCCAACACTTGTCACTACTTTCTCTTATGGTGT

TCAATGCTTTTCAAGATACCCAGATCATATGAAGCGGCACGACTTCTTCAAGAGCGCCATGCCTGAGGGA

TACGTGCAGGAGAGGACCATCTTCTTCAAGGACGACGGGAACTACAAGACACGTGCTGAAGTCAAGTTTG

AGGGAGACACCCTCGTCAACAGGATCGAGCTTAAGGGAATCGATTTCAAGGAGGACGGAAACATCCTCGG

CCACAAGTTGGAATACAACTACAACTCCCACAACGTATACATCATGGCCGACAAGCAAAAGAACGGCATC

AAAGCCAACTTCAAGACCCGCCACAACATCGAAGACGGCGGCGTGCAACTCGCTGATCATTATCAACAAA

ATACTCCAATTGGCGATGGCCCTGTCCTTTTACCAGACAACCATTACCTGTCCACACAATCTGCCCTTTC

GAAAGATCCCAACGAAAAGAGAGACCACATGGTCCTTCTTGAGTTTGTAACAGCTGCTGGGATTACACAT

GGCATGGATGAACTATACAAACATGATGAGCTTTGAACTAGAGTCCTGCTTTAATGAGATATGCGAGACG

CCTATGATCGCATGATATTTGCTTTCAATTCTGTTGTGCACGTTGTAAAAAACCTGAGCATGTGTAGCTC

AGATCCTTACCGCCGGTTTCGGTTCATTCTAATGAATATATCACCCGTTACTATCGTATTTTTATGAATA

ATATTCTCCGTTCAATTTACTGATTGTACCCTACTACTTATATGTACAATATTAAAATGAAAACAATATA

TTGTGCTGAATAGGTTTATAGCGACATCTATGATAGAGCGCCACAATAACAAACAATTGCGTTTTATTAT

TACAAATCCAATTTTAAAAAAAGCGGCAGAACCGGTCAAACCTAAAAGACTGATTACATAAATCTTATTC

AAATTTCAAAAGGCCCCAGGGGCTAGTATCTACGACACACCGAGCGGCGAACTAATAACGTTCACTGAAG

GGAACTCCGGTTCCCCGCCGGCGCGCATGGGTGAGATTCCTTGAAGTTGAGTATTGGCCGTCCGCTCTAC

CGAAAGTTACGGGCACCATTCAACCCGGTCCAGCACGGCGGCCGGGTAACCGACTTGCTGCCCCGAGAAT

TATGCAGCATTTTTTTGGTGTATGTGGGCCCCAAATGAAGTGCAGGTCAAACCTTGACAGTGACGACAAA

TCGTTGGGCGGGTCCAGGGCGAATTTTGCGACAACATGTCGAGGCTCAGCAGGACCTGCAGGCATGCAAG

CTAGCTTACTAGTGATGCATATTCTATAGTGTCACCTAAATCTGC

>pMS4-AMIRbC1 (**AK0068 (1bp - 14675bp, direct) 14675bp**)

GGCCGCACTAGTGATATCCCGCGGCCATGGCGGCCGGGAGCATGCGACGTCGGGCCCAATTCGCCCTATA

GTGAGTCGTATTACAATTCACTGGCCGTCGTTTTACAACGTCGTGACTGGGAAAACCCTGGCGTTACCCA

ACTTAATCGCCTTGCAGCACATCCCCCTTTCGCCAGCTGGCGTAATAGCGAAGAGGCCCGCACCGATCGC

CCTTCCCAACAGTTGCGCAGCCTGAATGGCGAATGGAAATTGTAAACGTTAATGGGTTTCTGGAGTTTAA

TGAGCTAAGCACATACGTCAGAAACCATTATTGCGCGTTCAAAAGTCGCCTAAGGTCACTATCAGCTAGC

AAATATTTCTTGTCAAAAATGCTCCACTGACGTTCCATAAATTCCCCTCGGTATCCAATTAGAGTCTCAT

ATTCACTCTCAATCCAAATAATCTGCAATGGCAATTACCTTATCCGCAACTTCTTTACCTATTTCCGCCC

GGATCCGGGCAGGTTCTCCGGCCGCTTGGGTGGAGAGGCTATTCGGCTATGACTGGGCACAACAGACAAT

CGGCTGCTCTGATGCCGCCGTGTTCCGGCTGTCAGCGCAGGGGCGCCCGGTTCTTTTTGTCAAGACCGAC

CTGTCCGGTGCCCTGAATGAACTGCAGGACGAGGCAGCGCGGCTATCGTGGCTGGCCACGACGGGCGTTC

CTTGCGCAGCTGTGCTCGACGTTGTCACTGAAGCGGGAAGGGACTGGCTGCTATTGGGCGAAGTGCCGGG

GCAGGATCTCCTGTCATCTCACCTTGCTCCTGCCGAGAAAGTATCCATCATGGCTGATGCAATGCGGCGG

CTGCATACGCTTGATCCGGCTACCTGCCCATTCGACCACCAAGCGAAACATCGCATCGAGCGAGCACGTA

CTCGGATGGAAGCCGGTCTTGTCGATCAGGATGATCTGGACGAAGAGCATCAGGGGCTCGCGCCAGCCGA

ACTGTTCGCCAGGCTCAAGGCGCGCATGCCCGACGGCGAGGATCTCGTCGTGACCCATGGCGATGCCTGC

TTGCCGAATATCATGGTGGAAAATGGCCGCTTTTCTGGATTCATCGACTGTGGCCGGCTGGGTGTGGCGG

ACCGCTATCAGGACATAGCGTTGGCTACCCGTGATATTGCTGAAGAGCTTGGCGGCGAATGGGCTGACCG

CTTCCTCGTGCTTTACGGTATCGCCGCTCCCGATTCGCAGCGCATCGCCTTCTATCGCCTTCTTGACGAG

TTCTTCTGAGCGGGACTCTGGGGTTCGAAATGACCGACCAAGCGACGCCCAACCTGCCATCACGAGATTT

CGATTCCACCGCCGCCTTCTATGAAAGGTTGGGCTTCGGAATCGTTTTCCGGGACGCCGGCTGGATGATC

CTCCAGCGCGGGGATCTCATGCTGGAGTTCTTCGCCCACCCCGATCCAACACTTACGTTTGCAACGTCCA

AGAGCAAATAGACCACGAACGCCGGAAGGTTGCCGCAGCGTGTGGATTGCGTCTCAATTCTCTCTTGCAG

GAATGCAATGATGAATATGATACTGACTATGAAACTTTGAGGGAATACTGCCTAGCACCGTCACCTCATA

ACGTGCATCATGCATGCCCTGACAACATGGAACATCGCTATTTTTCTGAAGAATTATGCTCGTTGGAGGA

TGTCGCGGCAATTGCAGCTATTGCCAACATCGAACTACCCCTCACGCATGCATTCATCAATATTATTCAT

GCGGGGAAAGGCAAGATTAATCCAACTGGCAAATCATCCAGCGTGATTGGTAACTTCAGTTCCAGCGACT

TGATTCGTTTTGGTGCTACCCACGTTTTCAATAAGGACGAGATGGTGGAGTAAAGAAGGAGTGCGTCGAA

GCAGATCGTTCAAACATTTGGCAATAAAGTTTCTTAAGATTGAATCCTGTTGCCGGTCTTGCGATGATTA

TCATATAATTTCTGTTGAATTACGTTAAGCATGTAATAATTAACATGTAATGCATGACGTTATTTATGAG

ATGGGTTTTTATGATTAGAGTCCCGCAATTATACATTTAATACGCGATAGAAAACAAAATATAGCGCGCA

AACTAGGATAAATTATCGCGCGCGGTGTCATCTATGTTACTAGATCGAATTAATTCCAGGCGGTGAAGGG

CAATCAGCTGTTGCCCGTCTCACTGGTGAAAAGAAAAACCACCCCAGTACATTAAAAACGTCCGCAATGT

GTTATTAAGTTGTCTAAGCGTCAATTTGTTTACACCACAATATATCCTGCCACCAGCCAGCCAACAGCTC

CCCGACCGGCAGCTCGGCACAAAATCACCACTCGATACAGGCAGCCCATCAGTCCGGGACGGCGTCAGCG

GGAGAGCCGTTGTAAGGCGGCAGACTTTGCTCATGTTACCGATGCTATTCGGAAGAACGGCAACTAAGCT

GCCGGGTTTGAAACACGGATGATCTCGCGGAGGGTAGCATGTTGATTGTAACGATGACAGAGCGTTGCTG

CCTGTGATCAAATATCATCTCCCTCGCAGAGATCCGAATTATCAGCCTTCTTATTCATTTCTCGCTTAAC

CGTGACAGGCTGTCGATCTTGAGAACTATGCCGACATAATAGGAAATCGCTGGATAAAGCCGCTGAGGAA

GCTGAGTGGCGCTATTTCTTTAGAAGTGAACGTTGACGATGTCGACGGATCTTTTCCGCTGCATAACCCT

GCTTCGGGGTCATTATAGCGATTTTTTCGGTATATCCATCCTTTTTCGCACGATATACAGGATTTTGCCA

AAGGGTTCGTGTAGACTTTCCTTGGTGTATCCAACGGCGTCAGCCGGGCAGGATAGGTGAAGTAGGCCCA

CCCGCGAGCGGGTGTTCCTTCTTCACTGTCCCTTATTCGCACCTGGCGGTGCTCAACGGGAATCCTGCTC

TGCGAGGCTGGCCGGCTACCGCCGGCGTAACAGATGAGGGCAAGCGGATGGCTGATGAAACCAAGCCAAC

CAGGGGTGATGCTGCCAACTTACTGATTTAGTGTATGATGGTGTTTTTGAGGTGCTCCAGTGGCTTCTGT

TTCTATCAGCTGTCCCTCCTGTTCAGCTACTGACGGGGTGGTGCGTAACGGCAAAAGCACCGCCGGACAT

CAGCGCTATCTCTGCTCTCACTGCCGTAAAACATGGCAACTGCAGTTCACTTACACCGCTTCTCAACCCG

GTACGCACCAGAAAATCATTGATATGGCCATGAATGGCGTTGGATGCCGGGCAACAGCCCGCATTATGGG

CGTTGGCCTCAACACGATTTTACGTCACTTAAAAAACTCAGGCCGCAGTCGGTAACCTCGCGCATACAGC

CGGGCAGTGACGTCATCGTCTGCGCGGAAATGGACGAACAGTGGGGCTATGTCGGGGCTAAATCGCGCCA

GCGCTGGCTGTTTTACGCGTATGACAGTCTCCGGAAGACGGTTGTTGCGCACGTATTCGGTGAACGCACT

ATGGCGACGCTGGGGCGTCTTATGAGCCTGCTGTCACCCTTTGACGTGGTGATATGGATGACGGATGGCT

GGCCGCTGTATGAATCCCGCCTGAAGGGAAAGCTGCACGTAATCAGCAAGCGATATACGCAGCGAATTGA

GCGGCATAACCTGAATCTGAGGCAGCACCTGGCACGGCTGGGACGGAAGTCGCTGTCGTTCTCAAAATCG

GTGGAGCTGCATGACAAAGTCATCGGGCATTATCTGAACATAAAACACTATCAATAAGTTGGAGTCATTA

CCCAACCAGGAAGGGCAGCCCACCTATCAAGGTGTACTGCCTTCCAGACGAACGAAGAGCGATTGAGGAA

AAGGCGGCGGCGGCCGGCATGAGCCTGTCGGCCTACCTGCTGGCCGTCGGCCAGGGCTACAAAATCACGG

GCGTCGTGGACTATGAGCACGTCCGCGAGCTGGCCCGCATCAATGGCGACCTGGGCCGCCTGGGCGGCCT

GCTGAAACTCTGGCTCACCGACGACCCGCGCACGGCGCGGTTCGGTGATGCCACGATCCTCGCCCTGCTG

GCGAAGATCGAAGAGAAGCAGGACGAGCTTGGCAAGGTCATGATGGGCGTGGTCCGCCCGAGGGCAGAGC

CATGACTTTTTTAGCCGCTAAAACGGCCGGGGGGTGCGCGTGATTGCCAAGCACGTCCCCATGCGCTCCA

TCAAGAAGAGCGACTTCGCGGAGCTGGTATTCGTGCAGGGCAAGATTCGGAATACCAAGTACGAGAAGGA

CGGCCAGACGGTCTACGGGACCGACTTCATTGCCGATAAGGTGGATTATCTGGACACCAAGGCACCAGGC

GGGTCAAATCAGGAATAAGGGCACATTGCCCCGGCGTGAGTCGGGGCAATCCCGCAAGGAGGGTGAATGA

ATCGGACGTTTGACCGGAAGGCATACAGGCAAGAACTGATCGACGCGGGGTTTTCCGCCGAGGATGCCGA

AACCATCGCAAGCCGCACCGTCATGCGTGCGCCCCGCGAAACCTTCCAGTCCGTCGGCTCGATGGTCCAG

CAAGCTACGGCCAAGATCGAGCGCGACAGCGTGCAACTGGCTCCCCCTGCCCTGCCCGCGCCATCGGCCG

CCGTGGAGCGTTCGCGTCGTCTCGAACAGGAGGCGGCAGGTTTGGCGAAGTCGATGACCATCGACACGCG

AGGAACTATGACGACCAAGAAGCGAAAAACCGCCGGCGAGGACCTGGCAAAACAGGTCAGCGAGGCCAAG

CAGGCCGCGTTGCTGAAACACACGAAGCAGCAGATCAAGGAAATGCAGCTTTCCTTGTTCGATATTGCGC

CGTGGCCGGACACGATGCGAGCGATGCCAAACGACACGGCCCGCTCTGCCCTGTTCACCACGCGCAACAA

GAAAATCCCGCGCGAGGCGCTGCAAAACAAGGTCATTTTCCACGTCAACAAGGACGTGAAGATCACCTAC

ACCGGCGTCGAGCTGCGGGCCGACGATGACGAACTGGTGTGGCAGCAGGTGTTGGAGTACGCGAAGCGCA

CCCCTATCGGCGAGCCGATCACCTTCACGTTCTACGAGCTTTGCCAGGACCTGGGCTGGTCGATCAATGG

CCGGTATTACACGAAGGCCGAGGAATGCCTGTCGCGCCTACAGGCGACGGCGATGGGCTTCACGTCCGAC

CGCGTTGGGCACCTGGAATCGGTGTCGCTGCTGCACCGCTTCCGCGTCCTGGACCGTGGCAAGAAAACGT

CCCGTTGCCAGGTCCTGATCGACGAGGAAATCGTCGTGCTGTTTGCTGGCGACCACTACACGAAATTCAT

ATGGGAGAAGTACCGCAAGCTGTCGCCGACGGCCCGACGGATGTTCGACTATTTCAGCTCGCACCGGGAG

CCGTACCCGCTCAAGCTGGAAACCTTCCGCCTCATGTGCGGATCGGATTCCACCCGCGTGAAGAAGTGGC

GCGAGCAGGTCGGCGAAGCCTGCGAAGAGTTGCGAGGCAGCGGCCTGGTGGAACACGCCTGGGTCAATGA

TGACCTGGTGCATTGCAAACGCTAGGGCCTTGTGGGGTCAGTTCCGGCTGGGGGTTCAGCAGCCAGCGCT

TTACTGGCATTTCAGGAACAAGCGGGCACTGCTCGACGCACTTGCTTCGCTCAGTATCGCTCGGGACGCA

CGGCGCGCTCTACGAACTGCCGATAAACAGAGGATTAAAATTGACAATTGTGATTAAGGCTCAGATTCGA

CGGCTTGGAGCGGCCGACGTGCAGGATTTCCGCGAGATCCGATTGTCGGCCCTGAAGAAAGCTCCAGAGA

TGTTCGGGTCCGTTTACGAGCACGAGGAGAAAAAGCCCATGGAGGCGTTCGCTGAACGGTTGCGAGATGC

CGTGGCATTCGGCGCCTACATCGACGGCGAGATCATTGGGCTGTCGGTCTTCAAACAGGAGGACGGCCCC

AAGGACGCTCACAAGGCGCATCTGTCCGGCGTTTTCGTGGAGCCCGAACAGCGAGGCCGAGGGGTCGCCG

GTATGCTGCTGCGGGCGTTGCCGGCGGGTTTATTGCTCGTGATGATCGTCCGACAGATTCCAACGGGAAT

CTGGTGGATGCGCATCTTCATCCTCGGCGCACTTAATATTTCGCTATTCTGGAGCTTGTTGTTTATTTCG

GTCTACCGCCTGCCGGGCGGGGTCGCGGCGACGGTAGGCGCTGTGCAGCCGCTGATGGTCGTGTTCATCT

CTGCCGCTCTGCTAGGTAGCCCGATACGATTGATGGCGGTCCTGGGGGCTATTTGCGGAACTGCGGGCGT

GGCGCTGTTGGTGTTGACACCAAACGCAGCGCTAGATCCTGTCGGCGTCGCAGCGGGCCTGGCGGGGGCG

GTTTCCATGGCGTTCGGAACCGTGCTGACCCGCAAGTGGCAACCTCCCGTGCCTCTGCTCACCTTTACCG

CCTGGCAACTGGCGGCCGGAGGACTTCTGCTCGTTCCAGTAGCTTTAGTGTTTGATCCGCCAATCCCGAT

GCCTACAGGAACCAATGTTCTCGGCCTGGCGTGGCTCGGCCTGATCGGAGCGGGTTTAACCTACTTCCTT

TGGTTCCGGGGGATCTCGCGACTCGAACCTACAGTTGTTTCCTTACTGGGCTTTCTCAGCCGGGATGGCG

CTAAGAAGCTATTGCCGCCGATCTTCATATGCGGTGTGAAATACCGCACAGATGCGTAAGGAGAAAATAC

CGCATCAGGCGCTCTTCCGCTTCCTCGCTCACTGACTCGCTGCGCTCGGTCGTTCGGCTGCGGCGAGCGG

TATCAGCTCACTCAAAGGCGGTAATACGGTTATCCACAGAATCAGGGGATAACGCAGGAAAGAACATGTG

AGCAAAAGGCCAGCAAAAGGCCAGGAACCGTAAAAAGGCCGCGTTGCTGGCGTTTTTCCATAGGCTCCGC

CCCCCTGACGAGCATCACAAAAATCGACGCTCAAGTCAGAGGTGGCGAAACCCGACAGGACTATAAAGAT

ACCAGGCGTTTCCCCCTGGAAGCTCCCTCGTGCGCTCTCCTGTTCCGACCCTGCCGCTTACCGGATACCT

GTCCGCCTTTCTCCCTTCGGGAAGCGTGGCGCTTTCTCAATGCTCACGCTGTAGGTATCTCAGTTCGGTG

TAGGTCGTTCGCTCCAAGCTGGGCTGTGTGCACGAACCCCCCGTTCAGCCCGACCGCTGCGCCTTATCCG

GTAACTATCGTCTTGAGTCCAACCCGGTAAGACACGACTTATCGCCACTGGCAGCAGCCACTGGTAACAG

GATTAGCAGAGCGAGGTATGTAGGCGGTGCTACAGAGTTCTTGAAGTGGTGGCCTAACTACGGCTACACT

AGAAGGACAGTATTTGGTATCTGCGCTCTGCTGAAGCCAGTTACCTTCGGAAAAAGAGTTGGTAGCTCTT

GATCCGGCAAACAAACCACCGCTGGTAGCGGTGGTTTTTTTGTTTGCAAGCAGCAGATTACGCGCAGAAA

AAAAGGATATCAAGAAGATCCTTTGATCTTTTCTACGGGGTCTGACGCTCAGTGGAACGAAAACTCACGT

TAAGGGATTTTGGTCATGAGATTATCAAAAAGGATCTTCACCTAGATCCTTTTAAATTAAAAATGAAGTT

TTAAATCAATCTAAAGTATATATGAGTAAACTTGGTCTGACAGTTACCAATGCTTAATCAGTGAGGCACC

TATCTCAGCGATCTGTCTATTTCGTTCATCCATAGTTGCCTGACTCCCCGTCGTGTAGATAACTACGATA

CGGGAGGGCTTACCATCTGGCCCCAGTGCTGCAATGATACCGCGAGACCCACGCTCACCGGCTCCAGATT

TATCAGCAATAAACCAGCCAGCCGGAAGGGCCGAGCGCAGAAGTGGTCCTGCAACTTTATCCGCCTCCAT

CCAGTCTATTAAACAAGTGGCAGCAACGGATTCGCAAACCTGTCACGCCTTTTGTGCCAAAAGCCGCGCC

AGGTTTGCGATCCGCTGTGCCAGGCGTTAGGCGTCATATGAAGATTTCGGTGATCCCTGAGCAGGTGGCG

GAAACATTGGATGCTGAGAACCATTTCATTGTTCGTGAAGTGTTCGATGTGCACCTATCCGACCAAGGCT

TTGAACTATCTACCAGAAGTGTGAGCCCCTACCGGAAGGATTACATCTCGGATGATGACTCTGATGAAGA

CTCTGCTTGCTATGGCGCATTCATCGACCAAGAGCTTGTCGGGAAGATTGAACTCAACTCAACATGGAAC

GATCTAGCCTCTATCGAACACATTGTTGTGTCGCACACGCACCGAGGCAAAGGAGTCGCGCACAGTCTCA

TCGAATTTGCGAAAAAGTGGGCACTAAGCAGACAGCTCCTTGGCATACGATTAGAGACACAAACGAACAA

TGTACCTGCCTGCAATTTGTACGCAAAATGTGGCTTTACTCTCGGCGGCATTGACCTGTTCACGTATAAA

ACTAGACCTCAAGTCTCGAACGAAACAGCGATGTACTGGTACTGGTTCTCGGGAGCACAGGATGACGCCT

AACAATTCATTCAAGCCGACACCGCTTCGCGGCGCGGCTTAATTCAGGAGTTAAACATCATGAGGGAAGC

GGTGATCGCCGAAGTATCGACTCAACTATCAGAGGTAGTTGGCGTCATCGAGCGCCATCTCGAACCGACG

TTGCTGGCCGTACATTTGTACGGCTCCGCAGTGGATGGCGGCCTGAAGCCACACAGTGATATTGATTTGC

TGGTTACGGTGACCGTAAGGCTTGATGAAACAACGCGGCGAGCTTTGATCAACGACCTTTTGGAAACTTC

GGCTTCCCCTGGAGAGAGCGAGATTCTCCGCGCTGTAGAAGTCACCATTGTTGTGCACGACGACATCATT

CCGTGGCGTTATCCAGCTAAGCGCGAACTGCAATTTGGAGAATGGCAGCGCAATGACATTCTTGCAGGTA

TCTTCGAGCCAGCCACGATCGACATTGATCTGGCTATCTTGCTGACAAAAGCAAGAGAACATAGCGTTGC

CTTGGTAGGTCCAGCGGCGGAGGAACTCTTTGATCCGGTTCCTGAACAGGATCTATTTGAGGCGCTAAAT

GAAACCTTAACGCTATGGAACTCGCCGCCCGACTGGGCTGGCGATGAGCGAAATGTAGTGCTTACGTTGT

CCCGCATTTGGTACAGCGCAGTAACCGGCAAAATCGCGCCGAAGGATGTCGCTGCCGACTGGGCAATGGA

GCGCCTGCCGGCCCAGTATCAGCCCGTCATACTTGAAGCTAGGCAGGCTTATCTTGGACAAGAAGATCGC

TTGGCCTCGCGCGCAGATCAGTTGGAAGAATTTGTTCACTACGTGAAAGGCGAGATCACCAAGGTAGTCG

GCAAATAATGTCTAACAATTCGTTCAAGCCGACGCCGCTTCGCGGCGCGGCTTAACTCAAGCGTTAGAGA

GCTGGGGAAGACTATGCGCGATCTGTTGAAGGTGGTTCTAAGCCTCGTACTTGCGATGGCATCGGGGCAG

GCACTTGCTGACCTGCCAATTGTTTTAGTGGATGAAGCTCGTCTTCCCTATGACTACTCCCCATCCAACT

ACGACATTTCTCCAAGCAACTACGACAACTCCATAAGCAATTACGACAATAGTCCATCAAATTACGACAA

CTCTGAGAGCAACTACGATAATAGTTCATCCAATTACGACAATAGTCGCAACGGAAATCGTAGGCTTATA

TATAGCGCAAATGGGTCTCGCACTTTCGCCGGCTACTACGTCATTGCCAACAATGGGACAACGAACTTCT

TTTCCACATCTGGCAAAAGGATGTTCTACACCCCAAAAGGGGGGCGCGGCGTCTATGGCGGCAAAGATGG

GAGCTTCTGCGGGGCATTGGTCGTCATAAATGGCCAATTTTCGCTTGCCCTGACAGATAACGGCCTGAAG

ATCATGTATCTAAGCAACTAGCCTGCTCTCTAATAAAATGTTAGGAGCTTGGCTGCCATTTTTGGGGTGA

GGCCGTTCGCGGCCGAGGGGCGCAGCCCCTGGGGGGATGGGAGGCCCGCGTTAGCGGGCCGGGAGGGTTC

GAGAAGGGGGGGCACCCCCCTTCGGCGTGCGCGGTCACGCGCCAGGGCGCAGCCCTGGTTAAAAACAAGG

TTTATAAATATTGGTTTAAAAGCAGGTTAAAAGACAGGTTAGCGGTGGCCGAAAAACGGGCGGAAACCCT

TGCAAATGCTGGATTTTCTGCCTGTGGACAGCCCCTCAAATGTCAATAGGTGCGCCCCTCATCTGTCAGC

ACTCTGCCCCTCAAGTGTCAAGGATCGCGCCCCTCATCTGTCAGTAGTCGCGCCCCTCAAGTGTCAATAC

CGCAGGGCACTTATCCCCAGGCTTGTCCACATCATCTGTGGGAAACTCGCGTAAAATCAGGCGTTTTCGC

CGATTTGCGAGGCTGGCCAGCTCCACGTCGCCGGCCGAAATCGAGCCTGCCCCTCATCTGTCAACGCCGC

GCCGGGTGAGTCGGCCCCTCAAGTGTCAACGTCCGCCCCTCATCTGTCAGTGAGGGCCAAGTTTTCCGCG

AGGTATCCACAACGCCGGCGGCCGGCCGCGGTGTCTCGCACACGGCTTCGACGGCGTTTCTGGCGCGTTT

GCAGGGCCATAGACGGCCGCCAGCCCAGCGGCGAGGGCAACCAGCCCGGTGAGCGTCGGAAAGGGTCGAC

ATCTTGCTGCGTTCGGATATTTTCGTGGAGTTCCCGCCACAGACCCGGATTGAAGGCGAGATCCAGCAAC

TCGCGCCAGATCATCCTGTGACGGAACTTTGGCGCGTGATGACTGGCCAGGACGTCGGCCGAAAGAGCGA

CAAGCAGATCACGATTTTCGACAGCGTCGGATTTGCGATCGAGGATTTTTCGGCGCTGCGCTACGTCCGC

GACCGCGTTGAGGGATCAAGCCACAGCAGCCCACTCGACCTTCTAGCCGACCCAGACGAGCCAAGGGATC

TTTTTGGAATGCTGCTCCGTCGTCAGGCTTTCCGACGTTTGGGTGGTTGAACAGAAGTCATTATCGTACG

GAATGCCAGCACTCCCGAGGGGAACCCTGTGGTTGGCATGCACATACAAATGGACGAACGGATAAACCTT

TTCACGCCCTTTTAAATATCCGTTATTCTAATAAACGCTCTTTTCTCTTAGGTTTACCCGCCAATATATC

CTGTCAAACACTGATAGTTTAAACTGAAGGCGGGAAACGACAATCTGATCATGAGCGGAGAATTAAGGGA

GTCACGTTATGACCCCCGCCGATGACGCGGGACAAGCCGTTTTACGTTTGGAACTGACAGAACCGCAACG

ATTGAAGGAGCCACTCAGCCCCAATACGCAAACCGCCTCTCCCCGCGCGTTGGCCGATTCATTAATGCAG

CTGGCACGACAGGTTTCCCGACTGGAAAGCGGGCAGTGAGCGCAACGCAATTAATGTGAGTTAGCTCACT

CATTAGGCACCCCAGGCTTTACACTTTATGCTTCCGGCTCGTATGTTGTGTGGAATTGTGAGCGGATAAC

AATTTCACACAGGAAACAGCTATGACCATGATTACGCCAAGCTATTTAGGTGACACTATAGAATACTCAA

GCTATGCATCCAACGCGTTGGGAGCTCTCCCATATCGACCTGCAGGCGGCCGCTCGACGAATTAATTCCA

ATCCCACAAAAATCTGAGCTTAACAGCACAGTTGCTCCTCTCAGAGCAGAATCGGGTATTCAACACCCTC

ATATCAACTACTACGTTGTGTATAACGGTCCACATGCCGGTATATACGATGACTGGGGTTGTACAAAGGC

GGCAACAAACGGCGTTCCCGGAGTTGCACACAAGAAATTTGCCACTATTACAGAGGCAAGAGCAGCAGCT

GACGCGTACACAACAAGTCAGCAAACAGACAGGTTGAACTTCATCCCCAAAGGAGAAGCTCAACTCAAGC

CCAAGAGCTTTGCTAAGGCCCTAACAAGCCCACCAAAGCAAAAAGCCCACTGGCTCACGCTAGGAACCAA

AAGGCCCAGCAGTGATCCAGCCCCAAAAGAGATCTCCTTTGCCCCGGAGATTACAATGGACGATTTCCTC

TATCTTTACGATCTAGGAAGGAAGTTCGAAGGTGAAGGTGACGACACTATGTTCACCACTGATAATGAGA

AGGTTAGCCTCTTCAATTTCAGAAAGAATGCTGACCCACAGATGGTTAGAGAGGCCTACGCAGCAGGTCT

CATCAAGACGATCTACCCGAGTAACAATCTCCAGGAGATCAAATACCTTCCCAAGAAGGTTAAAGATGCA

GTCAAAAGATTCAGGACTAATTGCATCAAGAACACAGAGAAAGACATATTTCTCAAGATCAGAAGTACTA

TTCCAGTATGGACGATTCAAGGCTTGCTTCATAAACCAAGGCAAGTAATAGAGATTGGAGTCTCTAAAAA

GGTAGTTCCTACTGAATCTAAGGCCATGCATGGAGTCTAAGATTCAAATCGAGGATCTAACAGAACTCGC

CGTGAAGACTGGCGAACAGTTCATACAGAGTCTTTTACGACTCAATGACAAGAAGAAAATCTTCGTCAAC

ATGGTGGAGCACGACACTCTGGTCTACTCCAAAAATGTCAAAGATACAGTCTCAGAAGACCAAAGGGCTA

TTGAGACTTTTCAACAAAGGATAATTTCGGGAAACCTCCTCGGATTCCATTGCCCAGCTATCTGTCACTT

CATCGAAAGGACAGTAGAAAAGGAAGGTGGCTCCTACAAATGCCATCATTGCGATAAAGGAAAGGCTATC

ATTCAAGATCTCTCTGCCGACAGTGGTCCCAAAGATGGACCCCCACCCACGAGGAGCATCGTGGAAAAAG

AAGACGTTCCAACCACGTCTTCAAAGCAAGTGGATTGATGTGACATCTCCACTGACGTAAGGGATGACGC

ACAATCCCACTATCCTTCGCAAGACCCTTCCTCTATATAAGGAAGTTCATTTCATTTGGAGAGGACACGC

TCGACTATTTTTACAACAATTACC**ATGAAGACTAATCTTTTTCTCTTTCTCATCTTTTCACTTCTCCTAT**

**CATTATCCTCGGCCGAATTCCTCGAG**TCAC**TTAGCCAAGAAGAAATTCATGA***CAGGCAAGAAGATATGGTACAA***TCTAGA**AGTAAAGGAGAAGAACTTTTCACTGGAGTTGTCCCAATTCTTGTTGAATTAGATGGTGAT

GTTAATGGGTACAAATTTTCTGTCAGTGGAGAGGGTGAAGGTGATGCAACATACGGAAAACTTACCCTTA

AATTTATTTGCACTACTGGAAAACTACCTGTTCCATGGCCAACACTTGTCACTACTTTCTCTTATGGTGT

TCAATGCTTTTCAAGATACCCAGATCATATGAAGCGGCACGACTTCTTCAAGAGCGCCATGCCTGAGGGA

TACGTGCAGGAGAGGACCATCTTCTTCAAGGACGACGGGAACTACAAGACACGTGCTGAAGTCAAGTTTG

AGGGAGACACCCTCGTCAACAGGATCGAGCTTAAGGGAATCGATTTCAAGGAGGACGGAAACATCCTCGG

CCACAAGTTGGAATACAACTACAACTCCCACAACGTATACATCATGGCCGACAAGCAAAAGAACGGCATC

AAAGCCAACTTCAAGACCCGCCACAACATCGAAGACGGCGGCGTGCAACTCGCTGATCATTATCAACAAA

ATACTCCAATTGGCGATGGCCCTGTCCTTTTACCAGACAACCATTACCTGTCCACACAATCTGCCCTTTC

GAAAGATCCCAACGAAAAGAGAGACCACATGGTCCTTCTTGAGTTTGTAACAGCTGCTGGGATTACACAT

GGCATGGATGAACTATACAAACATGATGAGCTTTGAACTAGAGTCCTGCTTTAATGAGATATGCGAGACG

CCTATGATCGCATGATATTTGCTTTCAATTCTGTTGTGCACGTTGTAAAAAACCTGAGCATGTGTAGCTC

AGATCCTTACCGCCGGTTTCGGTTCATTCTAATGAATATATCACCCGTTACTATCGTATTTTTATGAATA

ATATTCTCCGTTCAATTTACTGATTGTACCCTACTACTTATATGTACAATATTAAAATGAAAACAATATA

TTGTGCTGAATAGGTTTATAGCGACATCTATGATAGAGCGCCACAATAACAAACAATTGCGTTTTATTAT

TACAAATCCAATTTTAAAAAAAGCGGCAGAACCGGTCAAACCTAAAAGACTGATTACATAAATCTTATTC

AAATTTCAAAAGGCCCCAGGGGCTAGTATCTACGACACACCGAGCGGCGAACTAATAACGTTCACTGAAG

GGAACTCCGGTTCCCCGCCGGCGCGCATGGGTGAGATTCCTTGAAGTTGAGTATTGGCCGTCCGCTCTAC

CGAAAGTTACGGGCACCATTCAACCCGGTCCAGCACGGCGGCCGGGTAACCGACTTGCTGCCCCGAGAAT

TATGCAGCATTTTTTTGGTGTATGTGGGCCCCAAATGAAGTGCAGGTCAAACCTTGACAGTGACGACAAA

TCGTTGGGCGGGTCCAGGGCGAATTTTGCGACAACATGTCGAGGCTCAGCAGGACCTGCAGGCATGCAAG

CTAGCTTACTAGTGATGCATATTCTATAGTGTCACCTAAATCTGC
