# Supplementary material for: Large Artificial microRNA Cluster Genes Confer Effective Resistance against Multiple Tomato Yellow Leaf Curl Viruses in Transgenic Tomato
Source: Plants (Basel). 2023 May 31;12(11):2179. doi: 10.3390/plants12112179 (PMC10255879; doi:10.3390/plants12112179)
Supplement: Supplementary file 1 [file plants-12-02179-s001.zip › plants-2357753-supplementary/plants-2357753-SM/Supplemental Figures-AK2023-F.pptx]

## Slide 1
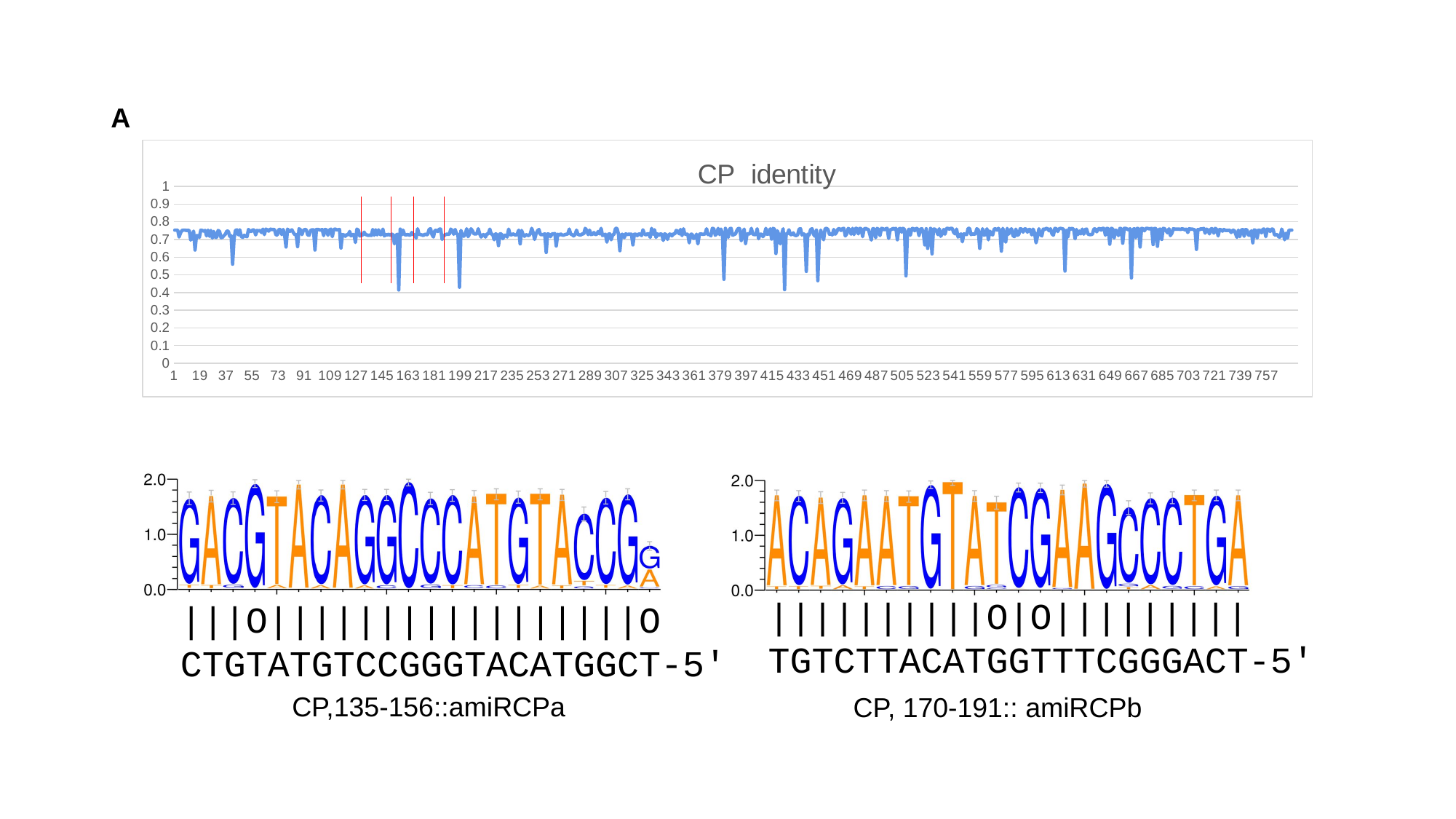

A
### Chart: CP identity
| Category | identity |
|---|---|
|||O|||||||||||||||||O
CTGTATGTCCGGGTACATGGCT-5'
CP,135-156::amiRCPa
||||||||||O|O|||||||||
TGTCTTACATGGTTTCGGGACT-5'
CP, 170-191:: amiRCPb

## Slide 2
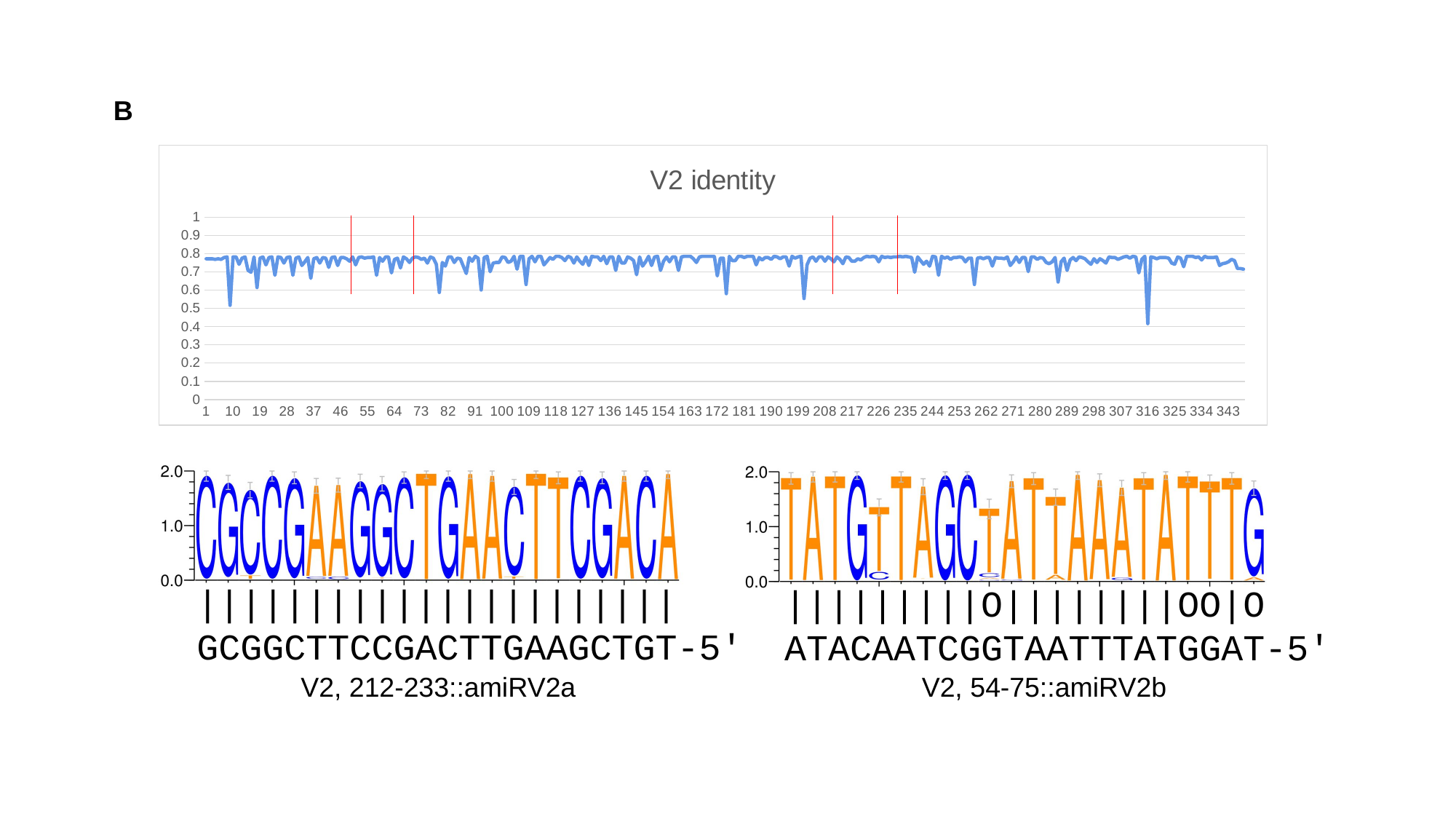

B
### Chart: V2 identity
| Category | identity |
|---|---|
||||||||||||||||||||||
GCGGCTTCCGACTTGAAGCTGT-5'
V2, 212-233::amiRV2a
|||||||||O||||||||OO|O
ATACAATCGGTAATTTATGGAT-5'
V2, 54-75::amiRV2b

## Slide 3
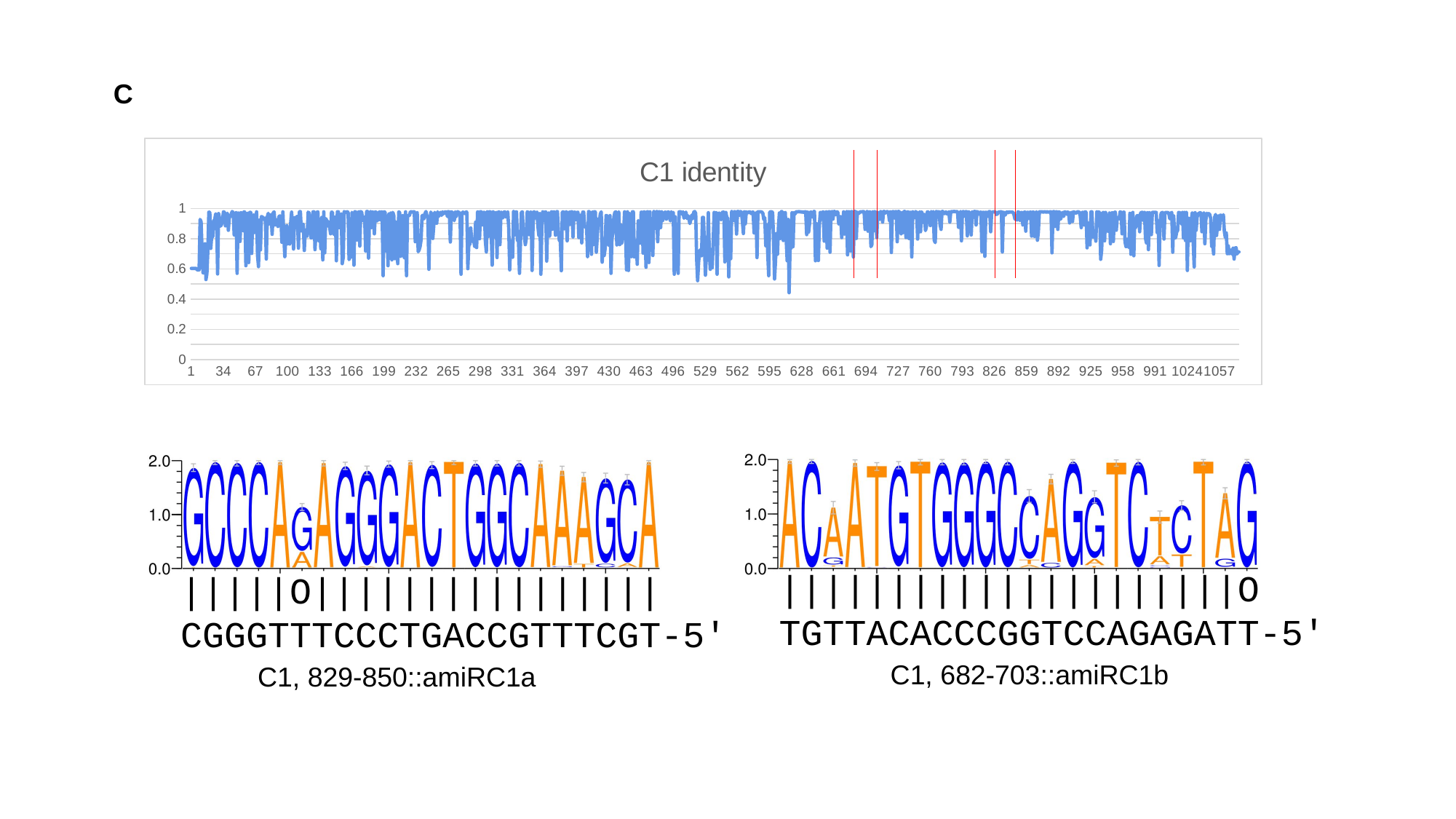

C
### Chart: C1 identity
| Category | identity |
|---|---|
|||||O||||||||||||||||
CGGGTTTCCCTGACCGTTTCGT-5'
C1, 829-850::amiRC1a
|||||||||||||||||||||O
TGTTACACCCGGTCCAGAGATT-5'
C1, 682-703::amiRC1b

## Slide 4
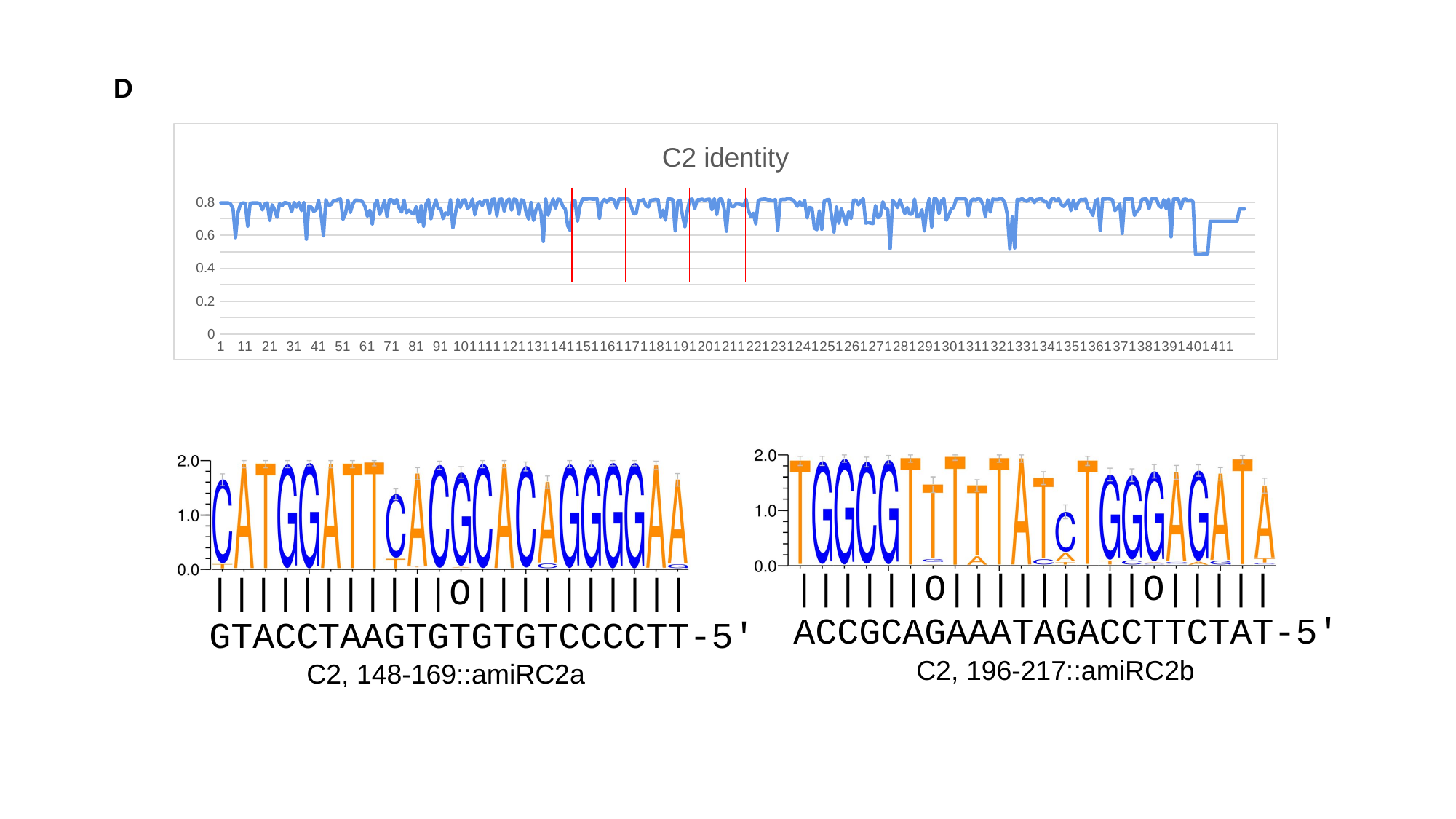

D
### Chart: C2 identity
| Category | identity |
|---|---|
||||||O|||||||||O|||||
ACCGCAGAAATAGACCTTCTAT-5'
C2, 196-217::amiRC2b
|||||||||||O||||||||||
GTACCTAAGTGTGTGTCCCCTT-5'
C2, 148-169::amiRC2a

## Slide 5
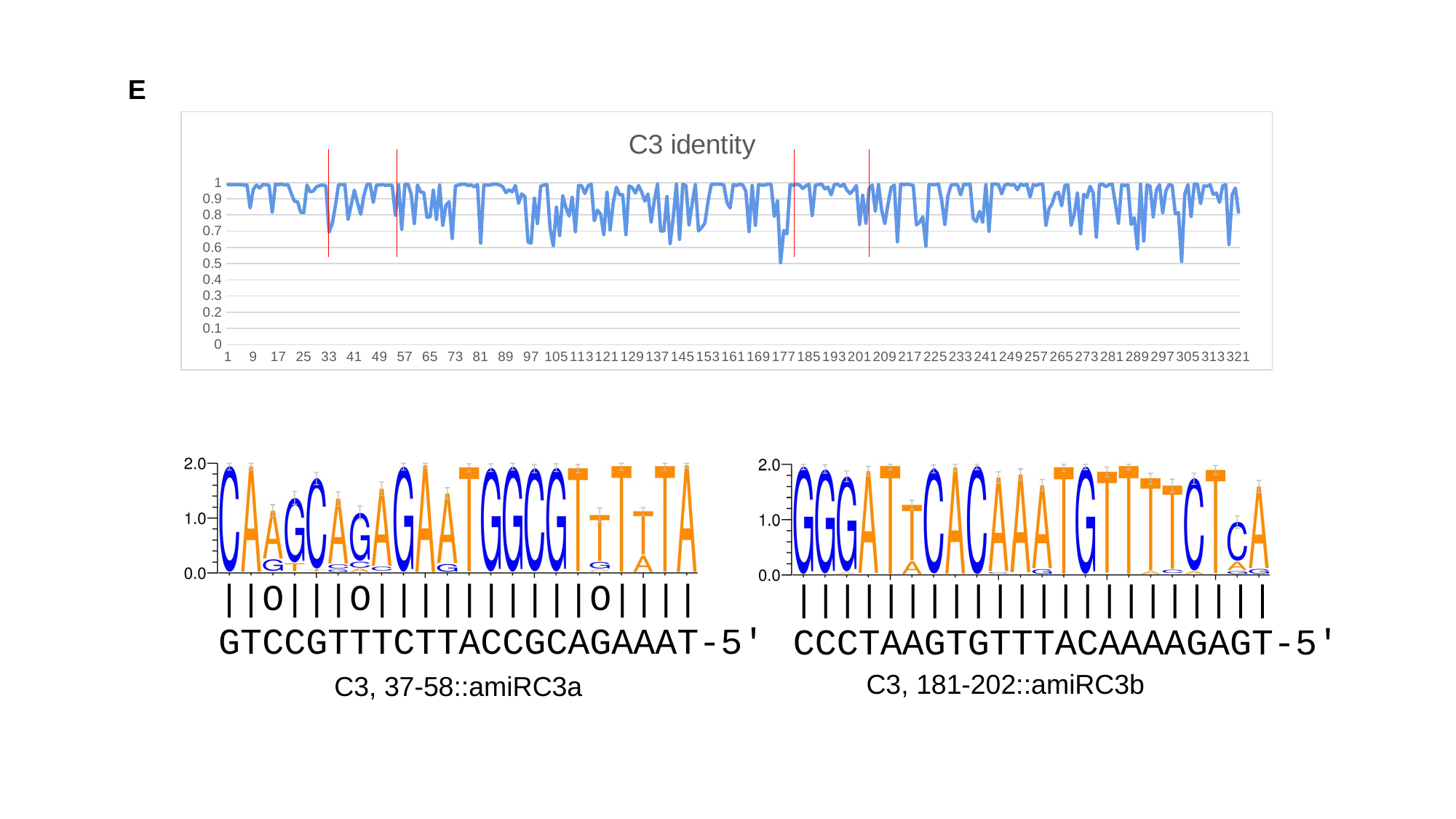

E
### Chart: C3 identity
| Category | identity |
|---|---|
||O|||O||||||||||O||||
GTCCGTTTCTTACCGCAGAAAT-5'
C3, 37-58::amiRC3a
||||||||||||||||||||||
CCCTAAGTGTTTACAAAAGAGT-5'
C3, 181-202::amiRC3b

## Slide 6
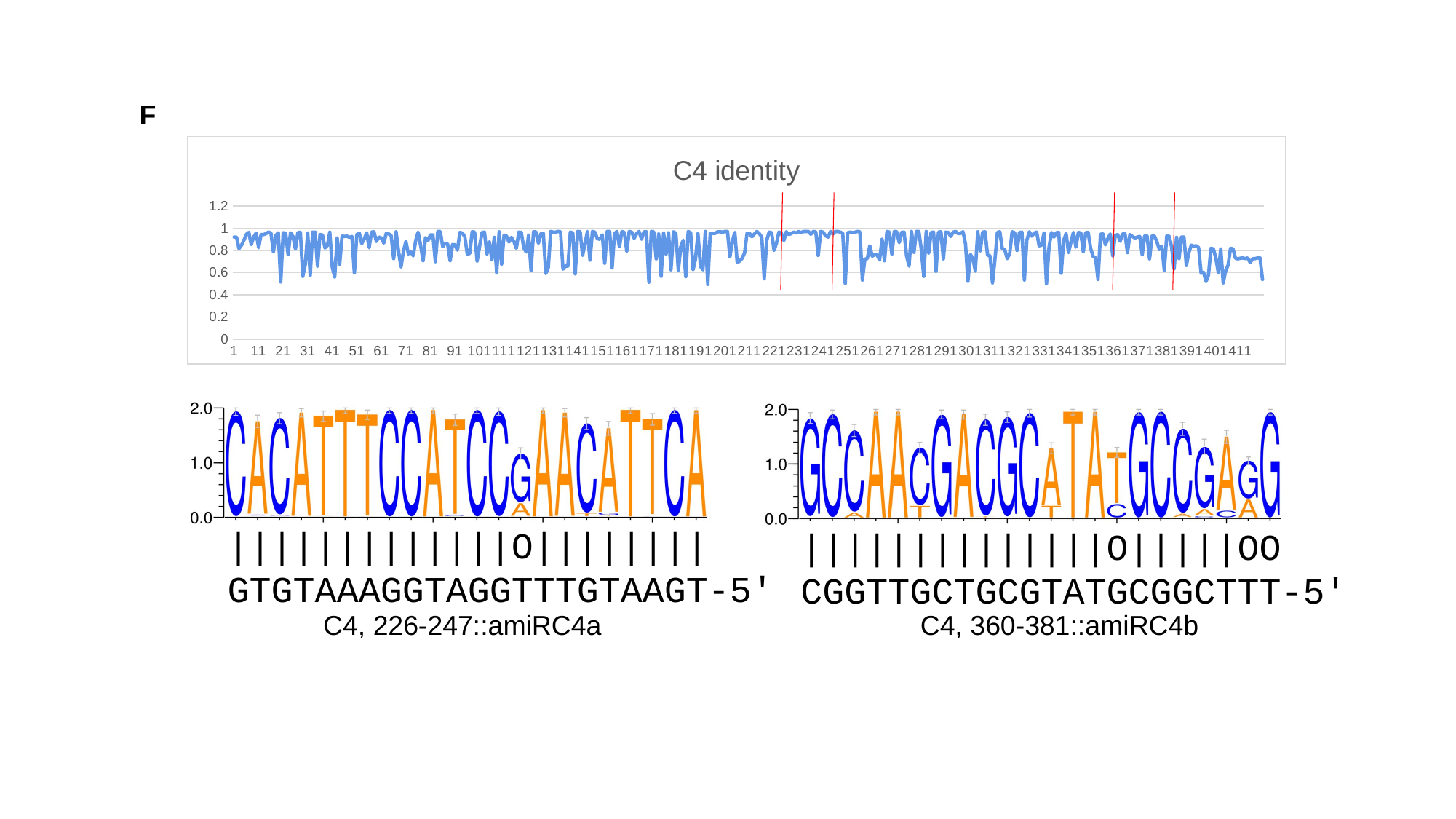

F
### Chart: C4 identity
| Category | identity |
|---|---|
|||||||||||||O||||||||
GTGTAAAGGTAGGTTTGTAAGT-5'
C4, 226-247::amiRC4a
||||||||||||||O|||||OO
CGGTTGCTGCGTATGCGGCTTT-5'
C4, 360-381::amiRC4b

## Slide 7
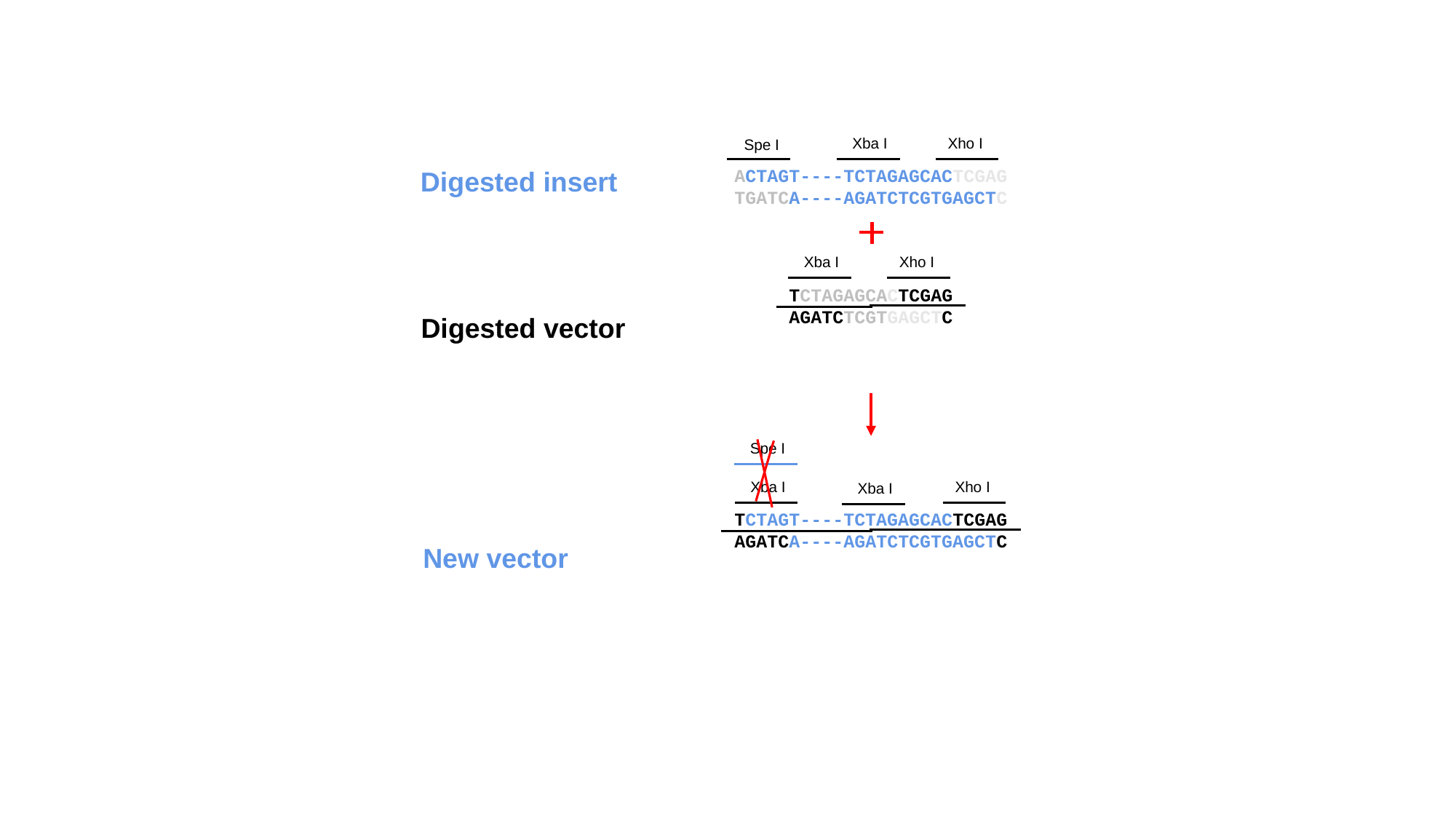

Xho I
Xba I
Spe I
ACTAGT----TCTAGAGCACTCGAG
TGATCA----AGATCTCGTGAGCTC
Digested insert
Xho I
Xba I
TCTAGAGCACTCGAG
AGATCTCGTGAGCTC
Digested vector
Spe I
Xho I
Xba I
Xba I
TCTAGT----TCTAGAGCACTCGAG
AGATCA----AGATCTCGTGAGCTC
New vector

## Slide 8
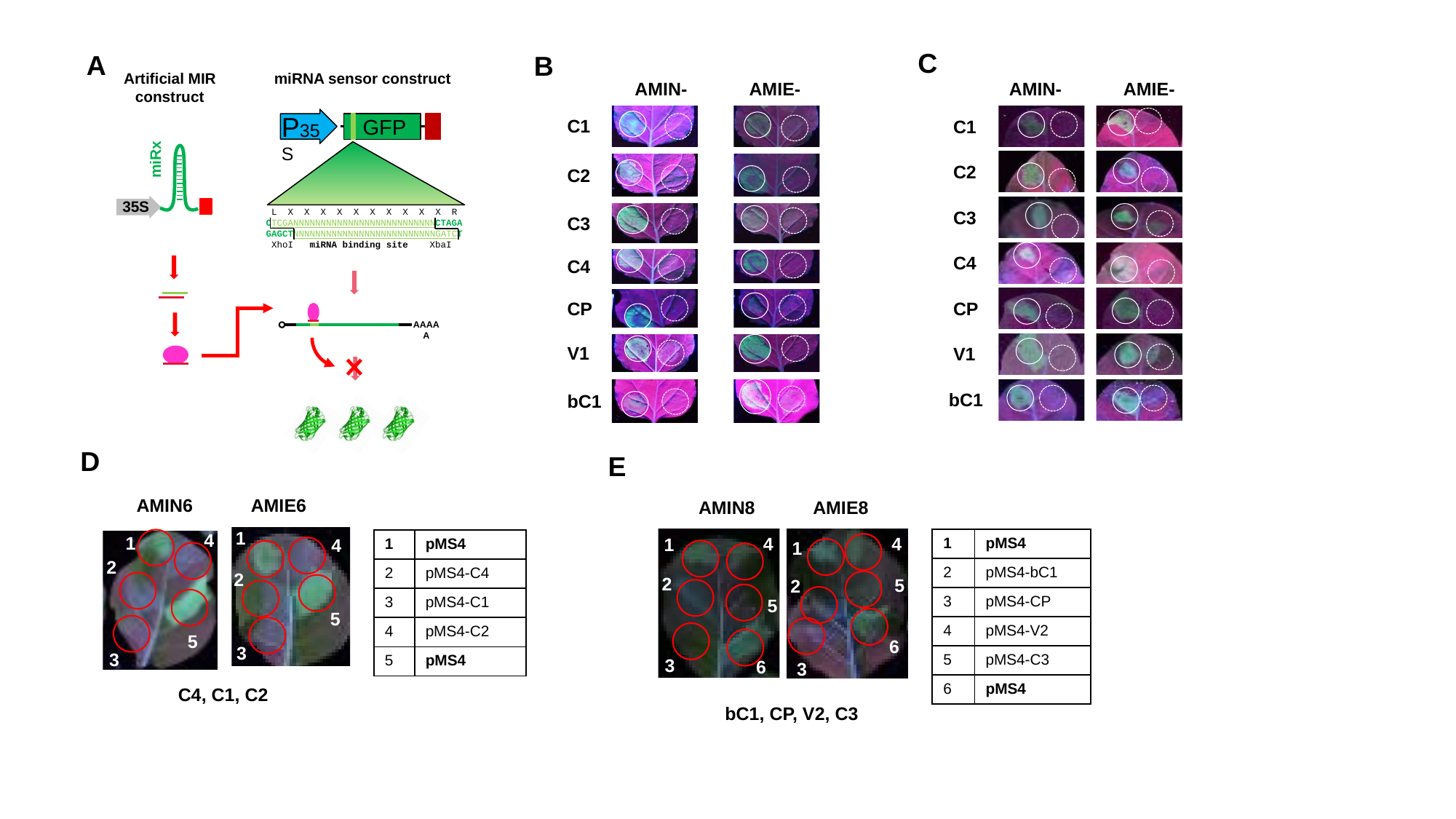

C
A
B
Artificial MIR construct
miRx
35S
miRNA sensor construct
P35S
GFP
 L X X X X X X X X X X R
CTCGANNNNNNNNNNNNNNNNNNNNNNNNNNCTAGA
GAGCTNNNNNNNNNNNNNNNNNNNNNNNNNNGATCT
 XhoI miRNA binding site XbaI
AAAAA
AMIN-
AMIE-
C1
C2
C3
C4
CP
V1
bC1
AMIN-
AMIE-
C1
C2
C3
C4
CP
V1
bC1
D
E
AMIN6
AMIE6
1
4
1
4
2
2
5
5
3
3
AMIN8
AMIE8
4
4
1
1
2
5
2
5
6
3
6
3
| 1 | pMS4 |
| --- | --- |
| 2 | pMS4-bC1 |
| 3 | pMS4-CP |
| 4 | pMS4-V2 |
| 5 | pMS4-C3 |
| 6 | pMS4 |
| 1 | pMS4 |
| --- | --- |
| 2 | pMS4-C4 |
| 3 | pMS4-C1 |
| 4 | pMS4-C2 |
| 5 | pMS4 |
C4, C1, C2
 bC1, CP, V2, C3

## Slide 9
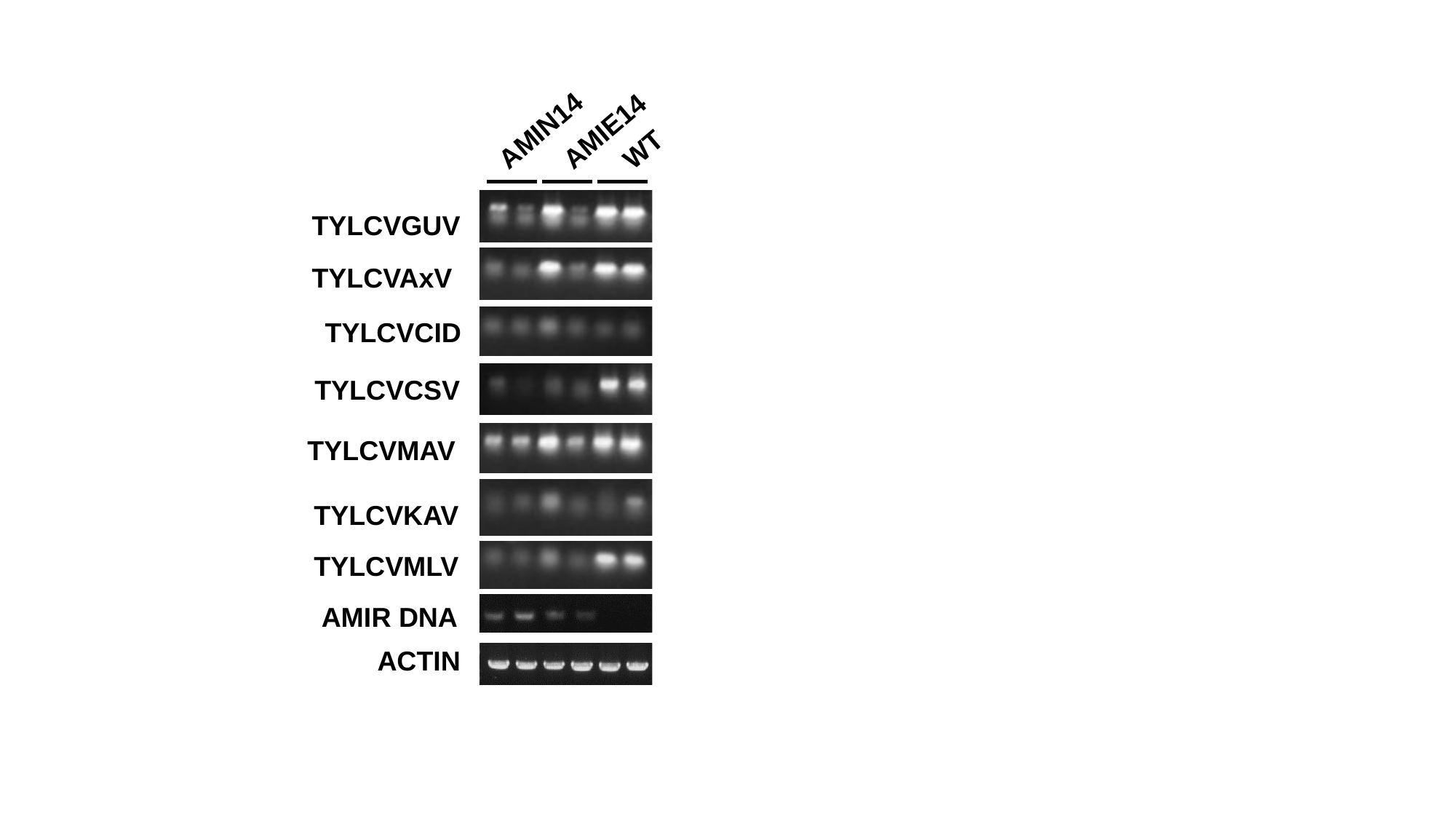

AMIN14
 AMIE14
 WT
TYLCVGUV
TYLCVAxV
TYLCVCID
TYLCVCSV
TYLCVMAV
TYLCVKAV
TYLCVMLV
AMIR DNA
ACTIN

## Slide 10
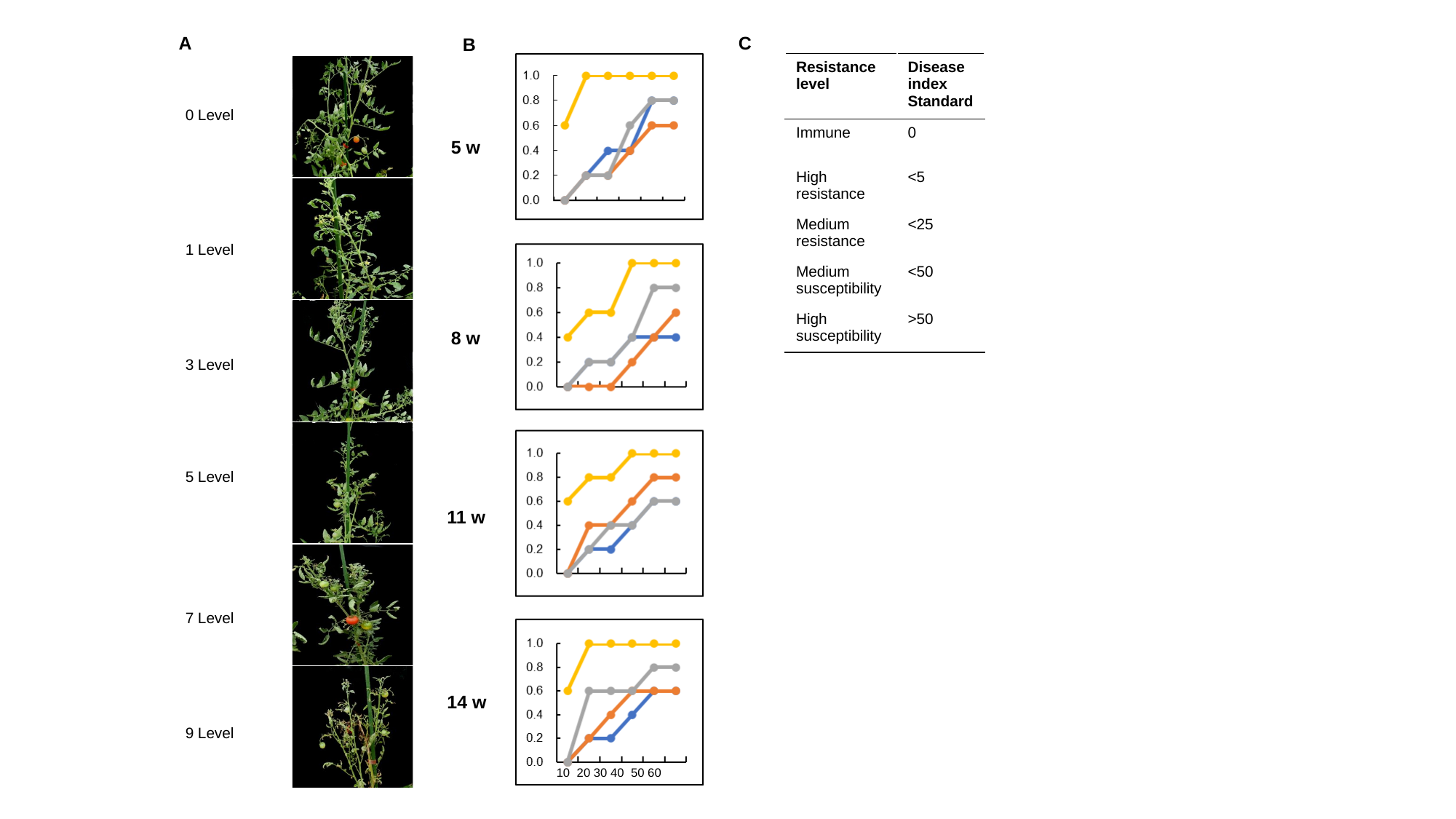

A
C
B
| Resistance level | Disease index Standard |
| --- | --- |
| Immune | 0 |
| High resistance | <5 |
| Medium resistance | <25 |
| Medium susceptibility | <50 |
| High susceptibility | >50 |
0 Level
5 w
1 Level
8 w
3 Level
5 Level
11 w
7 Level
14 w
9 Level
 10 20 30 40 50 60
